# The Wise Mind Balances the Abstract and the Concrete

**DOI:** 10.1162/opmi_a_00149

**Published:** 2024-06-28

**Authors:** Igor Grossmann, Johanna Peetz, Anna Dorfman, Amanda Rotella, Roger Buehler

**Affiliations:** Department of Psychology, University of Waterloo, Waterloo, ON, Canada; Psychology Department, Carleton University, Ottawa, ON, Canada; Department of Psychology, Bar-Ilan University, Ramat Gan, Israel; Department of Psychology, Northumbria University, Newcastle upon Tyne, United Kingdom; Psychology Department, Wilfrid Laurier University, Waterloo, ON, Canada

**Keywords:** mental representations, construal, wisdom, perspective-taking, measurement

## Abstract

We explored how individuals’ mental representations of complex and uncertain situations impact their ability to reason wisely. To this end, we introduce situated methods to capture abstract and concrete mental representations and the switching between them when reflecting on social challenges. Using these methods, we evaluated the alignment of abstractness and concreteness with four integral facets of wisdom: intellectual humility, open-mindedness, perspective-taking, and compromise-seeking. Data from North American and UK participants (*N* = 1,151) revealed that both abstract and concrete construals significantly contribute to wise reasoning, even when controlling for a host of relevant covariates and potential response bias. Natural language processing of unstructured texts among high (top 25%) and low (bottom 25%) wisdom participants corroborated these results: semantic networks of the high wisdom group reveal greater use of both abstract and concrete themes compared to the low wisdom group. Finally, employing a repeated strategy-choice method as an additional measure, our findings demonstrated that individuals who showed a greater balance and switching between these construal types exhibited higher wisdom. Our findings advance understanding of individual differences in mental representations and how construals shape reasoning across contexts in everyday life.

## INTRODUCTION

For centuries, scholars, leaders, and spiritual figures have pondered the elusive qualities that make up a wise mind. Take Isaiah Berlin’s “hedgehog and the fox” analogy (Berlin, 1953), which distinguished between thinkers favoring a monist, abstract approach as a basis for their judgment, and thinkers favoring a pluralist and context-sensitive approach. Berlin categorized various poets and luminaries as either abstract “hedgehogs” (e.g., Plato, Hegel, Dostoyevsky, or Nietzsche) or pluralist, context-aware “foxes” (e.g., Aristotle, Franklin, Pushkin, or Diderot) who draw on both the abstract and the concrete to move on “many levels, seizing upon the essence of a vast variety of experiences.” In many areas of life imbued with uncertainty (Keil, 2010) such as geopolitics (Tetlock, 2005), one may take a step back and approach an issue abstractly or consider how to balance abstract analytical principles with concrete features of the situation at hand (Brunswick, 1955; Hammond, 2010).

Critically, the distinction between abstract and concrete thinking remains a topic at the heart of several programs of research in the social (Trope & Liberman, 2010) and cognitive sciences (e.g., Barsalou et al., 2018; Bolognesi et al., 2020; Borghi, 2022). In social psychology, the influential (Adler & Sarstedt, 2021) Construal Level Theory (CLT) posits that psychological distance from events and objects influences the nature of their mental representation (Trope & Liberman, 2010). Per CLT, the present experience is represented concretely, whereas events and objects that are psychologically distant are represented abstractly. In contradistinction to the pluralistic approach of foxes outlined above, some interpretations of the CLT framework imply abstractness and concreteness lie on the opposite ends of a continuum, exemplified by the language of “high” and “low” levels of construal in this scholarship (Burgoon et al., 2013) and the common use of the unidimensional Behavioral Identification Form (BIF; Vallacher & Wegner, 1989) to assess effectiveness of the manipulated construal. Practically, scholars typically assess abstract and concrete modes of thinking on a unidimensional continuum. That is, studies usually compute a general abstractness index from concreteness and abstractness scores rather than use each score separately, with the assumption that “high” abstractness is merely an inverse of the “low” concreteness.

Conversely, abstractness and concreteness can also be understood as complementary processes in a multidimensional space (Bolognesi et al., 2020; Borghi, 2022; Borghi et al., 2018; Troyer & McRae, 2022; Villani et al., 2022): From the situated perspective on mental representations (Barsalou et al., 2018), abstract processes are posited as necessary to integrate information to comprehend what is happening in a concrete situation, to make predictions about how a situation may unfold and change, or to select actions that are most likely to yield desired outcomes. From this perspective, mental representations help to identify situational elements and to integrate them; both steps could include elements of abstractness and concreteness.

Similarly, the recently advanced regulatory scope theory in social psychology (Trope et al., 2021) posits individuals modulate between abstract and concrete mental representations, not solely based on their regulatory focus or the psychological distance of a task, but also in response to the situational demands and the need for regulatory balance. Abstract construals, which facilitate a broader, expansive scope, are typically applied to tasks or goals that are psychologically distant or future-oriented. In contrast, concrete construals, which lead to a more contractive scope, are employed for immediate, specific tasks or problems. Crucially, effective regulation, as proposed by this theory, involves the capacity for flexible switching between these construal types. This adaptability is essential for responding effectively to varying contextual demands and for maintaining a balance between the expansive and contractive aspects of regulatory scope–a novel proposition calling for empirical research.

Overall, in spite of rich theorizing of abstract and concrete modes of thinking as distinct processes within both cognitive science (e.g., Barsalou et al., 2018) and social psychology (e.g., Trope et al., 2021), dominant measurement approaches aim to capture abstractness and concreteness along a single continuum. Putting these insights together, we investigate the relationship between wisdom and abstract and concrete construals. Because we observed measurement issues in prior construal research (see Supplement), we introduce and psychometrically validate a novel situation-sensitive method to capture abstract and concrete construal. Following recent theoretical propositions for complementarity of abstract and concrete construal (Barsalou et al., 2018; Trope et al., 2021; Wiesenfeld et al., 2017; also see Steinbach et al., 2019), we further explore construal switching. To examine the association of mental representations to the quality of one’s thought, we tested the association of abstractness to concreteness and the role of each construal type for several cardinal features of wisdom—intellectual humility, recognition of change, perspective-taking, and compromise-seeking.

### On Wisdom

Wisdom is a complex network of mental features that extends beyond knowledge or domain-specific cognitive abilities (Baltes & Kunzmann, 2004; Baltes & Smith, 2008; Baltes & Staudinger, 2000; Darnell et al., 2019; Jeste et al., 2010; Kekes, 1983; McKee & Barber, 1999; Sternberg, 2007; Vervaeke & Ferraro, 2013). In recent years, social and behavioral scientists have identified several mental features that form the psychological foundations of wisdom (Baltes & Smith, 2008; Bangen et al., 2013; Grossmann, 2017; Oakes et al., 2019; Santos et al., 2017), such as intellectual humility (i.e., recognition of limits of one’s knowledge), open-mindedness to multiple ways an issue might unfold and change, consideration of different perspectives on the issue, and a search for compromise in resolving opposing viewpoints. These meta-cognitive features are at the center of the recently advanced Common Wisdom Model—i.e., a common denominator across most operationalizations of the construct in behavioral and social sciences (Grossmann, Weststrate, Ardelt, et al., 2020; Grossmann, Weststrate, Ferrari, et al., 2020). These features of wisdom are distinct from other established personality traits and intelligence (Brienza & Grossmann, 2017; Brienza et al., 2018; Grossmann et al., 2013) and have been uniquely associated with prosocial attitudes and behavior (Brienza et al., 2018, 2021; Grossmann & Brienza, 2018; Grossmann et al., 2017), interpersonal and subjective well-being (Grossmann et al., 2013; Huynh et al., 2016; Peetz & Grossmann, 2021), and affective accuracy about future interpersonal conflicts (Grossmann et al., 2021). However, a critical question remains unresolved: How do the mental representations of events in people’s lives relate to these features of wisdom?

### Wisdom and Construal

When people think about events or decisions, their “construals” or mental representations can be either abstract, focusing on general, context-independent characteristics, or concrete, detailing specific, context-dependent aspects (Vallacher & Wegner, 1989). While there is no universal agreement about the defining features of construal, core markers include linguistic categories associated with abstract versus concrete thought (Semin & Fiedler, 1988; Wakslak et al., 2014; Yin et al., 2022), whether people identify the event with abstract versus concrete descriptors (Fujita et al., 2006; Vallacher & Wegner, 1989), whether people focus on how versus why the event occurred (Freitas et al., 2004; Trope et al., 2021; Villani et al., 2022), whether the event is seen as unique versus one of a set of similar experiences (Kahneman & Lovallo, 1993; Lagnado & Sloman, 2004; Ledgerwood et al., 2010; Liberman et al., 2002). Across most of these indicators, construal in social psychology is measured along on a continuum, with abstract thought at one end and concrete thought at the other (Gilead et al., 2020).

This methodological unidimensionality stands in contrast to Berlin’s portrayal of wise “foxes” (1953) – i.e., individuals who appear to rely on both abstract and concrete processes in the context of judgment. Emerging cognitive science research also suggests that abstract thinking, while seemingly opposite to concrete thinking, is essential for implementing concrete action plans (Barsalou et al., 2018). Moreover, features described as abstract and concrete in the CLT scholarship may conceptually target different, albeit interrelated dimensions. Specifically, some theories distinguish between perceptual abstractness on the one hand and categorical abstraction (vs. specificity) on the other hand (Bolognesi et al., 2020; Borghi, 2022). For example, “table” and “theory” differ in perceptual abstractness (tangible vs. intangible), while “table” and “furniture” differ in categorical specificity (specific vs. general). Critically, perceptual abstractness is only weakly related to categorical abstraction (Bolognesi et al., 2020). Notably, in prior CLT research abstract construal has been defined in terms of typicality (Kahneman & Lovallo, 1993; Ledgerwood et al., 2010) and the broader meaning of the situation (e.g., focus on “why”) (Fujita et al., 2006), whereas concrete construal has been defined in terms of experiential, detailed, vivid, readily observable characteristics (e.g., focus on “how”) (Burgoon et al., 2013). These different foci are thought to engage distinct cognitive processes: categorical abstraction (vs. specificity; for abstract construal) and perceptual concreteness (vs. abstractness; for concrete construals).

These observations lead us to question how abstract and concrete construals relate in the context of wisdom, especially given the mixed findings in prior research. On the one hand, some scholarship shows that ego de-centering—a strategy implicating psychological distance (Kross & Ayduk, 2017)—can promote greater wisdom (for a review, see Grossmann, 2017), including intellectual humility, consideration of different ways a complex situation may unfold, and willingness to compromise (Grossmann et al., 2021; Grossmann & Kross, 2014; Kross & Grossmann, 2012). Per CLT, these findings suggest that greater wisdom is associated with more abstract thought. On the other hand, several observational, diary, and experimental studies have documented a positive association between aforementioned features of wisdom and attention to emotional experiences and emotional intensity (Grossmann et al., 2016, 2019, 2021) – both considered concrete features of an experience. Moreover, abstract thought can detract from wisdom, such that abstract construal can lead to oversimplified, schematic (Aguilar et al., 2013) or stereotypical (McCrea et al., 2012) thinking, and ignore unique, individuating features of the situation (Braga et al., 2014; Eyal et al., 2011) that could benefit wise judgment.

Thus, the question remains: how do abstract and concrete construal relate to wisdom? Bringing social and cognitive science insights together, we propose that abstractness and concreteness have complementary benefits for wisdom, and that the optimal approach may be to balance or switch between them. This aligns with the idea that wisdom is pluralistic (Brunswick, 1955; Dhami & Mumpower, 2018; Hammond, 2010), involving a balance between abstract and context-specific elements of judgment to maximize adaptive outcomes. Moreover, it aligns with the cognitive science view on the complementarity of abstract construal (or categorical abstraction) and concrete construal (or perceptual concreteness) in mental representations.

We suggest that though mental features of wisdom may be aligned with an abstract construal of the situation, there are also potential unwise consequences of solely relying on representations that may be oversimplified or schematic. Focusing also on concrete representations may be helpful for promoting wisdom because they allow people to be sensitive to the details of the here and now, and respond to unique, individuating information available in the current context. We propose that mental features of wisdom may be aligned with a consideration of both abstract and concrete mental representations. Following the pluralist style of thought Berlin attributed to intellectual “foxes” (Berlin, 1953), features of wise thought such as intellectual humility or perspective-taking may be aligned with greater flexibility in mental representations.

To elaborate on this proposition, consider such aspects of wisdom as intellectual humility (Porter et al., 2022). Recognizing limits of one’s knowledge requires focusing on specific details of the issue at hand (concrete construal) and understanding one’s general knowledge scope (abstract construal). In a similar vein, acknowledgment of different perspectives requires awareness of one’s perspective and concrete perspectives of others, which at the most basic level requires concrete construal. But it also requires a more abstract realization whether one’s perspective is like others. Many meta-cognitive aspects of the Common Wisdom Model discussed in philosophy and behavioral sciences such as intellectual humility, acknowledgement of uncertainty, and perspective-taking (Grossmann, Weststrate, Ardelt, et al., 2020) appear to be related to both concrete and abstract construals. This proposition also dovetails with research on human judgment, which shows that a context-sensitive fox, who draws on both the abstract and the concrete, is more likely to correctly forecast geopolitical events compared to an abstract-only hedgehog (Mellers et al., 2015).

### Psychometric Concerns and Level of Analysis

To test this proposition, it is crucial to start by examining the psychometric properties of mental construals. Existing trait-style construal measures such as the Behavioral Identification Inventory (BIF; Vallacher & Wegner, 1989) imply that construal is a unidimensional construct with abstractness on one end and concreteness on another; thus, the relationship between the subjective sense of abstractness and concreteness would be negative and there would be little reason to consider their mutual association with one’s wisdom. However, if people mentally represent abstractness and concreteness as unique dimensions, the relationship between them can take different forms^1^.

Moreover, it is important to note that while the current literature on construal has mainly focused on experimentally-induced changes in a given situation (for example Fiedler et al., 2012), the relationship between abstract and concrete construal may also vary depending on the level of analysis (Keil, 2010; Vallacher & Wegner, 1989). *Between-group* differences in construal in experiments (e.g., more abstract mental representations in the distanced group compared to less distanced group)—as studied in classic between-person social psychological experiments—do not necessary correspond to the *inter-individual* differences in construal (e.g., individuals who show more abstract mental representations might not also report greater psychological distance, as studied in cognitive science scholarship on abstract and concrete concepts). Nor would either of these levels of analysis need to correspond to the level of *intra-individual* change (e.g., if the individual changes degree of abstractness from one situation to another, they might not show a corresponding change in psychological distance)—a so far underexplored area of research. For example, while individuals may vary in their general tendency to use abstract or concrete construals, the relationship between these construals may also differ across social contexts. To overcome these limitations, we examine the relationship between abstract and concrete construal at both the inter-individual and intra-individual levels to advance our understanding of how construal is implicated in making wise judgments.

### Overview

A series of pilot studies revealed a positive association between reports of abstractness and concreteness and wisdom in reflections on autobiographical events and decision scenarios. Based on this pilot work, in Study 1 we developed and psychometrically evaluated a novel individual difference measure of abstractness and concreteness and their influence of the central features of wisdom. In Study 2, we used this new measure to examine how abstract and concrete construals distinctly align with mental features of wisdom across different levels of analysis; between-person level and when examining within-person variability in construal and wisdom across a range of social situations. We examined these associations across social situations in which people had experienced a conflict or some other challenge involving another person; the kinds of real-life circumstances where wise judgment is advantageous. Additionally, we examined the extent to which people switch their construal strategies from abstract to concrete while reflecting on challenging issues and how construal switching aligns to mental features of wisdom across different levels of analysis. Our results suggest that people represent abstract and concrete construals as distinct processes rather than inverse poles of the same dimension. Moreover, we observed that both abstract and concrete construals were each positively associated with each tested feature of wisdom (Studies 1–2), as were measures of balance and switching between construal types (Study 2). The pre-registration documents, unabridged surveys, and data for all studies are available in the supplemental online materials.

## PILOT STUDIES

We started by examining three features reported in prior construal research: 1) action representation – asking *why* an action is performed (abstract) versus *how* an action is performed (concrete) (Fujita et al., 2006; Villani et al., 2022); 2) mental model of an event – thinking about an event as an instance of a broader category (abstraction) versus as a unique instance (specificity) (Bolognesi et al., 2020; Ledgerwood et al., 2010); and 3) the event viewpoint – observer (perceptual abstractness) versus experiential actor (perceptual concreteness) (Bolognesi et al., 2020; Libby & Eibach, 2011; Shaeffer et al., 2015). In four studies, we asked participants to report these construal-related features when reflecting on events in their lives, which included autobiographical events, anticipated social challenges, and decision scenarios. Subsequently, participants completed an established scale assessing mental features of wisdom (Brienza et al., 2018; Grossmann, Weststrate, Ardelt, et al., 2020). In Pilot Study A we found that both features of abstract and of concrete construal were independently linked with wiser reasoning (intellectual humility, open-mindedness to change, perspective-taking, search for a compromise; examined separately and jointly) about anticipated future social situations. Pilot Study B replicated these links with different features of construal, focusing on autobiographical past situations. In Pilot Study C we unsuccessfully attempted to manipulate the prevalence of construal features (Freitas et al., 2004), but again showed that, on the level of individual differences, both abstract and concrete features were independently linked with wiser reasoning. Pilot Study D examined hypothetical decision scenarios and showed that, in line with previous CLT research (Eyal et al., 2004; Liberman & Trope, 1998), features of abstractness also uniquely related to desirability concerns and features of concreteness to feasibility concerns, supporting the discriminant validity of the construal features; in turn, both abstract and concrete construal features were associated with wiser reasoning.

In line with pre-registered predictions, the results from each pilot study along with the meta-analytic estimates (overall *N* = 915) showed that both abstract and concrete construal were positively associated with mental features of wisdom, abstract: *r* = .23, 95% *CI* [.17; .29]; concrete: *r* = .28, 95% *CI* [.22; .37]. The effects were consistent across different features of construal, and when controlling for length of reflection (a proxy for deliberation effort). Detailed results are presented in the online supplement.

However, analyses of the features of abstract and concrete construal used in these pilot studies also revealed that the construal indices showed modest reliability. These concerns motivated us to develop a more theoretically and psychometrically grounded measure of abstract and concrete construal in our Study 1.

## STUDY 1

To overcome the measurement issues presented in the pilot studies, in Study 1 we developed a new *S*ituation-specific *A*bstract and *C*oncrete *C*onstrual *S*cale (SACCS). We examined the underlying factor structure, the nomological network (including convergent and discriminant validity), and associations to wisdom. In the exploratory Study 1a, we performed item selection. In Study 1b, we performed pre-registered confirmatory analyses on two new samples (https://osf.io/gf8hu), reducing the item pool. Simultaneously, we examined the nomological network of the scale and tested the associations to mental features of wisdom. We predicted factor analyses to reveal largely independent factors for abstract and concrete construals. Further, we predicted both abstract and concrete construal would be independently and positively related to wisdom. We examined robustness of the associations between both construal types and wisdom, with cognitive reflection (Frederick, 2005), impulsivity, rational-experiential focus (Norris & Epstein, 2011), and differences in personality (Ashton & Lee, 2009) as covariates.

### Methods

#### Ethics Review Board Statement.

This research was reviewed and received ethics clearance through the Research Ethics Committee (Protocol # 22518). Informed consent was obtained from all participants. This research was carried out following the recommendations of the Human Research Ethics Committee at the University of Waterloo, with written informed consent following the Declaration of Helsinki.

### Item Generation and Initial Selection (Study 1a)

We generated 67 items for abstract and concrete construal that appeared central to prior theory and research (for reviews, see Trope & Liberman, 2010; Vallacher & Wegner, 2012). First, we performed an exploratory factor analysis (EFA) to find the best factor structure as well as identify uniquely-loading items on respective factors. In parallel, we reached out to five construal level theory experts, to ensure our selection was consistent with features construal researchers consider relevant for the construct. Employing a variant of the expert elicitation procedure, experts provided general feedback on key features of abstract and concrete construal, also indicated the suitability of these items for the operationalization of abstract and concrete construal in challenging situations. Through this process, we reduced the item pool to 22 items.

#### Participants.

We recruited a convenience sample of English-speaking mTurk participants via CloudResearch in exchange for $1.20. Four hundred sixty-seven U.S. participants completed the study. We excluded observations that included bot-like (e.g., “nice”) and nonsense responses (e.g., “The situation is very happens to moment”) to the open-ended questions, and participants who failed to respond to most of the items. Further, we used Mahalanobis distance scores to remove multivariate outliers (see supplement for details). The final sample included 293 participants (*M*age = 35.89, *SD* = 11.92; 36% female; 6.83% no college, 25.94% some college/vocational school, 49.49% completed college, 17.75% graduate/professional degree; *Md*[household income] = $35,001–$50,000; 5.80% Asian, 17.41% Black, 68.26% White, 5.80% Hispanic, 0.68% East Indian, 1.02% Mixed).

#### Procedure and Materials.

Because we sought to situate the measure of construal in the context of specific states, we first asked participants to recall a social conflict or some other challenging recent situation that has happened to them with another person (e.g., disagreement) (Brienza et al., 2018; see Appendix for verbatim instructions). One of the significant challenges with recall tasks are the memory bias and desirability-related distortions (Kahneman et al., 2004; Schwarz et al., 2009). To ensure greater access to memory, we asked participants to reconstruct the event and contextualise the details by answering six questions regarding the event (e.g., “When did the situation first begin?;” “Where were you when the situation happened.”) Then, participants reflected on that situation and described their thoughts and feelings.

We instructed participants to continue thinking about the challenging situation they described and to report what they did as the experience unfolded. Participants subsequently responded to 67 construal-related items on a 5-point scale (1 = *not at all*, 3 = *somewhat*, 5 = *very much*). We designed 34 items for abstract construal (e.g., “I focused on the broader meaning of the situation”) and 33 items for concrete construal (e.g., “I focused on the specific details of the situation”). The complete list of items is presented on the OSF project page. Due to a technical error two abstract and one concrete item were presented twice in the survey. The repeats were deleted prior to further analyses. To reduce scrolling fatigue when filling out the questionnaire online, items were spread across four pages. For each page, we randomly selected half of the items from the theorized abstractness pool and others from the concreteness pool. The abstract and concrete items assigned to each page were identical across participants, whereas pages and order of items in each page were randomized to avoid order effects. After completing the construal measure, participants provided demographic information such as age and biological sex.

#### Analytical Procedure.

The complete analytic protocol, including each step of the item reduction procedure, is presented in the online supplement (Tables S9–S14). We used conventional criteria to evaluate model fit (standardized root mean square residual [RMSR] < .10, root mean square error of approximation [RMSEA] < .08, comparative fit index [CFI] > .90) (Hu & Bentler, 1999). We performed EFA via *psych* (Revelle, 2022) package in *R*, using the default Ordinary Least Squares to find the minimum residual solution. We identified the number of factors by inspecting eigenvalues, scree plot, and performing parallel analyses. This process suggested four factors. Thus, we imposed a 4-factor solution. Because we were agnostic about the possible association of factors, we used oblique (oblimin) rotation to allow the factors to correlate. At each iteration, we removed items that did not load strongly onto a single factor (i.e., coefficients < .30), or that cross-loaded substantially on more than one factor (<.20 difference between loadings on different factors). We repeated this process several times, at each point inspecting model fit and cross-loadings until no low- and ambiguously-loading items remained.

#### Expert Elicitation.

In parallel to the factor analytic methods to reveal the items that uniquely identified (loaded onto) the abstract/concrete construal factors according to lay participants’ responses, we reached out to experts who provided their feedback on the fit of items to the theoretical concepts of abstract and concrete construal. Five experts in social psychology who have extensively published on construal level theory provided feedback on the appropriateness of the initial list of 67 items for measuring abstract and concrete construal when reflecting on challenging experience (i.e., the nature of the recall task) and to indicate ten items to represent abstractness and concreteness, each. The final selection included 14 items identified in the initial factor analyses (but without distance and emotion items), supplemented with 8 items that received high scores from experts and showed no cross-loadings in initial factor analyses.

### Model Selection and Nomological Network (Study 1b)

In the pre-registered phase, we had three aims. First, we refined and confirmed the factor structure of the SACCS on a new sample of English-speaking North Americans. Upon initial evidence of a poor fit, we trimmed items further and compared different models of abstract and concrete construal. To ensure positive association between abstract and concrete construal is not due to a general response or cognitive reflection tendency, we included a bifactor model. Moreover, in analyses with covariates, we considered a range of measures capturing domain-general cognitive reflection, need for cognition, experiential and analytical thinking, personality, and other relevant situational and trait-level individual differences outlined below. Subsequently, we recruited a new sample from a different English-speaking culture – the United Kingdom – to test the generalizability of the model we identified.

#### Participants.

We recruited two English-speaking convenience samples from two different geographic regions. First, following our pre-registered protocol we targeted 314 American and Canadian residents from MTurk via CloudResearch, in exchange for $3.25. This target sample is sufficient to detect a moderate to small effect size in personality/social psychology (*r* = .20), with 95% power (per G*Power calculation). Given the prevalence of bot-style responses on the MTurk platform, we oversampled by 20%, requesting data from 375 participants. Because some participants did not finish the study, we included 433 participants with partial submission (at least 2/3 of the study). Based on pre-registration, we excluded 110 participants who provided non-sense responses to open-ended questions or wrote texts of no relevance to the open-ended questions (e.g., “Very good study” in response to the question about the nature of the recalled social interaction), and one participant who missed half of the construal and wisdom-related items; the resulting exclusion rate of 23% was typical for MTurk studies in the field (Aguinis et al., 2021).

The final MTurk sample included 323 participants (*M*age = 34.83, *SD* = 10.19; 42% female; 7.76% no college, 22.36% some college/vocational school, 52.48% completed college, 17.39% graduate/prof. degree; *Md*[household income] = $50,001–$75,000; 7.45% Asian, 18.32% Black, 67.08% White, 4.35% Hispanic, 0.62% East Indian, 1.24% Mixed), thereby reaching our targeted sample.

To confirm the factor structure and to generalize beyond North America, we targeted another sample of 300 individuals with primary residency in the United Kingdom via Prolific. Participants received 2.6 GBP (approximately $3.25) for participation. We followed the same exclusion criteria as above, excluding 54 responses from participants who did not complete at least 2/3 of the study, and 8 participants who provided incoherent responses to open-ended questions. Further, we built in two attention check items at the end of the impulsivity and rational-experiential focus questionnaires (Please select “1 - Not at all” / Please select “2 - Somewhat disagree”) and excluded another 8 participants who did not follow these instructions. The final Prolific sample included 238 participants (*M*age = 32.92, *SD* = 10.96; 76% female; 16.39% no college, 29.83% some college/vocational school, 32.77% completed college, 21.01% graduate/prof. degree; *Md*[household income] = $35,001–$50,000; 9.28% Asian, 2.53% Black, 81.86% White, 1.27% Hispanic, 1.27% Middle Eastern, 0.84% East Indian, 1.69% Mixed).

#### Procedure and Materials.

Instruction for the North American and UK samples was identical, except for two attention check items included for the UK sample. The instructions for recall and reconstruction of a challenging social event were the same as in Study 1a. Participants completed the reduced set of SACCS items on a 5-point scale (1 = *not at all*, 5 = *very much*; see Figure S5 in the supplement for the distribution of responses).

As participants continued reflecting on the event, we assessed situational appraisals. That is, we measured how people appraised the situation they recalled in terms of *psychological distance* (“I distanced myself from the experience;” “I tried to look at myself through a third-person perspective, as an observer would”),^2^ *temporal closeness to the experience* (“How close in time do you feel to this event?”), *appraisal of closeness to conflict partner* (“How close do you feel to the other person involved in the event now?”), *reliance on emotion/intuition* (4 items, e.g., “I used my heart as a guide for my thoughts”), and *appraisal of impulsivity* (4 items, e.g., “I acted on impulse, without caring what might happen”). All items were rated on 5-point scales (1 = *not at all*, 5 = *very much*).

To capture *mental features of wisdom*, participants completed the Situated Wise Reasoning Scale (SWiS) (Brienza et al., 2018). The reliability of the subscales was good (intellectual humility *α* = .72; multiple ways *α* = .75; other perspectives *α* = .84; search for compromise *α* = .83). As in pilot studies, subscales were also averaged into an overall index of wise reasoning (*α* = .88). SWiS also included four items capturing the consideration of a self-transcendent viewpoint (*α* = .85). Because this facet of the scale is conceptually close to the notion of psychological distance, per expert feedback in Study 1a in our main analyses we treated this facet separately from the rest of the scale. Each result reported in the main text and the supplement remains similar if this facet is included into the composite wisdom score.

Subsequently, participants completed five trait-style individual difference measures to capture the nomological network of abstract-concrete construal and to probe the construal-wisdom association while controlling for these differences (see Table 2 for descriptives and reliability estimates). These five measures were presented in a randomized order. We assessed participants’ *analytic-rational and experiential thinking* using the Rational Experiential Inventory (REI) (Norris & Epstein, 2011). Participants responded to three 10-item subscales for experiential thinking: intuition (e.g., “I trust my initial feelings about people), emotionality (e.g., “My anger is often very intense”) and imagination (e.g., “Sometimes I like to just sit back and watch things happen”) and to the 12-item analytic-rational thinking subscale (e.g., “I enjoy problems that require hard thinking”) on a 5-point scale (1 = *completely disagree*, 5 = *completely agree*).

To measure participants’ *cognitive reflection*, we used the Cognitive Reflection Test (CRT) (Frederick, 2005). Participants answered three open ended questions that assess the tendency to override a strong wrong response alternative. For instance, for the question “A bat and a ball cost $1.10 in total. The bat costs $1.00 more than the ball. How much does the ball cost? (in cents),” n the intuitive answer is 10 cents, but the correct answer is 5.

We measured participants’ *level of the behavioral identification* using the Behavioral Identification Form (BIF) (Vallacher & Wegner, 1989). It assesses individual differences in level (high vs. low) of identification for different actions. Participants responded to 24 items by choosing one of two descriptions of an action. For instance, for “making a list” one could either select “getting organized” (higher level) or “writing things down” (lower level). The individual’s identification level is calculated based on the number of chosen high-level descriptions, with higher scores reflecting higher level identification.

We assessed participants’ *analytic and holistic thinking tendencies* via the Analysis-Holism Scale (AHS) (Choi et al., 2007). It consists of four independent facets that has 6 items in each: (1) interdependent explanation of causal relationship (*α* = .77, e.g., “Everything in the universe is somehow related to each other”), (2) acceptance of contradiction (*α* = .69 e.g., “It is more desirable to take the middle ground than to go to extremes”), (3) holistic locus of attention (*α* = .73, e.g., “The whole, rather than its parts, should be considered in order to understand a phenomenon”), and (4) linear prediction of change (reverse-scored; *α* = .72, e.g., “If an event is moving toward a certain direction, it will continue to move toward that direction”) on a 5-point scale (1 = *completely disagree*, 5 = *completely agree*). Notably, high scores on the linear prediction of change sub-scale endorsed linearity and predictability instead of holism-aligned dialecticism and uncertainty; on other sub-scales higher scores reflected greater holism. Reliability analyses revealed that this sub-scale showed positive (rather than theoretically expected negative) associations to other sub-scales. Critically, even the original psychometric work showed modest-to-negligible association of this sub-scale to several other sub-scales (Table 2 in Choi et al., 2007). Consequently, we focused on the averaged responses to the former three sub-scales concerning interdependent explanation of causal relationship, acceptance of contradiction, and holistic locus of attention, .32 < *r*s ≤ .35, as an index of holistic thinking.

To assess *personality traits*, we used the HEXACO inventory (Ashton & Lee, 2009) to capture six dimensions: Honesty-Humility (e.g., “I wouldn’t use flattery to get a raise or promotion at work, even if I thought it would succeed”), Emotionality (e.g., “I would feel afraid if I had to travel in bad weather conditions”), Extraversion (e.g., “I prefer jobs that involve active social interaction to those that involve working alone”), Agreeableness versus Anger (e.g., “I tend to be lenient in judging other people”), Conscientiousness (e.g., “People often call me a perfectionist”), and Openness to Experience (e.g., “I would enjoy creating a work of art, such as a novel, a song, or a painting”). Participants indicated how much they disagree/agree with each of the 60 items on a 5-point scale (1 = *completely disagree*, 5 = *completely agree*). At the end of the study, participants provided demographic information.

#### Linguistic Markers.

To move beyond self-reported questionnaire responses, we aimed to assess linguistic markers participants spontaneously relied on in their open-ended reflections on the scenarios. Preliminary inspection of open-ended responses indicated sufficiently detailed content for Natural Language Processing (NLP) analyses. The responses exhibited an average length of 22.65 words (*SD* = 19.45, *Range* [5; 163]) and an average of 2 sentences per response (*SD* = 1.03, *Range* [1; 7]), suggesting that the responses were not only varied in length but also rich enough to provide meaningful linguistic data for analysis (also see analyses of co-occurrences below).

We built on the insights from the Linguistic Category Model (LCM) (Semin & Fiedler, 1988)—a framework that categorizes linguistic markers in terms of abstractness and concreteness. The LCM differentiates between concrete language that refers to specific, observable actions or qualities, and abstract language that denotes more general, interpretive, or evaluative states or processes. Informed by this model, we sought to identify markers reflecting experience-focused adjectives and descriptive action verbs to capture concrete language, and markers reflecting person-focused adjectives (e.g., general trait ascriptions) and interpretative action verbs to capture abstract language.

We first part-of-speech classified open-ended text responses via UDPipe English model (https://universaldependencies.org/). Next, we manually inspected adjectives. First, we classified them in terms of characteristics of entities, personal and general attributes that are immutable (e.g., universal, normal, past) and those describing sensory perceptions, feelings and situation-specific nuances (e. g., worried, violent, uncomfortable). This initial classification was guided by ChatGPT-4 suggestions for sorting adjectives in terms of abstractness and concreteness (as of July 14, 2023). Second, to identify descriptive (concrete) and interpretative action (abstract) verbs, we cross-checked UDPipe-tagged verbs against Brysbaert and colleagues’ (2014) concreteness ratings (https://web.archive.org/web/20230925032140/https://crr.ugent.be/archives/1330). To create the descriptive verb dictionary, we excluded transactional and non-descriptive action words like ’communicate’ and ’understand, ’ and focused on the top 200 most common verbs. For interpretive action verbs, we initially examined the least concrete verbs as per Brysbaert et al. ’s ratings. Third, we refined the dictionary of interpretative action verbs by building on the insights from the Linguistic Category Model, selecting verbs related to mental processes, interpretation, evaluation, and judgment. These verbs, typically more abstract, highlight the interpretive and evaluative aspects of social cognition.

For each participant, we calculated the proportion of words in each category relative to the total number of tokens, to control for narrative text length. Final dictionaries are available in the *R* script on the project website (https://osf.io/mwcyp/).

#### Analytical Procedure.

Following our pre-registration, we used the North American sample to estimate the fit of the model identified in Study 1a for the 22 SACCS items. We considered a two-factor model with items from the abstract pool forming one factor and items from the concrete pool forming another factor, while controlling for a common factor. To improve model fit, we followed the pre-registered contingency plan and performed an iterative EFA (see supplement), trimming 3 poor-loading items from abstract and concrete sub-scales, each, and inspecting modification indices to allow for two highest residual correlations. Finally, we used the UK sample to confirm the fit of the bifactor model of this reduced set of 18 items.

In assessing the nomological network, we treated indicators of discriminant and convergent validity as separate yet interrelated components within the same model. We pooled reverse-coded items in the same direction and averaged respective items into indices; descriptive and reliability information for the nomological network indices is presented in Table 2. Our analytical focus was on factor scores derived from a bifactor model, which allowed us to ensure that estimated associations were independent of a general factor affecting responses to abstract and concrete construal items (details on average-based indices of abstract and concrete construal are available in the supplementary analyses in the *R* notebook on OSF).

To test nomological network hypotheses, we used multiple regressions to examine the effects of mean-centered construals and their interaction. Further, for linguistic analyses we only considered responses with at least four words of text to ensure we considered only context-rich responses. This approach was underpinned by several key hypotheses aligned with the tenets of Construal Level Theory (CLT) and related research:

1. Abstractness is more closely associated with psychological distance than concreteness, in line with CLT (Trope & Liberman, 2010).
2. Abstract construals (“big picture” thinking) is more strongly associated with cognitive reappraisal in interpersonal conflicts and interpersonal closeness (Gross, 1998) compared to concreteness.
3. Abstractness aligns with higher attribution of emotionality to past transgressions (Kousta et al., 2011).
4. Concreteness relates to situation-sensitive personality traits like conscientiousness, extraversion, and agreeableness.
5. Linguistically, abstractness is associated with descriptors of entities and trait attributes, while concreteness corresponds with the prevalence of descriptive action verbs and adjectives that detail one’s perceptions and situational specifics (Semin & Fiedler, 1988).

For wisdom-related analyses, we estimated a bifactor model with SACCS items and SWiS items feeding into a general factor. In this model, we regressed a second-order wisdom factor (with first-order factors of intellectual humility, others’ perspectives, multiple ways, and search for compromise) on abstract and concrete construal. Further, we probed these associations when including analytic-rational and experiential thinking, cognitive reflection, and personality traits as covariates. Given the large number of indicators in the nomological network and wisdom-related models, we maximized power by performing analyses across North American and the UK samples. Supplementary analyses report sample-wise analyses.

#### NLP Analyses.

Using part-of-speech tagged tokens (via UDPipe; see above), we identified verbs, nouns, and adjectives in each sentence. We sorted the resulting dataframe in descending order of wisdom factor scores, obtained from the bifactor model with wisdom and SACCS items. Subsequently, we examined co-occurrences between lemmatized verbs, adjectives and verbs in the same sentence of the same participant, separately for top 25% of wisdom performers and bottom 25% of wisdom performers. We plotted the semantic networks based on these co-occurrences, selecting the top 70 co-occurrences to avoid overplotting. Further, we identified overlapping and distinct words in each network for visualization. We aimed to determine whether participants primarily emphasized the concrete details of the negative social conflict they reflected on, as instructed, or also explored broader meanings and positive reappraisals of the experience.

### Results

#### Exploratory Analyses.

The initial item-reduction resulted in 20 items (10 from the abstract pool and 10 from the concrete pool) explaining 47% of the total variance (model fit indices: *RMSR* = .02; *CFI* = 1; *RMSEA* = .001, 95% *CI* [.001 .023]). At this first stage, factors were moderately positively correlated, .14 < *r*s < .59, and no factors showed negative loadings (supplementary Table S13)—i.e., people did not report abstract and concrete experiences along a single continuum.

In the parallel *expert elicitation* stage, we inspected the most frequently expert-nominated items, avoiding items with cross-loadings in initial factor analyses. Many of these items overlapped with the items identified in initial factor analyses (see Table S13 in the supplement). Further, experts converged in their recommendations on treating items reflecting psychological distance and consideration of emotions and intuitions as distinct from abstract and concrete construal. Following this advice, we removed six distance- and emotion/intuition-related items from further consideration.

Integrating factor analytic insights and expert recommendations, we zeroed in on 22 items (evenly split between abstract and concrete pools). The abstract items related to “why” questions, typicality, one’s general character, extrapolation to one’s general future, the “big picture” perspective, and the general significance of the situation. Conversely, the concrete items related to “how” questions, specific behaviors, qualifiers of time, concrete experiences and circumstances, and concrete outcomes of the situation (see Table 1 for verbatim items and sub-scale reliability). The fit of the two-factor model was acceptable (see supplement), *RMSR* = .054, *RMSEA* = .060, and *CFI* = .901. To ensure that the positive association between abstract and concrete construal is not due to a general response tendency, we also tested a bifactor model. This model assumes all items loading onto a general factor, which is orthogonal to factors representing abstract and concrete construal. The model fit of a bifactor model was acceptable, *RMSR* = .047, *RMSEA* = .057, and *CFI* = .924. Here, most abstract and all concrete items significantly loaded on respective factors (Table S14; also see Figure S5 in the supplement). In turn, latent factors of abstractness and concreteness explained non-negligible amounts of variance, abstract: *R*^2^ = .252, concrete: *R*^2^ = .230. Moreover, when controlling for the general factor, abstract and concrete factors remained positively associated with each other, *r* = .48.

**Table 1.** Reliability and standardized parameter estimates for the final items for the Situated Abstract Concrete Construal Scale (SACCS) from bifactor model in Studies 1a–1b.

Item	Study 1a North America		Study 1b North America		Study 1b UK	
	Abstract (*α* = .84)	Concrete (*α* = .88)	Abstract (*α* = .83)	Concrete (*α* = .82)	Abstract (*α* = .76)	Concrete (*α* = .80)
I thought about what the situation says about me as a person. (a1)	.416		.411		.319	
I thought about the life experiences that led to this situation. (a3)	.408		.488		.328	
I thought about how this situation fits into the broader context of my life. (a4)	.400		.479		.467	
I thought about how this situation impacts my overall well-being. (a5)	.273		.466		.387	
I focused on the broader meaning of the situation. (a8)	.246		.593		.307	
I thought about whether this situation was typical. (a12)	.551					
I thought about the situation as one of many similar experiences. (a15)	.774		.796		.567	
It seemed to represent a typical event in my life. (a17)	.722		.688		.904	
I focused on the big picture. (a26)	.202		.411		.262	
I asked myself why this situation made me feel the way it did. (a28)	.407		.458		.196	
I thought about the significance of this situation for people involved. (a31)	.080					
I considered the specific words I was saying in the situation. (c1)		.429		.546		.169
I thought about the specific events and circumstances that led to this situation. (c3)		.400				
I thought about how this situation would immediately impact me. (c4)		.486				
I focused on the specific details of the situation. (c8)		.505		.600		.444
I considered the concrete and immediate outcomes of the situation. (c10)		.326		.413		.326
I focused on each part of the situation as the experience unfolded. (c12)		.491		.533		.402
I focused on the “here and now” of the situation. (c14)		.562		.458		.488
I focused on specific aspects of the experience. (c16)		.482		.606		.574
I focused on what I was saying and doing in the situation. (c22)		.360		.558		.447
I focused on specific behaviors during the situation. (c27)		.369		.654		.560
I considered the specific circumstances surrounding the situation. (c28)		.407		.451		.541

*Note*. Models include a general factor, accounting for common method variance across items. Study 1a includes 22 items. Study 1b includes the final 18 items after further item-reduction analyses, as well as correlated residuals (a1 ∼∼ a28; c1 ∼∼ c22). Item type (a = abstract, c = concrete) and number presented in parentheses, numbers correspond to the items from the original list.

#### Confirmatory Analyses.

**Figure 1.**
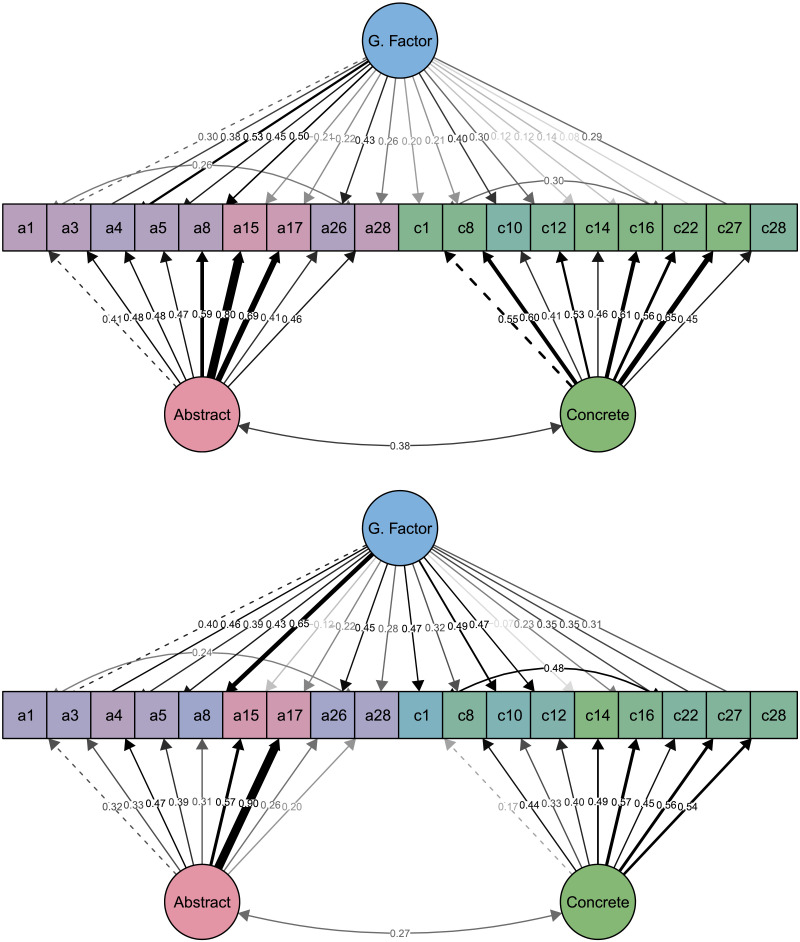
Path diagram of the bifactor model with 18 items (a = items from the abstract pool; c = items from the concrete pool) in Study 1. Top panel –North American sample data. Bottom panel –UK sample data. G. Factor = general factor. Item coloring and line thickness correspond to strength of association with respective construal factors and the general factor in a given sample. Dotted line represents an item with unstandardized factor loading fixed to 1 (a requirement for structural equation modelling analyses). For ease of interpretation, we present standardized parameter estimates. For wording of each item, refer to Table 1.

Initial confirmatory tests of the 22-item SACCS scale revealed model fit slightly below a priori established benchmarks, *RMSR* = .057, *RMSEA* = .071, *CFI* = .863. Therefore, we followed our pre-registered contingency plan and performed iterative EFAs, reducing the scale to 18 items with an acceptable model fit, *RMSR* = .050, *RMSEA* = .059, *CFI* = .923 (see supplementary Table S17). Further confirmatory tests of this bifactor model on another sample from the UK showed an acceptable fit, *RMSR* = .052, *RMSEA* = .054, *CFI* = .924. Results for both samples indicated that abstract and concrete construal factors explained non-negligible variance in responses to SACCS items, North America: *R*^2^ (abstract) = .242; *R*^2^ (concrete) = .384; UK: *R*^2^ (abstract) = .201; *R*^2^ (concrete) = .054. Path models in Figure 1 show standardized parameters for the North American sample (top) and the UK sample (bottom), revealing significant loading of abstract and concrete items on the respective latent factors, along with a non-negligible positive association between these factors.

##### *Nomological Network*.

We examined hypothesized nomological network associations of the SACCS for trait, state, and linguistic markers. We performed multiverse tests involving two approaches to control for general response tendencies across measures—the bifactor and method factor models. Across both approaches, we observed support for the convergent validity of the SACCS, in line the CLT’s theorizing about abstractness being more aligned with psychological distance than concreteness (Trope & Liberman, 2010), the role of abstract (“big picture”) cognitive reappraisal of interpersonal conflicts and interpersonal closeness (Gross, 1998), alignment of abstractness with greater attribution of emotionality (Kousta et al., 2011) for the past transgression, and concreteness with situation-sensitive personality traits (conscientiousness, extraversion, and agreeableness), as well as alignment of abstractness with linguistic descriptors of entities and trait attributes, and concreteness with prevalence of descriptive action verbs (Semin & Fiedler, 1988) and adjectives describing one’s perceptions and situational specifics.

State-level abstractness and concreteness were largely independent of trait-level responses, with all associations in the small-moderate range (Table 2). People who construed events concretely were more likely to report valuing intuition and imagination in their lives (Table 2 and Table S21 for sample-specific analyses), consistent with the idea that concrete construal is linked with focus on first-hand experiences. Also, participants who construed events concretely were more likely to report higher levels of situation-sensitive personality characteristics (conscientiousness, extraversion, agreeableness). Conversely, people who construed their events abstractly were significantly more likely to rely on heuristics (i.e., choosing a spontaneously appealing wrong option on the Cognitive Reflection Test), and were less likely to appreciate systematic deliberation. In North America, concreteness was also associated with first-hand experiences, REI-Emotionality, -Intuition, and -Imagination (see supplement). Contrary to expectations, the BIF was not associated with construing the event more abstractly and showed a negligible positive association with construing the event concretely. In hindsight, the orthogonality of the BIF and SACCS indices can be explained by distinct levels of analysis outlined in the introduction earlier: Whereas BIF captures trait-level reports on *action*-related preferences, SACCS was explicitly designed to capture subjective construals—i.e., situation-grounded reports on abstractness and concreteness in *reflections* on one’s experience.

**Table 2.** Descriptive statistics, reliability, and associations of nomological network measures to abstract and concrete construal in Study 1.

Measure	Descriptives	Reliability	Abstract	Concrete	A × C
	*M* (*SD*)	*α* (*r*)	*β*	*β*	*β*
*Trait-level measures*					
REI - Analytic (*n* = 12)	3.61 (0.72)	.86	−.13***	.11*	−.01
REI - Intuitive (*n* = 10)	3.39 (0.56)	.70	.05	.06*	.04
REI - Imagination (*n* = 10)	3.74 (0.64)	.78	.05	.16***	.01
REI - Emotionality (*n* = 10)	3.31 (0.57)	.62	.02	0.04	.02
Cognitive Reflection (*n* = 3)	1.30 (1.24)	.79	−.21***	−0.06	.03
Behavioral Identification Form (*n* = 25)	0.60 (0.23)	.87	−.001	.02*	.01
Holistic Thinking (*n* = 3)	4.97 (0.67)	.60	0.06	.17***	.08***
HEXACO - Honesty/Humility (*n* = 10)	3.40 (0.73)	.74	−.11***	−.02	−.05
HEXACO - Emotionality (*n* = 10)	3.30 (0.71)	.77	.01(.03)	.03	.01
HEXACO - Extraversion (*n* = 10)	3.00 (0.73)	.79	−.04(−.02)	.07*	.03
HEXACO - Agreeableness (*n* = 10)	3.20 (0.69)	.76	−.10**	.09**	.03
HEXACO - Conscientiousness (*n* = 10)	3.60 (0.71)	.79	−.17***	.11**	.01
HEXACO - Openness (*n* = 10)	3.50 (0.75)	.79	−.06	.12****	−.04

*Situational appraisals*					
Psychological distance (*n* = 2)	2.70 (1.10)	(−.25)	.35****	.12*	.04
Temporal closeness	3.53 (1.15)	–	.26***	.21***	−.03
Closeness to interpersonal transgressor	3.08 (1.54)	–	.31***	.03	.08
Reliance on emotion/intuition (*n* = 4)	3.59 (0.80)	.63	.21***	.15***	−.02
Impulsivity assessment (*n* = 4)	2.91 (1.19)	.86	.33***	−.06	.01

*Linguistic markers* (% of tokens)					
Person-focused adjectives (log)	1.33 (3.00)	–	.22**	−.02	−.07
Interpretative action verbs (log)	6.02 (6.31)	–	−.02	−.05	−.08
Sensory/situational adjectives (log)	8.90 (11.97)	–	−.10	.22*	.29**
Descriptive action verbs (log)	7.80 (.6.73)	–	−.13	.22*	.06

*State-level self-transcendence*	2.86 (1.18)	.85	.30***	.21***	.14**	
*Wisdom* (average of sub-scales; *n* = 4)	3.01 (0.91)	.89	.21***	.26***	.09**	
Intellectual humility (*n* = 4)	2.90 (1.01)	.72	.28***	.20***	.09*	
Others’ perspectives (*n* = 4)	3.02 (1.13)	.84	.23***	.28***	.13**	
Multiple views (*n* = 4)	3.21 (0.95)	.75	.16***	.25***	.05	
Compromise (*n* = 5)	2.90 (1.01)	.83	.15***	.31***	.09*	

*Note*. *n* = number of items in a measure. *β* = standardized parameter estimates from a multiple regression with abstract construal, concrete construal, and their interaction (A × C), with predictors mean-centered and scaled by 1 *SD* prior to creating an interaction term. Abstract and concrete construal are based on factor scores from a bifactor model in Figure 1, controlling for the influence of a common factor.

*** *p* ≤ .001.

** *p* ≤ .01.

* *p* ≤ .05.

Turning to state-specific correlations, abstractness showed a stronger association than concreteness with the appraisal of the event as psychologically distant, in line with CLT. Further, consistent with the notion that an abstract, “big picture” viewpoint affords reappraising the nature of the conflict and provides socio-emotional benefits thereafter (cf. cognitive reappraisal; Gross, 1998; Kross & Ayduk, 2017, for a review), people who construed their events abstractly *during* the conflict were significantly more likely to *later* appraise being close to the person they had conflict with earlier (also see natural-language processing results below).^3^

Furthermore, they were more likely to assess their past behavior in the conflict situation as impulsive, suggesting that abstractness was aligned with the view of the initial response to the transgression as premature (in line with prior emotion regulatory research regulation; Gross, 1998; Kross & Ayduk, 2017). Moreover, abstract and concrete construal were also additively associated with holism, such that greater holistic tendencies were observed among participants reporting both high abstractness and concreteness (see supplementary Figure S7).

Additionally, we observed systematic associations of abstractness and concreteness to linguistic markers (Table 2). While abstractness was uniquely associated with adjectives describing entities and persons that one cannot perceive directly (e.g., main, normal, universal), concreteness was associated with adjectives describing one’s perception of the experience - sensory perceptions, feelings, and descriptions of the specific situation, as well as with descriptive verbs (e.g., listen, speak, cry). Critically, situation-specific abstract and concrete construal scores were independent of the trait-level differences in action identification, indicating unique contribution of the SACCS^4^ beyond trait-level measures.

##### *Construal and Wisdom*.

Replicating pilot study results, both abstract and concrete construal were significantly positively associated with mental features of wisdom (Table 2 and supplementary Figure S7 and Table S20): each aspect of wisdom was significantly more likely to manifest among participants who reported construing the event both abstractly and concretely. These effects remained virtually identical when controlling for trait-level covariates, situational appraisals, and deliberation time on the task (supplementary Table S20).

Next, we ran a bifactor model in which both construal and wisdom-related items contributed to a common factor, to test the latent-level associations of wisdom to abstract and concrete construal, and their interaction (Cortina et al., 2021). Results showed that controlling for a common method factor, abstract and concrete construal significantly contributed to wisdom, abstract construal: *B* = .543, *SE* = .183, *β* = .208, *z* = 3.24, *p* = .001, concrete construal: *B* = .612, *SE* = .144, *β* = .280, *z* = 4.24, *p* < .001. Moreover, we observed an abstract × concrete construal interaction, *B* = .171, *SE* = .083, *β* = .10, *z* = 2.07, *p* = .039. Simple slope analyses revealed a stronger association of abstractness with wisdom when concreteness was also high (+1 *SD*), *B* = .764, *SE* = .196, *p* < .001, compared to when it was low (−1 *SD*), *B* = .422, *SE* = .206, *p* = .040.

##### *Natural-Language-Processing*.

To examine whether the association between construal and wisdom extends beyond self-report questionnaires, we performed exploratory natural language processing (NLP) analyses. First, we examined the linguistic markers related to abstractness and concreteness which were inspired by Semin and Fiedler’s (1988) linguistic category model (see Table 2). However, we failed to observe significant associations of these linguistic markers and self-reported wisdom facets, Spearman’s ρs < .08, possibly because of high skewness and low to no usage of specified terms, reducing variability essential for detecting meaningful associations, person-focused adjectives: *Md* = 0, *Skew* = 2.68; interpretative action verbs: *Md* = 0, *Skew* = 1.45; sensory adjectives: *Md* = .06, *Skew* = 3.03; descriptive action verbs: *Md* = .08, *Skew* = 9.70.

**Figure 2.**
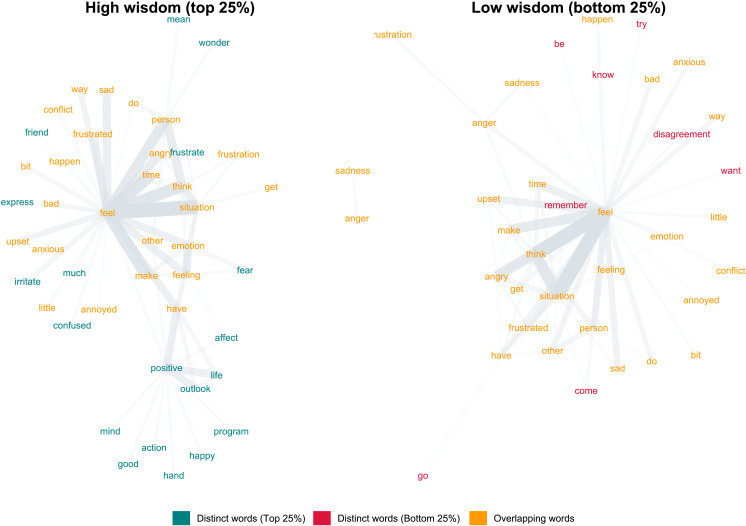
Linguistic analyses of 50 most frequent adjectives, nouns, and verbs in participants’ open-ended reflections on the most recent interpersonal conflict. Visual representation in the network based on strength of cooccurrences (as indicated by thickness of edges in the graph and their spatial position on the graph), standardized across corpora of high and low wisdom performers (indexed by method-factor-adjusted latent score of wisdom). Overlapping words in orange indicate that participants in both groups mentioned concrete feelings involving another person. Distinct words for the high wisdom group indicate that participants in this group simultaneously mentioned the positive outlook on life, an indicator of abstract reframing of the experience in terms of bigger picture (also evidenced by mentioning of *wonder*, *mean*, *mind*, *program*, *action*).

Because of these limitations, in the next step we chose to focus on the corpus-wide (vs. individual-level) analyses comparing high vs. low wisdom groups, probing co-occurrences of key verbs, adjectives, and nouns in the same sentence among the participants scoring in the top 25% and bottom 25% on the SWiS. These analyses reflect semantic dependencies when participants were prompted to write down their “thoughts and feelings about the unresolved interpersonal challenge” they chose to reflect on, prior to providing questionnaire-based ratings of construal. In other words, the instructions prompted participants to be concrete in their reflection, raising the question which group would be more likely to also consider the conflict experience from the abstract big picture angle. The network graphs in Figure 2 revealed that while both groups expressed negative feelings, high wisdom participants also wondered about the situation’s meaning, labelled their emotional states (“confused”), and considered future actions (“program”). Moreover, high wisdom participants appeared to engage in big picture positive reframing of the aversive interpersonal situation (by describing a positive life outlook and reporting positive emotions in their retelling of the conflict), consistent with the notion of cognitive reappraisal (Gross, 1998) – i.e., a mental process involving both specificity/concreteness when bringing to mind the specifics of the situation and abstraction when considering the bigger picture beyond the immediate aversive situation at hand.

Formal analyses corroborated the differential linguistic patterns between high and low wisdom groups. First, Fisher’s Exact Test showed that high wisdom group’s terms were 2.35 times more likely to be unique, 95% *CI* [.94, 7.34], *p*(*OR* > 1) = .065. Moving to terms present in both networks, centrality analysis, based on the count of adjacent edges normalized by *n* − 1 vertices, revealed the high wisdom group had fewer adjacent edges (*M* = .07, *SD* = 0.11) than the low wisdom group (*M* = .12, *SD* = 0.17), Wilcoxon signed-rank test *V* = 59, *p* = .002. This analysis suggests denser discourse clustering around concrete terms about the conflict reflection (e.g., “feel,” “think,” “angry,” “situation,” “frustrated,” “anger;” Figure 2) in the low wisdom group. Finally, co-occurrence frequency was significantly higher in the low wisdom (*Md* = 22) compared to the higher wisdom group (*Md* = 6), as indicated by the negative binomial model, *B* = −1.20, *SE* = 0.08, *z* = 14.33, *p* < .001. Collectively, these findings suggest that the high wisdom group was less inclined than the low wisdom group to dwell solely on the specifics of the immediate situation they reflected upon, integrating more abstract terms into their discourse.

### Discussion

Study 1 introduced a new Situation-specific Abstract and Concrete Construal Scale (SACCS), which showed good psychometric properties and unique associations to mental features of wisdom. The results of Study 1 also suggested that situation-specific reports of abstract and concrete construal tend to go hand in hand, rather than being inverse ends of a single abstract-concrete construal dimension. Additionally, concreteness was more likely to be associated with intuitive and imaginative cognitive styles, whereas abstractness was more likely to be associated with psychological distance and heuristic information processing. Moreover, abstractness and concreteness additively contributed to wisdom. These effects held when controlling for individual difference covariates in reflection styles, personality and analytic-holistic thinking, and when performing multiverse analyses via construal scores from bifactor and common method models of abstractness and concreteness, as well as when modelling associations between construal and wisdom within structural equation modelling (see analyses at osf.io/r6huj).

Critically, the additive effects of abstractness and concreteness on wisdom manifested not only in the SACCS, but also when exploring the semantic content of words in open-ended reflections about the interpersonal conflicts among high and low wisdom groups. Though participants in both groups were focusing on their negative feelings toward the other person (following the prompt instructions to write about the social conflict they recently experienced), high wisdom participants uniquely engaged in big picture positive reframing of the aversive interpersonal situation, which requires balancing the abstract with the concrete (Barsalou et al., 2018; Gross, 1998).

Despite the promising findings of Study 1, however, it is possible that the positive contribution of both high levels of abstractness and concreteness for wisdom is due to the event sampling method: Participants in Study 1 tended to recall concrete, salient events from their lives, and the event reconstruction process focused on details and specifics to avoid memory biases, leading to mostly concrete recalls. It is plausible that under such naturalistic circumstances it is chiefly abstractness, on top of the already concrete event-recall, which is contributing to wiser reflection on the experience. Study 2 addressed this limitation.

## STUDY 2

In Study 2, all participants reflected on the same set of six non-autobiographical scenarios describing challenging interpersonal situations, spread over several study sessions to avoid cognitive fatigue. Study 2 also seeks to systematically evaluate how both abstract and concrete construals contribute to mental features of wisdom across distinct—inter- and intra-individual—levels of analyses. To this end, participants reported on their construal and mental features of wisdom across six situations, spread over three measurement waves. To test effects of construal on wisdom across trait and state levels of analyses, we fit multilevel models with participants’ trait construal tendency across six situations and situation-specific deviations from their trait as predictors of wisdom.

Moreover, Study 2 extended the assessment of construal and wisdom beyond scale-based responses. Inspired by Berlin’s original idea that “foxes” *balance* abstract and concrete construal in their judgment as well as emerging insights about construal balance and flexibility within regulatory scope framework (Trope et al., 2021; Wiesenfeld et al., 2017), we probed how balance and switching between abstract and concrete strategies while working through a complex social event contributes to expression of wisdom in event reflections. Upon rating 18 SACCS items, participants completed the newly developed Strategy Ordering Task: They selected eight of the construal items they were mostly considering in their reflections. We asked participants to rearrange items on the screen via the mouse cursor’s “grab and drag” action to extend measurement beyond scale-based responses. Overall, for each of the six events, we were able to obtain measures of abstract and concrete mental representations both based on rating scales and the Strategy Ordering Task, simultaneously assessing the balance of and switching between abstract and concrete construal strategies while reflecting on a given scenario.

### Methods

#### Ethics Review Board Statement and Pre-Registration.

This research was reviewed and received ethics clearance through the University of Waterloo Research Ethics Committee (#30580). Informed consent was obtained from all participants. All study hypotheses and methods were pre-registered on OSF (https://osf.io/hpd5f).

#### Participants.

We aimed to recruit 300 North American participants for the first measurement wave via Prolific Academic, targeting a final sample of at least 165 participants for the concluding wave. This sample size was determined based on G*Power calculations, indicating it would be sufficient to detect a moderate/small effect size (*r* = .20) in personality/social psychology research, with an *α*/*β* set at 5%. Based on prior research with Prolific samples, we anticipated up to 10% of responses to be excluded due to low quality (suspected bots and nonsense responses) and 30% additional attrition rate between waves. Participants received 2.50 GBP (approximately $2.9) for each wave, in total 7.5 GBP for participating in all three waves over ten days. Participants self-selected into the study and represented a convenience sample.

We started with a sample of 300 participants recruited for the first wave, with 288 continuing to the second wave and 255 completing the third wave. Following our pre-registered protocol, we excluded nonsensical or irrelevant responses (e.g., generic compliments like “very good study”) or bot-like or copy-pasted content from external sources (wave 1 = 1.67%; wave 2 = 4.17%; wave 3 = 5.10%), repeated responses from the same participant (wave 2 = 0.72%; wave 3 = 0.83%), participants who completed less than half of the construal and SWiS items each wave (wave 2 = 1.46%; wave 3 = 0.83%), and participants who showed no variance in construal and SWiS scales (wave 1 = 0.34%; wave 2 = 0.37%; wave 3 = 0.83%). The final sample compromised 297 participants in the first wave and 237 participants in the third wave (see Table 3 for demographics).

**Table 3.** Demographics by Wave in Study 2.

Demographic Variable	Wave 1 (*N* = 297)	Wave 2 (*N* = 270)	Wave 3 (*N* = 237)
Average Age (years)	32.12	32.46	33.04
Percentage Female	53.4	53.93	52.34
No College Education (%)	13.61	12.73	13.62
Some College/Vocational School (%)	35.37	36.7	34.47
Completed College Education (%)	33.67	33.71	34.04
Graduate/Professional Degree (%)	17.35	16.85	17.87
Median Household Income Range	$75,001–$100,000	$75,001–$100,000	$75,001–$100,000
Asian (%)	12.93	12.35	13.62
Black/African American (%)	3.74	3.75	3.4
White (%)	72.11	73.41	71.06
Middle-Eastern (%)	1.36	1.12	1.7
Hispanic/Latino (%)	2.72	2.25	2.55
East Indian (%)	1.02	1.12	0.85
Mixed Ethnicity (%)	6.12	5.99	6.81

#### Procedure.

Participants took part in a 3-wave study with four days between each wave. In each wave, participants were presented with descriptions of two different real events, sampled from reports in prior research (Dorfman et al., 2022). We selected events based on comparable description length, focus on specific (rather than reoccurring) experiences, and their typicality—in prior research, commonly reported challenges concerned social conflicts/arguments with a spouse, friend, or parent (Dorfman et al., 2022). By spreading responses to six events across three waves, we aimed to reduce the burden for a single survey, while simultaneously testing stability of key constructs in different measurement sessions. The presentation order of the two situations was randomized between participants. Below are examples of two situations presented in the first wave (see the supplement for all six stories).

A person at the department where I work is angry at me over something that isn’t my fault. It is actually another person’s fault for not communicating information with them. I feel stuck in the middle and like no one will take responsibility. I just tried to defend myself when the angry person came in and talked about the situation. I explained that the head supervisor had told me that they wanted to communicate with this person. I promised to avoid miscommunications in the future. I feel annoyed and attacked because it’s not my responsibility to play their office politics games.

I got drunk and really upset someone who I am friends with. I guess he thinks I blocked him on Facebook when really, I have deactivated my Facebook account. The following day, I reactivated my Facebook account and tried talking to the person. He is really upset with me. He said he is tired of me doing crazy things whenever I get drunk, and said he is also tired of how emotional I get when I am with him. So, he decided he no longer wants to be friends with me and has blocked me on Facebook. I don’t have his phone number either. So, I can’t text him. I haven’t spoken to him since, and it is really making me sad. I feel like I have lost a best friend because I went from talking to him almost every day to not talking to him at all. I’ve been doing a lot of crying as a result of it. I’ve been trying to talk to the guy, again. Using another account that I have, I am trying to message him on Facebook, but he won’t respond to me.

After reading each story, they were instructed to imagine the event occurring to themselves. Participants then recorded their thoughts and potential actions in response to the situation, as detailed in the supplemental materials. They were also asked to complete the Situation-specific *A*bstract and *C*oncrete *C*onstrual *S*cale (SACCS) and to select up to eight SACCS items that were most relevant to their reflections. Subsequently, participants arranged these chosen items in an order reflecting their thought process. They then completed the Situated Wise reasoning Scale (SWiS, Brienza et al., 2018), which was also used in Study 1. This entire procedure was replicated for a second, different situation. At the end of the session, participants rated their level of identification with each scenario and provided demographic information.

#### Abstract/Concrete Scale.

Participants read the prompt “We would like you to continue to think about the situation as the main person in the story and recall what you considered doing as you reflected on it. Please select the extent to which you engaged in the following thoughts and behaviors. ’As the main person in the story, I… ’” Next, they completed the nine abstract and nine concrete SACCS items identified in Study 1b, on a 1–5 scale (1 = *not at all*, 2 = *slightly*, 3 = *somewhat*, 4 = *moderately*, 5 = *very much*). Because of the nature of the recalled experience, we changed the items from past tense to present perfect tense (e.g., from “I thought about the situation as one of many similar experiences” to “I have thought about the situation as one of many similar experiences.”). Each sub-scale showed very good reliability for each of the events (.72 < abstract *α*s ≤ .88; .85< concrete *α*s ≤ .90). Following the pre-registered analytical plan, we averaged scale-based scores for further analyses.

#### Strategy Ordering Task.

Immediately upon the reflective writing and state-level measures for each scenario, participants read the following prompt: “This is the list of activities you reported engaging in on the previous page. Please select EIGHT activities you were mostly engaging in as the main person in the situation.” Participants first selected items from the list of those they rated engaging with when filling out the SACCS (i.e., items scored above “not at all”). The reason we instructed participants to focus on eight activities was to reduce cognitive load during the subsequent sorting task. We calculated a *disbalance index* between abstract and concrete items by calculating the difference between the relative abstractness score and 50% and subsequently taking the absolute value of this score. Thus, a higher score reflects deviation from parity irrespective of direction.

On the new screen, participants were provided with the following prompt:

As the main person in the story, which of the activities did you consider engaging with first, second, and so on … Take a look at the situation below, if you need a reminder.

Participants were instructed to drag and move the items on their computer screen, in the order they considered them, from the top item being the first to the bottom item being the last. We examined how many times participants switched between abstract and concrete items, treating this score as an index of *construal switching* in their mental representation of the situation. For 12 observations (out of 1,602), participants reported considering fewer than eight construal strategies. To avoid bias in estimation of construal switching, we weighted the scores by the total number of strategies participants selected (i.e., computed proportions out of total selected strategies; ii. multiplied by 7—the maximum number of possible switches).

#### Situated Wise Reasoning Scale (SWiS).

Participants continued to think about the situation as the main person in the story and recall what they considered doing as they reflected on it. Participants completed the same 21-item SWiS questionnaire (Brienza et al., 2018) as in Study 1. The reliability of each facet was good (.67 < intellectual humility^5^ *α*s ≤ .84; .77 < multiple ways *α*s ≤ .86; .78 < other perspectives *α*s ≤ .86; .82 < search for compromise *α*s ≤ .87; .85 < self-transcendence *α*s ≤ .89). Therefore, we created subscale scores by averaging respective items. As in Study 1, we subsequently averaged four subscales into an overall wisdom index (subscale reliabilities: .86 < *α*s ≤ .90; reliability across subscale scores: *α* = .83), treating self-transcendence as a separate measure to avoid conceptual overlap with abstractness.

#### Relatability.

At the end, participants indicated how much they could relate to the story on a 5-point scale (1 = *not at all*, 2 = *slightly*, 3 = *somewhat*, 4 = *moderately*, 5 = *very much*).

#### Analytical Procedure.

Following pre-registration, we examined the reliability of SACCS and SWiS. Because reliability was satisfactory for each scale (*α*s > .70), following pre-registration we averaged items for each scale—this procedure allowed us to compare scores across events ostensibly differing in abstractness and concreteness. We fit a series of random intercept linear mixed effect models with responses nested in participants and in events (thereby treating event as a random factor). Starting with the null models without predictors, we examined intra-individual consistency of each construct across events and measurement waves. Next, we fit a random intercept linear mixed effect model with scale-based abstractness and concreteness scores as predictors of wisdom. Following recommendations for longitudinal designs (Bolger & Laurenceau, 2013), for each predictor we included both trait-level (individual mean across all reported events) and state-specific scores (intra-individual deviation from individual’s mean or person-centered scores). All predictors were mean-centered. We predicted stronger association between construal and wisdom on the state- rather than trait-level of analysis. In the supplementary analyses, we also explored effects for each wisdom-related facet of the SWiS. Finally, we explored associations of construal balance and switching to wisdom.

Next, we focused on disbalance and construal switching scores. First, we examined whether people consistently ranked abstract or concrete items as more accessible and the general tendencies for construal switching. Second, we examined how disbalance and construal switching scores relate to wisdom-related SWiS markers on the trait and state-levels of analyses.

### Results

#### Cross-Situational Consistency.

Participants showed a substantial intra-individual consistency in their construals, on par with consistency of personality (Fleeson, 2001), affect (Oishi et al., 2004), and self-esteem (Diener & Larsen, 2009). That is, participants who reported using abstract [concrete] construals for one event also reported more abstract [concrete] construals for other events. Similarly, there was substantial intra-individual consistency in the mental features of wisdom in our data (see intra-class correlations estimates in supplementary Table S22), such that individuals who reported greater wisdom in reflections on one event also reported greater wisdom for other events. In comparison, measures of construal disbalance and switching showed modest intra-individual consistency (*ICC* = .12 and *ICC* = .14, respectively). In other words, one’s tendency to switch between abstract and concrete strategies when reflecting on a challenging event was less intra-individually consistent (i.e., individuals used different abstract and concrete strategies for different events, measured by the strategy ordering task) compared to consistency of participants’ scale-based reports of abstract and concrete construals (*ICC* = .37 and *ICC* = .51, respectively).

#### Construal and Wisdom.

Replicating Study 1, reports of abstract and concrete construal were both positively associated with greater wisdom in reflections on social conflicts (Tables 3 and Table S22 in the supplement). The additive positive effects of each construal type on wisdom were significant both when examining aggregated traits across events and state-specific variation from person’s aggregates. The latter findings indicate that the results hold on the *within*-person level, when controlling for between-person (trait) differences—greater abstractness and concreteness in a given situation beyond one’s trait average is corresponding to wiser reflection on it. Notably, and in contrast to Study 1, we did not observe significant interactive effects between construal types, suggesting that interactive effects may be less robust compared to additive effects.

Critically, additive effects of both construal types on wisdom were robust when including level of engagement (time spent reflecting and length of reflection essays) or subjective relatability of the event as covariates. Supplementary analyses showed comparable and significant effects for each facet of wisdom, .15 < *β*_trait abstract_ ≤ .29, .17 < *β*_trait concrete_ ≤ .33; .04 < *β*_state abstract_ ≤ .09, .08 < *β*_state concrete_ ≤ .15.

#### Construal Balance and Switching.

Greater balance between abstract and concrete strategies (i.e., the less people deviated from parity between abstract and concrete strategies) among the top eight choices was associated with higher wisdom on a trait level, with a non-significant trend in the same direction on the state-level. Supplementary tests revealed significant positive effects of trait-balance on each facet of wisdom, .16 < *β* ≤ .14, *p*s < .001, and state-balance for intellectual humility, *β* = .04, *p* = .028, and multiple perspectives, *β* = .05, *p* = .026.

**Figure 3.**
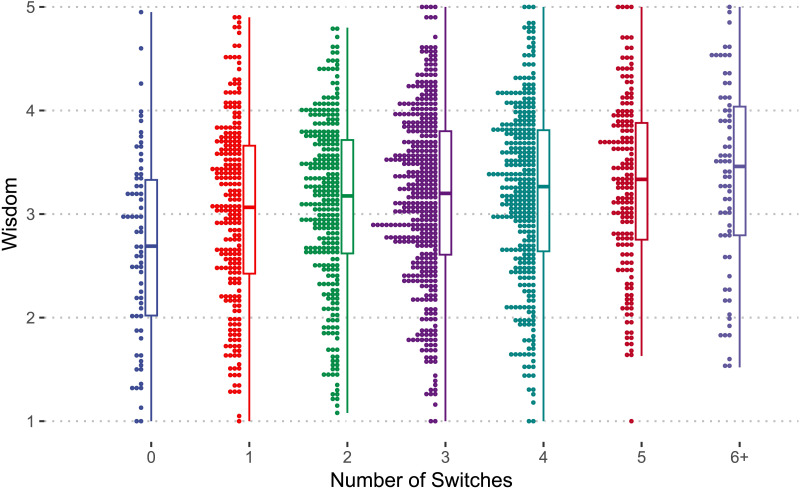
Wisdom scores as a function of number of switches between abstract and concrete construal strategies in Study 2. Box-and-dot-plot depicting distribution and central tendency (median) in each group. Only two participants switched seven times and were binned with the last group. Participants who switched at least once reported greater wisdom compared to those who did not, 2.57 < *t*s ≤ 3.69, .010 < *p* ≤ .001. Also, participants who switched six times reported greater wisdom than participants who switched only once, *t* = 2.05, *p* = .041, or trice, *t* = 1.82, *p* = .069.

Additionally, participants more likely to switch between abstract and concrete construals showed higher wisdom in their reflections (Figure 3 and Table 4^6^). Supplementary tests revealed significant associations of trait-switching to higher scores for each facet of wisdom, .11 < *β* ≤ .16, *p*s ≤ .004. Moreover, entering both disbalance and switching as predictors in the same model indicated independent significant effects of trait disbalance, *β* = −.15, 95%CI [−.22, −.08], *t*(798.37) = 4.01, *p* < .001, and switching, *β* = .08, 95%CI [.02, .15], *t*(796.24) = 2.17, *p* = .030, while no significant effects of state-level scores, .654 < *p*s ≤ .811. Supplementary analyses with level of engagement (time spent reflecting and length of reflection essays) as covariates yield identical results of trait-level balance and switching on wisdom scores, disbalance: −.17 < *β*s ≤ −.16; switching: .13 < *β*s ≤ .14, *p*s < .001.

**Table 4.** Effects of abstract and concrete construal, construal (dis)balance and switching on wisdom on trait and state levels in Study 2.

Level	Covariate	*B*	*SE*	*t*	*df*	*p*	*β*	AIC / BIC
trait	abstract	0.450	0.062	7.25	294.36	< .001	.31	3186.95 / 3240.74
	concrete	0.398	0.063	6.28	294.73	< .001	.27	
	abstract * concrete	0.052	0.062	0.83	285.11	.405	.03	
state	abstract	0.119	0.027	4.44	1304.48	< .001	.09	
	concrete	0.207	0.032	6.38	1308.67	< .001	.12	
	abstract* concrete	0.049	0.045	1.08	1551.11	.281	.02	
trait	disbalance	−0.026	0.005	−5.08	296.87	< .001	−.21	3396.91 / 3429.15
state	disbalance	−0.003	0.001	−1.87	1306.41	.062	−.03	
trait	switching	0.208	0.044	4.72	300.35	< .001	.20	3426.18 / 3458.45
state	switching	0.016	0.012	1.31	1304.13	.190	.02	

*Note*. *N*_observations_ = 1602, *N*_persons_ = 297, *N*_events_ = 6. *β* = standardized coefficients achieved by scaling predictor and dependent variables by 1 *SD* prior to model-fitting. Model fit indices: AIC = Akaike information criterion, BIC = Bayesian information criterion.

### Discussion

Imagine you are planning your first trip to Paris. As you close your eyes, generic images you have seen in countless movies come to mind: the Eiffel Tower, croissants, or perhaps the Louvre Museum. After you open your eyes, you start contemplating specific steps to realize your dream: examining prices when booking your flight, checking the weather to prepare clothes to pack, and studying the city map when reserving the hotel room. The former concerns the answer to the question “why” you may want to go to Paris—i.e., its general desirability. The latter concerns the “how” such a trip may be realized—i.e., the specifics enabling you to get there. These are different ways of thinking about the same thing: one is more abstract, while the other is more concrete (Villani et al., 2022). Which way results in a wiser judgment? We posited that this may be the wrong question. Consistent with recent theorizing in cognitive and social sciences (Barsalou et al., 2018; Bolognesi et al., 2020; Borghi, 2022; Trope et al., 2021; Villani et al., 2022; Wiesenfeld et al., 2017), we proposed that wiser reflections benefit from a combination of *both* abstract and concrete mental representations. To test this idea, we conducted studies involving personal challenges, consumer choices (see supplement), and standardized social conflicts.

In our studies we introduced and validated a method to assess individual differences in construal type: the Situated Abstract and Concrete Construal Scale (SACCS), demonstrating its psychometric and cross-situational utility for measuring mental representations across specific situations in people’s lives. Beyond scale-based responses, semantic networks of words in open-ended reflections among high (top 25%) and low (bottom 25%) wisdom participants revealed greater integration of abstract and concrete concepts (e.g., concrete negative feelings while also mentioning abstract reframing of the experience in terms of bigger picture) in the high wisdom group. We also proposed a metric of construal switching to further extend our work beyond scale-based responses, introducing a task that required participants to choose and rank strategies for reflecting on real-world challenges. Unlike previous questionnaires studies, this task did not rely on scale-based ratings.

Throughout these studies we demonstrated that people represent abstractness and concreteness as complementary rather than mutually exclusive processing modes, and one can employ both toward the same object of construal in their mental representations (Barsalou et al., 2018). We also showed that both abstract and concrete construal jointly contribute to greater wisdom in reflections. Additionally, we observe that people who reported switching between abstract and concrete thought showed greater wisdom in their reflections. This suggests that Berlin’s foxes (Berlin, 1953) who consider many things are more likely to show wise judgment than hedgehogs who focus only on one big thing.

What characteristics afford people who balance and switch between abstractness and concreteness to reason more wisely about social issues? It is tempting to assume that one key reason might involve a more dialectical or open-minded cognitive style among individuals who are switching between abstractness and concreteness. Though seemingly straightforward, this interpretation introduces a possible tautology, as both dialecticism and open-mindedness are core meta-cognitive features of wisdom (for reviews, see Baltes & Smith, 2008; Grossmann, 2017; Grossmann, Weststrate, Ardelt, et al., 2020). Moreover, our supplementary analyses indicate that the relationship between wisdom and balancing abstract and concrete construals is not merely due to overlapping measures. Specifically, effects for balance and switching were similar for the consideration of multiple ways an event may unfold (*multiple views* component—i.e., the defining feature of dialecticism; Spencer-Rodgers et al., 2018) and open-mindedness about others’ viewpoints (*others’ perspectives* component; see supplementary results and Table S22), as they were for other elements such as intellectual humility or the search for compromise and conflict resolution.

Nevertheless, further empirical work is needed to systematically unpack whether the observed association between balancing different construal types and features of wisdom is specific to abstractness and concreteness or if it generalizes beyond that. Future research should investigate whether people spontaneously switch between abstract and concrete modes of thinking and if this switching benefits other features of wisdom. Additionally, experimental designs that manipulate construal switching and assess its impact on wisdom-related outcomes could provide clearer insights into the specific contributions of balancing abstract and concrete construal to wise reasoning.

Our findings have implications for theory about mental representations. They show that mental representations of construal should not be measured along a single dimension with abstractness on one end and concreteness on the other. Instead, one can think about abstract and concrete mental representations as different types of complementary mental features that can exist together in multidimensional space (Borghi et al., 2018) to foster reflective thought (also see Gilead et al., 2020; Wiesenfeld et al., 2017). Consequently, future research on CLT and regulatory scope (Trope et al., 2021) should check if observed effect is caused by higher abstractness and lower concreteness, higher abstractness and same level of concreteness, higher abstractness and higher concreteness, or even some more nuances features beyond perceptual abstractness and categorical specificity (Barsalou et al., 2018; Bolognesi et al., 2020).

A few caveats are in order before concluding. Chiefly, we relied on convenience samples from English-speaking countries, and it is possible that the effects would look different in other populations. Though we used a range of methods and techniques, we were also constrained by pragmatic choices of how much participants could complete in one study session. Table 5 summarizes a range of design-based limitations. Future studies could also explore other areas where thinking both abstractly and concretely helps. One candidate concerns the idea of mental contrasting (Oettingen & Reininger, 2016), according to which it is beneficial for people to contrast the desired future with present realities. Another candidate comes from the scholarship on the folk standards of judgment (Grossmann, Eibach, et al., 2020; Meyers et al., 2021), which reveals that people often forgo the rational choice in favor of a reasonable accommodation. Notably, in many cultures (Chinese, English, Russian, Spanish, Portuguese, and Urdu), the folk standard of reasonableness appears to integrate both considerations of abstract ideals with concrete situational constraints. Systematic comparison of psychological domains for which construal switching may be beneficial as well as individual differences in meta-knowledge about these domains also opens a new direction for CLT and regulatory scope research.

**Table 5.** Overview of Study Limitations.

**Limitation**	**Implication**
Reliance on convenience samples	Effects may not generalize to less educated strata of the population
Reliance on English-speaking samples	Effects may not generalize to role of abstractness and concreteness in other languages
Reliance on North American and UK samples	Effects may not generalize to non-Western populations
Lack of examination of other mental representations	Mental representations other than abstractness and concreteness may be equally or more important for wise thought and should be examined in future research
Scenarios as fixed effects	Using a wide range of scenarios for testing intra-individual variability in mental representations (as random effects) can further strengthen ecological validity
NLP analyses in Study 1 are sensitive to the narrative response length and were performed on group vs. individual level	NLP requires a sufficient amount of text to estimate nuanced associations between abstractness and concreteness in narratives, calling for narrative-focused designs.
We relied on self-rated acts as to assess wisdom-related characteristics	Participants’ behavior may not have been fully captured due to the limits of self-insight into one’s reflective process. Observer-based methods for assessing intellectual humility, open-mindedness or perspective-taking via non-verbal behavioral cues may need to be developed and validated.

*Note*. This table provides an overview of the key limitations identified in the studies conducted and the potential implications of these limitations on the study’s findings and conclusions.

How people think about events, goals, or people can affect how well they function. Our studies suggest that thinking about both abstract and concrete features of a situation is linked to wise reflections on one’s life’s challenges. Moreover, people mentally represent abstractness and concreteness as different kinds of thinking rather than levels of one kind of thinking.

## ACKNOWLEDGMENTS

We thank Richard Eibach, Cheryl Wakslak, and Richard Lucas for feedback and suggestions and Adrienne Paynter and Mane Kara-Yakoubian for research assistance, and Allison Ledgerwood, Kentaro Fujita, Klaus Fiedler, Tal Eyal, and Oren Bornstein for their expert perspectives on core features of construal-level theory.

## AUTHOR CONTRIBUTIONS

IG: Conceptualization, Data curation, Formal analysis, Funding acquisition, Methodology, Project administration, Visualization, Writing – original draft, Writing – review & editing. JP: Conceptualization, Data curation, Funding acquisition, Methodology, Writing – review & editing. AD: Data curation, Methodology, Writing – review & editing. AR: Methodology, Writing – review & editing. RB: Conceptualization, Methodology, Writing – review & editing.

## OPEN SCIENCE PRACTICES

Pre-registrations, materials, data, and reproducible analyses are available on Open Science Framework page of the project: https://osf.io/r6huj/.

## DATA AVAILABILITY STATEMENT

Data, analysis scripts and materials are available here: https://osf.io/r6huj.

## Notes

^1^ It is possible that the *subjective* sense of relying on both abstract and concrete construal originates from the switching between “high” and “low” levels of the same *objective* dimension. The present work does not concern the question of the realism of construal processes–i.e., whether and how mental processes such as construals are rooted in objective reality. Instead, the present claims squarely target the phenomenology by examining measurement of subjective mental representations.^2^ Study 1b pre-registration treated psychological distance items as part of the SACCS. Per Study 1a expert recommendations, we treated these items as a distinct construct. Further, one abstract item (I thought about whether this situation was typical) was not included in the confirmatory Prolific sample of Study 1b.^3^ The positive association between abstractness and interpersonal closeness after a transgression might seem to contradict the theory linking abstractness with psychological distance. However, this apparent inconsistency is resolved by considering the sequence of events in our study. Participants first assessed the abstractness of their reactions during the conflict before later evaluating their current feelings of closeness to the involved individual. This sequence suggests that abstract construal in reflection on an ongoing conflict may facilitate a more amicable resolution, thereby reducing psychological distance and enhancing feelings of closeness post-conflict. This interpretation aligns with claims that psychological distance can, under certain conditions, promote resolution strategies that ultimately bring individuals closer (Kross & Ayduk, 2017, for a review).^4^ Whereas the SACCS was consistently associated with mental features of wisdom in the moderate-high effect size range, *r*_abstract_ = .51, *r*_concrete_ = .48, trait-style measure of abstractness (BIF) showed weak associations to wisdom, *r* = .10.^5^ Reliability across four items of the intellectual humility sub-scale was moderate for one of the events during the first measurement wave, *α* = .67, but very good for all other events, *Md* *α* = .82.^6^ Abstractness and concreteness on the SACCS interacted in predicting construal disbalance and switching (see Figures S13–S14 in the supplement): Participants who reported both high abstractness and concreteness showed lowest construal disbalance and greatest number of construal switches. This suggests that people who report engaging with both concrete and abstract considerations on the SACCS showed more switching between abstract and concrete strategies on a separate task.

## Supplementary Material


