# Supplementary material for: The Wise Mind Balances the Abstract and the Concrete
Source: Open Mind (Camb). 2024 Jun 28;8:826–58. doi: 10.1162/opmi_a_00149 (PMC11226238; doi:10.1162/opmi_a_00149)
Supplement: Supplementary file 1 [file opmi-08-826-s001.docx]

**Supplement**

**for**

**The wise mind balances the abstract and the concrete**

Igor Grossmann ^a^

University of Waterloo

Johanna Peetz ^b^

Carleton University

Anna Dorfman ^c^

Bar-Ilan University

Amanda Rotella ^d^

Northumbria University

Roger Buehler ^e^

Wilfrid Laurier University

^a^ Department of Psychology, University of Waterloo, 200 University Avenue West, Waterloo, ON N2L 3G1 Canada. Email: [igrossma@uwaterloo.ca](mailto:igrossma@uwaterloo.ca)

^b^ Psychology Department, Carleton University, 1125 Colonel By Drive, Ottawa, ON K1S5B6 Canada. Email: [johanna.peetz@carleton.ca](mailto:johanna.peetz@carleton.ca)

^c^ Department of Psychology, Bar-Ilan University, Ramat Gan, Israel, 590002. Email: [anna.dorfman@biu.ac.il](mailto:anna.dorfman@biu.ac.il)

^d^ Department of Psychology, Northumbria University, Sutherland Building, 2 Ellison Pl, Newcastle upon Tyne, NE1 8ST, United Kingdom. Email: [amanda.rotella@northumbria.ac.uk](mailto:amanda.rotella@northumbria.ac.uk)

^e^ Psychology Department, Wilfrid Laurier University, 75 University Avenue West, Waterloo, ON N2L 3C5 Canada. Email: [rbuehler@wlu.ca](mailto:rbuehler@wlu.ca)

**Online Supplement**

[Pilot studies 4](#_Toc162281967)

[Table S1. Pilot study Demographics across 4 samples and level of engagement (reflection length) 4](#_Toc162281968)

[Pilot Study A 5](#_Toc162281969)

[Method 5](#_Toc162281970)

[Table S2: Construal items used across pilot study pilot studies 6](#_Toc162281971)

[Principal component analyses of abstract and concrete construal items 7](#_Toc162281972)

[Table S3: Zero-order correlations of construal items in Pilot Study Sample A 8](#_Toc162281973)

[Table S4: Zero-order correlations of construal items across all 4 samples 8](#_Toc162281974)

[Factor analysis of wisdom (maximum likelihood extraction method) 8](#_Toc162281975)

[Table S5: Construal indices and wisdom in Pilot Study Sample A 10](#_Toc162281976)

[Table S6: Construal items and wisdom in Pilot Study Sample A 11](#_Toc162281977)

[Abstractness and concreteness in reflections on the past and anticipation of the future 11](#_Toc162281978)

[Pilot Study B 13](#_Toc162281979)

[Method 13](#_Toc162281980)

[Table S7: Construal indices and wisdom in Pilot Study B 15](#_Toc162281981)

[Frequency of considering pros and cons as an indirect measure of desirability and feasibility 15](#_Toc162281982)

[Pilot Study C 16](#_Toc162281983)

[Table S8: Construal indices and wisdom in Sample C 21](#_Toc162281984)

[Pilot Study D 22](#_Toc162281985)

[Method 22](#_Toc162281986)

[Table S9: Construal indices and wisdom in Sample D 24](#_Toc162281987)

[Mega-analysis of Pilot Studies 25](#_Toc162281988)

[Discussion of Pilot Study Results 28](#_Toc162281989)

[Study 1 29](#_Toc162281990)

[Study 1a Analytical steps 29](#_Toc162281991)

[Study 1b 32](#_Toc162281992)

[Table S9: EFA in Study 1a, Round 1 34](#_Toc162281993)

[Table S10: EFA in Study 1a, Round 2 35](#_Toc162281994)

[Table S11: EFA in Study 1a, Round 3 36](#_Toc162281995)

[Table S12: EFA in Study 2a, Round 4 36](#_Toc162281996)

[Table S13: EFA in Study 1a, Round 5 37](#_Toc162281997)

[Table S14: Modification indices for a 2-factor model of construal in Study 1a 39](#_Toc162281998)

[Table S15: EFA in Study 1b, Round 1 40](#_Toc162281999)

[Table S16: EFA in Study 1b, Round 2 41](#_Toc162282000)

[Table S17: Factor Loadings in Study 1b, Final Round 4 42](#_Toc162282001)

[Table S18: Parameter Estimates of the Bifactor Model in Study 1b – North America 43](#_Toc162282002)

[Table S19: Parameter Estimates of the Bifactor Model in Study 1b – UK 44](#_Toc162282003)

[Table S20: Construal and Covariates Predicting Wisdom in Study 1b 45](#_Toc162282004)

[Table S21: Sample-wise Nomological Net of Abstract and Concrete Construal in Study 1b 46](#_Toc162282005)

[Figure S6: Bifactor model of abstract and concrete construal in Study 1 47](#_Toc162282006)

[Figure S7: Effects of abstract and concrete construal on holism and wisdom in Study 1 48](#_Toc162282007)

[Study 2 49](#_Toc162282008)

[Relatability of events 49](#_Toc162282009)

[Effects in the strategy ordering task 49](#_Toc162282010)

[Effects in disbalance on different features of wisdom 49](#_Toc162282011)

[Effects in switching on different features of wisdom 49](#_Toc162282012)

[Table S22: Descriptive statistics, zero-order correlations, and measures of intra-individual and within-wave stability of key measures in Study 2. 51](#_Toc162282013)

[Figure S8: Relatability of events in Study 2 52](#_Toc162282014)

[Figure S9: Abstractness of events in Study 2 53](#_Toc162282015)

[Figure S10: Concreteness of events in Study 2 54](#_Toc162282016)

[Figure S11: Average wisdom for each event in Study 2 55](#_Toc162282017)

[Figure S12: Likelihood of selecting abstract strategies for different ranks in Study 2 56](#_Toc162282018)

[Figure S13: SACCS-based ratings of construal and construal disbalance in Study 2 57](#_Toc162282019)

[Figure S14: SACCS-based ratings of construal and construal switching in Study 2 58](#_Toc162282020)

[Supplementary References 59](#_Toc162282021)

# Pilot studies

Across four samples, we explored how abstract and concrete construal relates to mental features of wisdom across a range of situational contingencies, including different temporal foci, types of challenge, and varying degree of abstractness. In each sample, we measured construal and the extent to which people engaged in commonly agreed mental features of wisdom ^1^.

The first sample tested the boundary condition of the construal-wisdom relationship concerning temporal focus ^2,3^, exploring whether thoughts about a difficult social situation that occurred in the past versus is expected to occur in the future might influence participants' construal levels, thereby allowing us to examine subsequent variability in wisdom. The second sample aimed to replicate the observed effect on future events (a difficult social situation participants expect to occur). The third sample experienced an experimental manipulation of construal via “how” versus ‘why” instructions ^4^, to probe individual-level associations between construal and wisdom when explicitly instructed to consider abstract (focus on “why”) or concrete (focus on “how”) goals. The fourth sample shifted focus from idiosyncratic autobiographical experiences and anticipated events to standardized scenarios used in earlier construal research ^5^: Participants contemplated decision situations involving consumer choice (i.e., choosing between restaurants, phone apps, shelving units). For the sake of parsimony, we rely on (mega-)analytic estimates across four samples, as well as meta-analyses of scores from each sample.

We hypothesized that both abstract and concrete construals will independently predict wise reasoning. Indeed, our expectation is that effects of abstract and concrete construal will be additive, such that wisdom will be strongest to the extent that participants spontaneously engage in both types of construals. Table 1 presents demographics across samples.

In line with pre-registered predictions, the results from each pilot study showed that both abstract and concrete construal were both positively associated with mental features of wisdom. The effects were consistent across different features of construal, and when controlling for length of reflection. Critically, and in line with previous CLT research ^5,6^, abstractness also uniquely related to desirability concerns and concreteness to feasibility concerns, supporting the discriminant validity of the construal measures; thus, the results were unlikely to be due to a common method factor. Results for each pilot study are below.

## Table S1. Pilot study Demographics across 4 samples and level of engagement (reflection length)

| Pilot Study | A | B | C | D |  |
| --- | --- | --- | --- | --- | --- |
| Recruited | 354 | 116 | 237 | 313 |  |
| Final sample | 330 | 97 | 218 | 270 |  |
| Age *M* (*SD*) | 36.87 (10.87) | 35.25 (11.16) | 36.50 (11.98) | 36.58 (11.68) |  |
| % Female | 45.1 | 45.4 | 55.9 | 40.7 |  |
| Reflection probe words *M* (*SD*) | 54.19 (37.86) | 57.58 (29.98) | 65.77 (49.95) | 20.56 (11.68) |  |

*Note.* Ethnicity information was not recorded due to technical errors.

## Pilot Study A

### Method

The data collection procedure, scale aggregation plan and the hypotheses were preregistered (<https://osf.io/9x4wq>). We hypothesized that construal level dimensions would be independent dimensions, and that being high on both abstract and concrete thought would be linked with wise reasoning about interpersonal situations. We also preregistered hypotheses comparing the past vs future event conditions, which are reported separately below; conditions are aggregated in main analyses as no condition differences emerged. Note that this and other pilot study preregistrations were uploaded prior to data collection and timestamped on OSF but were not frozen via the preregistration function in OSF.

*Participants***.** We aimed to recruit 334 participants, based on a priori power analysis: A small regression interaction effect, *f* = .20 (Cohen, 1992), would be detectable with 80% power in a sample of 304. We oversampled to account for exclusions. We recruited 354 U.S. MTurk workers (compensated with $0.50). Of the initial sample, 14 were excluded and replaced because they ended the study early (i.e., before completing the wise reasoning scale), and 10 participants were excluded after data collection had ended because they did not describe an event (a preregistered exclusion criterium). The final sample included 344 participants who completed at least some of the construal items, and 330 participants who completed both construal- and wisdom-related items (*M*_age_ = 36.87, *SD* = 10.87; 45.1% female).

*Procedure.* Participants were instructed to describe a “difficult social situation” in their life (Brienza et al., 2018) that they anticipated occurring in the future (future condition, *n* = 171) or one that happened in the past (past condition, *n* = 159). Differential attrition across conditions is expected, as more people reported no recalling versus anticipating experiencing a difficult social situation. Reported events included conflicts with coworkers “I'm thinking of doing an annual review with one specific person who reports to me. (…) I'm not sure how it will go, he'll either recognize the problem or get defensive” and family “I might have to ask my mother for another loan soon, in order to cover rent for the month.” On average, participants wrote 53.25 words (*SD* = 37.82). After thinking about and writing down thoughts about the event, participants indicated the extent to which they engaged in abstract and concrete construals, as well as their wisdom.

*Construal items.* To assess abstract construal, participants rated the extent to which they focused on the goal (*why* to do things; Fujita et al., 2006); considered the event as an instance of a broader category (outside view) (Kahneman & Lovallo, 1993; Ledgerwood et al., 2010); and pictured the event from a third-person perspective (Libby & Eibach, 2011; Shaeffer, Libby, & Eibach, 2015). To assess concrete construal, participants rated the extend to which they focused on the process (*how* to do things), considered the event as unique (inside view), and pictured the event from a first-person perspective. These items captured three features of construal: action representation, perspective, and mental model. Participants reported the extent of these thoughts while recalling or imagining the difficult social situation. To assess *abstract construal*, participants reported how much they had thought of reasons for the event and about what the other person wanted (i.e., the “why” of the event; 2 items, *r* = .54, *p* < .001), how much they had thought of the event from a third-person perspective (1 item), and how much they regard the situation as one of many similar ones (1 item; inter-item correlation across all items: .10 < *r ≤* .53). To assess *concrete construal*, participants reported how much they had thought of what they and the other person were doing and saying and what they and the other person looked like (i.e., the “how” of the event; 2 items, *r* = .32, *p* < .001), how much they had thought of the event from a first-person perspective (1-item), and how much they regard the situation as unique (1 item; inter-item correlation across all items: .11 < *r ≤* .32). Participants rated their construal on scales from *Not at all* (1) to *Very much* (7). See Table S2 for verbatim items in this and following samples.

## Table S2: Construal items used across pilot study pilot studies

| Sample | Domain | Abstract | Concrete |
| --- | --- | --- | --- |
| A | Action representation (why - goal / how - process) | …think about what you and the other person wanted  …think about the reasons you and the other person were behaving this way | …think about the details of what you and the other person looked liked  …think about what you and the other person were saying and doing |
|  | Perspective (third-person /first-person) | …see the situation as an observer would (i.e., from a third person perspective) | …see the situation through your own eyes (i.e., from a first-person perspective) |
|  | Mental model (broad outside view / unique inside view) | …think about the situation as one of many similar experiences | …think about the situation as one-of-a kind, a unique experience |
| B | Action representation (why - goal / how - process) | I think about why this event would occur (e.g., reasons or causes) | I think about how the event will unfold (e.g., concrete actions) |
|  | Perspective (third-person /first-person) | I see the situation as an observer would (i.e., from a third person perspective) | I see the situation through my own eyes (i.e., from a first-person perspective) |
|  | Mental model (broad outside view / unique inside view) | I think about the situation as one of many similar experiences | I think about the situation as one-of-a-kind, a unique experience |
| C | Action representation (why - goal / how - process) | I think about what my reasons for choosing one or the other would be | I think about how the choice might unfold, step-by-step |
|  | Perspective (third-person /first-person) | I imagine myself choosing as an observer would (i.e., from a third person perspective | I imagine myself choosing through my own eyes (i.e., from a first-person perspective) |
|  | Mental model (broad outside view / unique inside view) | I think about the choice as one of many similar choices | I think about the choice as one-of-a kind, a unique choice |
| D | Action representation (why - goal / how - process) | I think about why this event would occur (e.g., reasons or causes) | I think about how the event will unfold (e.g., concrete actions) |
|  | Perspective (third-person /first-person) | I see the situation as an observer would (i.e., from a third person perspective) | I see the situation through my own eyes (i.e., from a first-person perspective) |
|  | Mental model (broad outside view / unique inside view) | I think about the situation as one of many similar experiences | I think about the situation as one-of-a-kind, a unique experience |

*Wisdom.* We used the Situated Wisdom Scale (SWiS; Brienza et al., 2018), which instructed people to consider a specific issue, mentally visualize and reflect on it, and then report on core features of wisdom in their reflections. Participants rated their thoughts about the situation using a 21-item scale (Brienza et al., 2018). This scale asked participants to rate the extent to which they engaged in thought processes characteristic of wisdom (1 = *not at all* to 5 = *very much*). Items assessed the following features: considering other perspectives (“other perspectives”, e.g., I put myself in the other person's shoes, 4 items, α = .88), intellectual humility - recognizing the limits of one’s knowledge (“intellectual humility”, e.g., I behaved as if there may be information to which I did not have access, 4 items, α = .83), sensitivity to the possibility of multiple ways an issue might unfold (“multiple ways”, e.g., I looked for different solutions as the situation evolved, 4 items, α = .78); search for compromise or integration of different opinions (“search for compromise”, e.g., I tried my best to find a way to accommodate both of us, 5 items, α = .81); taking a self-transcendent viewpoint (“self-transcendence”, e.g., I tried to see the conflict from the point of view of an uninvolved person, 4 items, α = .89). Because self-transcendent viewpoint shares conceptual overlap with one of the abstract construal items (third-person perspective), we treated this facet separately. The remaining subscales were also averaged into an overall wisdom index (α = .83), following the method outlined in Brienza et al. (2018).

*Data reduction*. As outlined in the pre-registered plan ([https://osf.io/gx9cs](https://osf.io/gx9cs/?view_only=c5a867dbaab34833b438cab3b8f8d310)), we performed main analyses on the aggregate average metrics of abstract and concrete thought across respective four items. This approach also allowed us to compare scores across Studies 1a-1d. Inter-item correlations for each construal were positive, but modest (Table S4). For data reduction purposes, we examined principal component and axes analyses, which indicated that either a two-(based on scree plot) or three-component solution (based on Eigenvalues > 1) provide the best component-to-explained-variance ratio. However, the component loadings were hard to interpret. For the sake of parsimony in our main analyses we followed the pre-registered plan and focused on the averaged indices across items a priori forming abstract and concrete construal dimensions (as defined in the pre-registration) without making claims about underlying latent structure.

### Principal component analyses of abstract and concrete construal items

First, we explored data reduction solutions on all participants who completed construal items (*N* = 344). The screeplot indicated a 2-component solution representing the most meaningful fit of the data, whereas eigenvalue rule of thumb (> 1) suggested a 3-component solution. Hence, we examined both 2 and 3 component solutions, using oblimin rotations.

We used the oblimin rotation because we wanted to explore whether the resulting factors were inter-related (either negatively, as CLT suggests; or positively, as we hypothesized). As results below indicate, a 2-component solution yield most items loading on the first component, with two abstract items (third-person perspective and commonness-oriented mental model) loading on the second item. In contrast, a 3-component solution yield one component dealing with Commonness and Uniqueness (reverse-loaded) set of items, another component dealing with the visual imagery of the situation (think about the details of what you and the other person looked liked; see the situation as an observer would (i.e., from a third person perspective)), and a third component with positive loadings of all other items. Results from a parallel set of exploratory principal axis analyses were comparable. Overall, it appears that the measures previously used to characterize individual variation in abstract and concrete construal do not provide evidence of a single underlying latent factor, with inverse loadings of abstract and concrete items.

**2 component solution**

| *Pattern Matrix^a^* | | |
| --- | --- | --- |
|  | Component | |
|  | 1 | 2 |
| 1C. HOW-A think about the details of what you and the other person looked liked | **.555** | .213 |
| 2C. HOW-B think about what you and the other person were saying and doing | **.744** | -.081 |
| 3C. FIRST-PERSON see the situation through your own eyes (i.e., from a first person perspective) | **.600** | -.076 |
| 4C. UNIQUE think about the situation as one-of-a kind, a unique experience | **.494** | -.555 |
| 1A. WHY-A think about what you and the other person wanted | **.730** | .111 |
| 2A. WHY-B think about the reasons you and the other person were behaving this way | **.710** | .108 |
| 3A. THIRD-PERSON see the situation as an observer would (i.e., from a third person perspective) | .163 | **.451** |
| 4A. COMMON think about the situation as one of many similar experiences | .102 | **.885** |
| Extraction Method: Principal Component Analysis. Oblimin with Kaiser Normalization. | | |

**3 component solution**

| *Pattern Matrix^a^* | | | |  |
| --- | --- | --- | --- | --- |
|  | Component | | | |
|  | 1 | 2 | 3 | |
| 1C. HOW-A think about the details of what you and the other person looked liked | .390 | -.046 | .484 | |
| 2C. HOW-B think about what you and the other person were saying and doing | .778 | -.081 | -.070 | |
| 3C. FIRST-PERSON see the situation through your own eyes | .758 | .065 | -.315 | |
| 4C. UNIQUE think about the situation as one-of-a kind, a unique experience | .173 | -.819 | .305 | |
| 1A. WHY-A think about what you and the other person wanted | .734 | .041 | .106 | |
| 2A. WHY-B think about the reasons you and the other person were behaving this way | .651 | -.027 | .223 | |
| 3A. THIRD-PERSON see the situation as an observer would | -.139 | .026 | .871 | |
| 4A. COMMON think about the situation as one of many similar experiences | .186 | .788 | .390 | |
| Extraction Method: Principal Component Analysis. Oblimin with Kaiser Normalization. | | | |  |

In this sample (Table S3) and also across pilot samples (Table S4), we also observed little evidence of greater unity of items within either abstract or concrete construal cluster: As Tables S3-S4 show, some of the cross-correlations were comparable if not higher than the correlation within a respective cluster. For the sake of parsimony and to ensure comparability across samples, we continued with using average scores across respective items as “indices” of abstract and concrete construal, without any claim for a possible underlying latent factor structure, simultaneously presenting item-wise analyses for each of the respective components. To ensure comparability to Studies 1b-1d, we first averaged respective why / how items and then averaged the three indices into markers of abstract and concrete construal.

## Table S3: Zero-order correlations of construal items in Pilot Study Sample A

|  | Abstract 4 | Abstract 3 | Abstract 2 | Abstract 1 | Concrete 4 | Concrete 3 | Concrete 2 |
| --- | --- | --- | --- | --- | --- | --- | --- |
| Concrete 1 | .24*** | .19** | .28*** | .29*** | .24*** | .18* | .32*** |
| Concrete 2 | .08 | .04 | .38*** | .45*** | .17* | .43*** |  |
| Concrete 3 | .14 | -.09 | .27*** | .33*** | .11 |  |  |
| Concrete 4 | -.30*** | .17* | .19** | .16* |  |  |  |
| Abstract 1 | .19** | .09 | .54*** |  |  |  |  |
| Abstract 2 | .18* | .15* |  |  |  |  |  |
| Abstract 3 | .26*** |  |  |  |  |  |  |

*Notes. p*-value adjustment method: Holm (1979). ***Correlation is significant at the .001 level (2-tailed). **Correlation is significant at the .01 level (2-tailed). *Correlation is significant at the .05 level (2-tailed).

## Table S4: Zero-order correlations of construal items across all 4 samples

| Sample |  | 1C How | 2C  1st-person | 3C  Unique | 1A  Why | 2A  3rd-person | 3A  Common |
| --- | --- | --- | --- | --- | --- | --- | --- |
| A^a^ | 1C | -- | **.344^***^** | **.263^***^** | .454^***^ | .164^***^ | .219^***^ |
|  | 2C |  | **--** | **.110^*^** | .339^***^ | -.082 | .135^*^ |
|  | 3C |  |  | -- | .199^***^ | .176^***^ | -.299^***^ |
|  | 1A |  |  |  | -- | **.144^**^** | **.210^***^** |
|  | 2A |  |  |  |  | **--** | **.260^***^** |
| B | 1C | -- | **.603^***^** | **.122** | .297^**^ | .012 | .125 |
|  | 2C |  | **--** | **.191** | .293^**^ | -.242^*^ | .003 |
|  | 3C |  |  | -- | .342^***^ | .164 | -.467^***^ |
|  | 1A |  |  |  | -- | **.193** | **.067** |
|  | 2A |  |  |  |  | **--** | **.276^**^** |
| C | 1C | -- | **.381^***^** | **.219^*^** | .243^**^ | .134 | .001 |
|  | 2C |  | **--** | **.116** | .361^***^ | -.243^**^ | .093 |
|  | 3C |  |  | -- | .089 | .149 | -.458**^***^** |
|  | 1A |  |  |  | -- | **.143** | **.201^*^** |
|  | 2A |  |  |  |  | **--** | **.266^***^** |
| D | 1C | -- | **.274^***^** | **.315^***^** | .172^**^ | .293^***^ | .272^***^ |
|  | 2C |  | **--** | **.197^***^** | .325^***^ | -.113 | .178^**^ |
|  | 3C |  |  | -- | .179^**^ | .260^***^ | -.137^*^ |
|  | 1A |  |  |  | -- | **-.071** | **.195^***^** |
|  | 2A |  |  |  |  | **--** | **.209^***^** |

*Notes:* a: *How* and *Why* represent averages across respective two items. *** *p* < .001 ** *p* < .01 * *p* < .05 (all two-tailed).

### Factor analysis of wisdom (maximum likelihood extraction method)

To generate an aggregate index of wisdom, we followed the Brienza et al. procedure ^7^: First, we generated facet scores by averaging respective items. Subsequently, we averaged scores for four facets (intellectual humility, multiple ways, others’ perspectives, search for compromise) into the wisdom index. This way, each facet was weighted equally in the resulting scale. To avoid conceptual overlap with abstract construal, we examined self-transcendence separately. Results indicated a 5-factor solution (Eigenvalues > 1), consistent with prior research ^7^. Examination of the factor loadings was mostly consistent with prior research with one minor exception: Two of the items concerning open-minded view of change (5. Looked for different solutions as the situation evolved; 6. Considered alternative solutions as the situation evolved) loaded on search for compromise and conflict resolution factors. This pattern is not unprecedented and was also observed in some samples by Grossmann, Oakes, and Santos ^8^. Notably, factor correlation matrix indicates a consistent set of positive correlations between each of the factors in a medium-high range, .231 <*r*s < .556.

| **5 component factor solution of wise reasoning items** | | | | | |
| --- | --- | --- | --- | --- | --- |
|  | Factor | | | | |
|  | 1 | 2 | 3 | 4 | 5 |
| 1. Put myself in the other person's shoes | .021 | **.846** | .071 | -.045 | -.061 |
| 2. Tried to communicate with the other person what we might have in common | .009 | **.560** | .056 | .015 | .207 |
| 3. Made an effort to take the other person's perspective | .032 | **.871** | .015 | -.054 | .008 |
| 4. Took time to get the other person's opinions on the matter before coming to a conclusion | .064 | **.596** | -.111 | -.180 | .156 |
| 5. Looked for different solutions as the situation evolved | .026 | .021 | -.053 | -.025 | **.772** |
| 6. Considered alternative solutions as the situation evolved | .004 | -.036 | -.074 | -.058 | **.805** |
| 7. Believed the situation could lead to a number of different outcomes | **1.038** | .002 | -.022 | .032 | -.054 |
| 8. Thought the situation could unfold in many different ways | **.707** | .033 | .033 | -.002 | .077 |
| 9. Double-checked whether my opinion on the situation might be incorrect | .017 | -.029 | -.006 | **-.899** | -.006 |
| 10. Double-checked whether the other person's opinions might be correct | -.049 | .028 | -.007 | **-.832** | .008 |
| 11. Looked for any extraordinary circumstances before forming my opinion | .017 | .082 | .127 | **-.522** | .086 |
| 12. Behaved as if there may be some information to which I did not have access | .124 | .235 | .103 | **-.353** | .004 |
| 13. Tried my best to find a way to accommodate both of us | -.036 | .219 | .019 | .080 | **.630** |
| 14. Though it may not have been possible, I searched for a solution that could result in both of us being satisfied | -.042 | .164 | .053 | .030 | **.678** |
| 15. Considered first whether a compromise was possible in resolving the situation | -.002 | .115 | .131 | -.048 | **.544** |
| 16. Viewed it as very important that we resolve the situation | .103 | -.063 | -.028 | -.058 | **.462** |
| 17. Tried to anticipate how the conflict might be resolved | .065 | -.098 | .191 | -.044 | **.546** |
| 18. Wondered what I would think if I was somebody else watching the situation | -.005 | -.015 | **.809** | .036 | .000 |
| 19. Tried to see the conflict from the point of view of an uninvolved person | .046 | .109 | **.740** | -.051 | -.022 |
| 20. Asked myself what other people might think or feel if they were watching the conflict | .033 | -.006 | **.902** | .019 | .009 |
| 21. Thought about whether an outside person might have a different opinion from mine about the situation | -.031 | -.044 | **.761** | -.108 | .032 |
| Extraction Method: Maximum Likelihood. Oblimin with Kaiser Normalization. | | | | | |

Analytical Procedure. Following our pre-registration, we relied on correlational and linear regression analyses to explore how indices of both abstract and concrete construals relate to each other and to wisdom. Further, we probed these associations as a function of reflecting on past versus future events. As outlined in the pre-registered plan, we performed main analyses on the aggregate average metrics of abstract and concrete construal. In this sample, we averaged two items of “why”/ “how” items respectively, prior to averaging scores for goal/process-oriented action representations, third/first-person perspectives, and broad/unique mental models. This procedure was not preregistered but allows us to compare scores across all four samples. Table S5 presents links between construal indices and the overall wise reasoning score as well as each wise reasoning facet. Table S6 reports item-wise analyses for individual construal items and the overall wise reasoning score as well as each individual wise reasoning facet.

## Table S5: Construal indices and wisdom in Pilot Study Sample A

| *DV* | *Predictor* | *Est.* | *S.E.* | *t* | *p* |
| --- | --- | --- | --- | --- | --- |
| Wisdom index  *R*^2^ = .160 | (Intercept) | 3.288 | 0.043 | 75.791 | < .001 |
|  | **abstract** | **0.184** | **0.037** | **4.924** | **< .001** |
|  | **concrete** | **0.163** | **0.038** | **4.293** | **< .001** |
|  | abstract*concrete | 0.038 | 0.025 | 1.507 | .133 |
| Other perspectives *R*^2^ = .115 | (Intercept) | 3.153 | 0.061 | 51.826 | < .001 |
|  | **abstract** | **0.271** | **0.052** | **5.159** | **< .001** |
|  | *concrete* | *0.091* | *0.059* | *1.722* | *.086* |
|  | abstract*concrete | 0.050 | 0.035 | 1.421 | .156 |
| Intellectual humility  *R*^2^ = .119 | (Intercept) | 2.972 | 0.058 | 51.026 | < .001 |
|  | **abstract** | **0.199** | **0.050** | **3.958** | **< .001** |
|  | **concrete** | **0.179** | **0.051** | **3.527** | **< .001** |
|  | *abstract*concrete* | *0.065* | *0.033* | *1.930* | *.054* |
| Multiple ways  *R*^2^ = .106 | (Intercept) | 3.447 | 0.051 | 67.839 | < .001 |
|  | **abstract** | **0.103** | **0.044** | **2.358** | **.019** |
|  | **concrete** | **0.210** | **0.044** | **4.736** | **< .001** |
|  | abstract*concrete | 0.043 | 0.029 | 1.466 | .144 |
| Search for a compromise *R*^2^= .110 | (Intercept) | 3.581 | 0.050 | 71.717 | < .001 |
|  | **abstract** | **0.164** | **0.043** | **3.811** | **< .001** |
|  | **concrete** | **0.170** | **0.044** | **3.890** | **< .001** |
|  | abstract*concrete | -0.007 | 0.029 | -0.236 | .814 |
| Self-transcendence  *R*^2^ = .113 | (Intercept) | 3.028 | 0.062 | 48.763 | < .001 |
|  | **abstract** | **0.262** | **0.054** | **4.888** | **< .001** |
|  | **concrete** | **0.141** | **0.054** | **2.603** | **.010** |
|  | abstract*concrete | 0.016 | 0.036 | 0.455 | .649 |

*Notes*. All continuous predictors are mean-centered. Effects significant at *p* < .05 are in bold. Effects significant at *p* < .10 are in italics.

## Table S6: Construal items and wisdom in Pilot Study Sample A

*Multiple regressions with construal items simultaneously predicting mental features of wisdom (total and each facet), as well as self-transcendence in Pilot Study A.*

|  | Estimate | *Est.* | *S.E.* | *\|t\|* | *p* |
| --- | --- | --- | --- | --- | --- |
| Wisdom Index  *R*^2^ = .220 | (Intercept) | 1.703 | 0.239 | 7.128 | < .001 |
|  | think about the details of what you and the other person looked liked (concrete 1) | 0.022 | 0.024 | 0.898 | .370 |
|  | think about what you and the other person were saying and doing (concrete 2) | 0.007 | 0.037 | 0.195 | .846 |
|  | see the situation through your own eyes (i.e., from a first person perspective) (concrete 3) | 0.005 | 0.031 | 0.174 | .862 |
|  | **think about the situation as one-of-a kind, a unique experience (concrete 4)** | **0.08** | **0.025** | **3.23** | **.001** |
|  | **think about what you and the other person wanted (abstract 1)** | **0.112** | **0.032** | **3.473** | **.001** |
|  | think about the reasons you and the other person were behaving this way (abstract 2) | -0.012 | 0.033 | -0.375 | .708 |
|  | **see the situation as an observer would (i.e., from a third person perspective) (abstract 3)** | **0.073** | **0.025** | **2.952** | **.003** |
|  | **think about the situation as one of many similar experiences (abstract 4)** | **0.065** | **0.027** | **2.407** | **.017** |
| Other perspectives  *R*^2^ = .135 | (Intercept) | 1.52 | 0.338 | 4.492 | < .001 |
|  | think about the details of what you and the other person looked liked (concrete 1) | 0.033 | 0.034 | 0.974 | .331 |
|  | think about what you and the other person were saying and doing (concrete 2) | 0.044 | 0.053 | 0.835 | .404 |
|  | see the situation through your own eyes (i.e., from a first person perspective) (concrete 3) | -0.036 | 0.044 | -0.812 | .417 |
|  | think about the situation as one-of-a kind, a unique experience (concrete 4) | 0.028 | 0.035 | 0.805 | .422 |
|  | **think about what you and the other person wanted (abstract 1)** | **0.114** | **0.046** | **2.483** | **.014** |
|  | think about the reasons you and the other person were behaving this way (abstract 2) | -0.005 | 0.046 | 0.100 | .92 |
|  | **see the situation as an observer would (i.e., from a third person perspective) (abstract 3)** | **0.115** | **0.035** | **3.279** | **.001** |
|  | **think about the situation as one of many similar experiences (abstract 4)** | **0.076** | **0.038** | **2.002** | **.046** |
| Intellectual humility  *R*^2^ = .189 | (Intercept) | 1.575 | 0.314 | 5.012 | < .001 |
|  | **think about the details of what you and the other person looked liked (concrete 1)** | **0.056** | **0.032** | **1.766** | **.078** |
|  | think about what you and the other person were saying and doing (concrete 2) | -0.073 | 0.049 | 1.485 | .138 |
|  | see the situation through your own eyes (i.e., from a first person perspective) (concrete 3) | -0.025 | 0.041 | 0.624 | .533 |
|  | **think about the situation as one-of-a kind, a unique experience (concrete 4)** | **0.156** | **0.032** | **4.821** | **< .001** |
|  | **think about what you and the other person wanted (abstract 1)** | **0.100** | **0.043** | **2.343** | **.020** |
|  | think about the reasons you and the other person were behaving this way (abstract 2) | -0.052 | 0.043 | 1.216 | .225 |
|  | **see the situation as an observer would (i.e., from a third person perspective) (abstract 3)** | **0.073** | **0.033** | **2.246** | **.025** |
|  | **think about the situation as one of many similar experiences (abstract 4)** | **0.127** | **0.035** | **3.583** | **< .001** |
| Multiple ways  *R*^2^ = .133 | (Intercept) | 1.926 | 0.282 | 6.839 | < .001 |
|  | think about the details of what you and the other person looked liked (concrete 1) | 0.003 | 0.028 | 0.112 | .911 |
|  | think about what you and the other person were saying and doing (concrete 2) | 0.008 | 0.044 | 0.170 | .865 |
|  | see the situation through your own eyes (i.e., from a first person perspective) (concrete 3) | 0.027 | 0.036 | 0.748 | .455 |
|  | **think about the situation as one-of-a kind, a unique experience (concrete 4)** | **0.100** | **0.029** | **3.455** | **.001** |
|  | **think about what you and the other person wanted (abstract 1)** | **0.123** | **0.038** | **3.231** | **.001** |
|  | think about the reasons you and the other person were behaving this way (abstract 2) | -0.011 | 0.039 | 0.295 | .768 |
|  | see the situation as an observer would (i.e., from a third person perspective) (abstract 3) | 0.031 | 0.029 | 1.048 | .296 |
|  | think about the situation as one of many similar experiences (abstract 4) | 0.041 | 0.032 | 1.293 | .197 |
| Search for a compromise  *R*^2^ = .147 | (Intercept) | 1.793 | 0.275 | 6.525 | < .001 |
|  | think about the details of what you and the other person looked liked (concrete 1) | -0.006 | 0.028 | 0.212 | .833 |
|  | think about what you and the other person were saying and doing (concrete 2) | 0.050 | 0.043 | 1.173 | .242 |
|  | see the situation through your own eyes (i.e., from a first person perspective) (concrete 3) | 0.055 | 0.036 | 1.553 | .121 |
|  | think about the situation as one-of-a kind, a unique experience (concrete 4) | 0.034 | 0.028 | 1.192 | .234 |
|  | **think about what you and the other person wanted (abstract 1)** | **0.113** | **0.037** | **3.033** | **.003** |
|  | think about the reasons you and the other person were behaving this way (abstract 2) | 0.019 | 0.038 | 0.513 | .608 |
|  | **see the situation as an observer would (i.e., from a third person perspective) (abstract 3)** | **0.074** | **0.028** | **2.588** | **.01** |
|  | think about the situation as one of many similar experiences (abstract 4) | 0.015 | 0.031 | 0.483 | .629 |
| Self-transcendence  *R*^2^ = .188 | (Intercept) | 1.443 | 0.335 | 4.312 | < .001 |
|  | **think about the details of what you and the other person looked liked (concrete 1)** | **0.061** | **0.034** | **1.813** | **.071** |
|  | think about what you and the other person were saying and doing (concrete 2) | 0.046 | 0.052 | 0.868 | .386 |
|  | see the situation through your own eyes (i.e., from a first person perspective) (concrete 3) | -0.050 | 0.043 | 1.163 | .246 |
|  | **think about the situation as one-of-a kind, a unique experience (concrete 4)** | **0.135** | **0.035** | **3.918** | **< .001** |
|  | think about what you and the other person wanted (abstract 1) | -0.024 | 0.045 | 0.523 | .601 |
|  | think about the reasons you and the other person were behaving this way (abstract 2) | -0.031 | 0.046 | 0.667 | .505 |
|  | **see the situation as an observer would (i.e., from a third person perspective) (abstract 3)** | **0.129** | **0.035** | **3.719** | **< .001** |
|  | **think about the situation as one of many similar experiences (abstract 4)** | **0.132** | **0.038** | **3.505** | **.001** |

### Abstractness and concreteness in reflections on the past and anticipation of the future

Examining the final sample of participants who completed both construal and wisdom items, we observed significantly more concrete (*M* = 5.05, *SD* = 1.17) than abstract (*M* = 4.40, *SD* = 1.20) construal thoughts, *t*(329) = 8.05, *p* < .001. Abstract and concrete construal were significantly positively associated with each other, *r* = .24, 95%*CI* [.13, .34], *t*(330) = 4.42, *p*  < .001. This association was statistically comparable when participants considered a past situation, *r* = .24, 95%*CI* [.09, .38], *t*(159) = 3.12, *p*  < .001, or anticipated a difficult social situation in the future, *r* = .31, 95%*CI* [.16, .44], *t*(171) = 4.20, *p*  < .001. Table S3 shows that the size of association between the cluster of items concerning abstractness and concreteness, respectively, were not higher than the cross-cluster associations. Indeed, the second highest correlation was between a pair of items representing concrete construal “thinking about what you and the other person were saying and doing” and abstract construal “thinking about what you and the other person wanted,” *r* = .45. Only one pair of cross-cluster correlations was significantly negative, and it concerned concrete thinking “about the situation as one-of-a kind, a unique experience” and abstract thinking “about the situation as one of many similar experiences,” *r* = - .30. All other cross-cluster item correlations were either positive or close to zero. Together, initial analyses of self-reported construal show little support for a unidimensional nature of the instrument. Instead, they suggest that in reflections on personally relevant issue abstract and concrete construal are either independent or co-occurring. Notably, the association between self-reported abstract and concrete construal is unlikely to be a side-product of greater deliberation: magnitude of abstract-concrete construal association remains identical when controlling for reflection response length, *r* = .26, *p* < .001.

## Pilot Study B

### Method

In the next sample we aimed to replicate associations of construal level found in Pilot Study A, targeting future events. We again assessed individual differences in abstract and concrete mental representations about an upcoming difficult social interaction via self-report, and then assessed wisdom about this event. We assessed construal level similarly to Pilot Study A, asking about the extent to which participants considered the event from a first or third-person perspective, and the extent to which they considered its unique or general features. Instead of asking about details of the process and goals associated with the event, we now asked directly how much they had thought about the *how* or the *why* of the event – the central features of concrete and abstract construal ^4^.

The data collection procedure, scale aggregation, and hypotheses were preregistered and are available on OSF ([https://osf.io/x59yh](https://osf.io/x59yh?view_only=11e5256aadf1463dbdcfedb04dbd2de6)). We hypothesized that construal level dimensions would be independent dimensions, and that being high on both abstract and concrete thought would be linked with wise reasoning about interpersonal situations.

*Participants*. We aimed to recruit 100 participants, based on a priori power analysis (a small regression effect, *f* = .10, would be detectable with 80% power in a sample of 80). We oversampled to account for exclusions. We recruited 116 U.S. MTurk workers (compensated with $0.50). Of these, we excluded 15 participants because they did not complete the wisdom scale and four participants because they did not nominate an event. The final sample included 97 participants (*M*_age_ = 35.25, *SD* = 11.16; 45.4% female).

*Procedure and Materials*. As in Sample A, participants described an event they expected to happen in the future. Next, as our measure of *abstract construal*, participants rated how much (1 = *not at all* to 7 = *very much*) they had thought of *why* this event would occur, had imagined it from a third person perspective, and had thought of it as one of many similar experiences. These three items were averaged into an index of abstract thought (inter-item .07 < *r*s ≤ .23). As our measure of *concrete construal*, participants rated how much (1 = *not at all* to 7 = *very much*) they had thought of *how* the event would unfold, had imagined it from a first-person perspective, and had thought about the situation as a one-of-a-kind, unique experience. These three items were averaged into an index of concrete thought (inter-item .12 < *r*s ≤ .60). See Table S2 for verbatim items. As in Sample A, items within each construal group were significantly positively correlated, with one exception: thinking about why the event would occur was unrelated to thinking of the situation as one of many similar experiences, *r* = .067.

Wisdom was assessed with the Brienza et al. wise reasoning index. Examination of the factor loadings in the confirmatory analysis in Sample B yield a pattern that was largely consistent with prior research with a few minor exceptions: One of the perspective-taking items also cross-loaded on the search for compromise/conflict resolution factor (4. Take time to get the other person's opinions on the matter before coming to a conclusion), whereas another item loaded on the intellectual humility factor (2. Try to communicate with the other person what we might have in common). Further, two of the items concerning multiple ways to think about change (7. Believe the situation could lead to a number of different outcomes; 8. Think the situation could unfold in many different ways) loaded on search for compromise and conflict resolution factor. This minor deviation may be due to stability of factor loadings when sample sizes are small, as in the present Pilot Study B. Notably, factor correlation matrix shows a consistent set of positive correlations between each of the sub-factors in a medium-high range, .255 < *r*s < .692.

**5 component factor solution of wise reasoning items**

|  | Factor | | | | |
| --- | --- | --- | --- | --- | --- |
|  | 1 | 2 | 3 | 4 | 5 |
| 1. Put myself in the other person's shoes | .158 | .100 | -.092 | **-.557** | -.175 |
| 2. Try to communicate with the other person what we might have in common | .251 | .027 | -.057 | -.139 | **-.459** |
| 3. Make an effort to take the other person's perspective | .178 | .159 | -.265 | **-.666** | -.026 |
| 4. Take time to get the other person's opinions on the matter before coming to a conclusion | .**353** | .019 | -.352 | -.275 | -.123 |
| 5. Look for different solutions as the situation evolved | .159 | -.036 | **-.734** | -.024 | -.104 |
| 6. Consider alternative solutions as the situation evolved | -.060 | -.022 | **-.981** | -.130 | -.037 |
| 7. Believe the situation could lead to a number of different outcomes | **.487** | .236 | -.263 | .172 | -.021 |
| 8. Think the situation could unfold in many different ways | **.351** | .116 | -.363 | .083 | .074 |
| 9. Double-check whether my opinion on the situation might be incorrect | -.089 | .085 | .081 | -.058 | **-.889** |
| 10. Double-check whether the other person's opinions might be correct | -.032 | -.056 | -.099 | -.033 | **-.867** |
| 11. Look for any extraordinary circumstances before forming my opinion | .282 | .173 | -.061 | -.038 | **-.399** |
| 12. Behave as if there may be some information to which I did not have access | .172 | .059 | -.043 | .143 | **-.369** |
| 13. Try my best to find a way to accommodate both of us | **.617** | -.015 | -.121 | -.127 | -.167 |
| 14. Though it may not be possible, I would search for a solution that could result in both of us being satisfied | **.719** | -.021 | -.108 | .049 | -.198 |
| 15. Consider first whether a compromise was possible in resolving the situation | **.740** | .036 | -.138 | -.005 | -.047 |
| 16. View it as very important that we resolve the situation | **.599** | -.006 | .019 | -.202 | -.054 |
| 17. Try to anticipate how the conflict might be resolved | **.551** | .027 | -.114 | -.240 | -.041 |
| 18. Wonder what I would think if I was somebody else watching the situation | -.165 | **.718** | -.167 | .061 | -.110 |
| 19. Try to see the conflict from the point of view of an uninvolved person | .060 | **.795** | .049 | -.115 | -.058 |
| 20. Ask myself what other people might think or feel if they were watching the conflict | -.084 | **.910** | .091 | -.134 | .048 |
| 21. Think about whether an outside person might have a different opinion from mine about the situation | .241 | **.827** | .036 | .153 | -.010 |

Results. Following pre-registered analytical plans, we relied on correlational and linear regression analyses to explore how indices of abstract and concrete construal indices relate to each other and to wise reasoning. Table S7 presents links between construal indices and the overall wise reasoning score as well as each wise reasoning facet.

## Table S7: Construal indices and wisdom in Pilot Study B

| *DV* | *Predictor* | *Est.* | *S.E.* | *t* | *p* |
| --- | --- | --- | --- | --- | --- |
| Wisdom Index  *R*^2^ = .113 | (Intercept) | 3.598 | 0.091 | 39.422 | < .001 |
|  | **abstract** | **0.119** | **0.068** | **1.741** | **.085** |
|  | **concrete** | **0.230** | **0.081** | **2.822** | **.006** |
|  | abstract*concrete | 0.031 | 0.047 | 0.665 | .508 |
| Other perspectives *R*^2^ = .099 | (Intercept) | 3.516 | 0.104 | 33.833 | < .001 |
|  | abstract | 0.134 | 0.078 | 1.724 | .088 |
|  | **concrete** | **0.238** | **0.093** | **2.565** | **.012** |
|  | abstract*concrete | 0.033 | 0.053 | 0.616 | .540 |
| Intellectual humility  *R*^2^ =.090 | (Intercept) | 3.263 | 0.11 | 29.567 | < .001 |
|  | **abstract** | **0.209** | **0.083** | **2.526** | **.013** |
|  | concrete | 0.139 | 0.098 | 1.414 | .161 |
|  | abstract*concrete | 0.06 | 0.057 | 1.068 | .288 |
| Multiple ways  *R*^2^ =.100 | (Intercept) | 3.723 | 0.104 | 35.653 | < .001 |
|  | abstract | 0.089 | 0.078 | 1.13 | .261 |
|  | **concrete** | **0.269** | **0.093** | **2.89** | **.005** |
|  | abstract*concrete | 0.027 | 0.054 | 0.504 | .615 |
| Search for a compromise *R*^2^ = .094 | (Intercept) | 3.888 | 0.105 | 36.878 | < .001 |
|  | abstract | 0.045 | 0.079 | 0.565 | .573 |
|  | **concrete** | **0.272** | **0.094** | **2.9** | **.005** |
|  | abstract*concrete | 0.004 | 0.054 | 0.078 | .938 |
| Self-transcendence  *R*^2^ =.131 | (Intercept) | 3.087 | 0.119 | 25.957 | < .001 |
|  | **abstract** | **0.302** | **0.089** | **3.388** | **.001** |
|  | concrete | 0.063 | 0.106 | 0.591 | .556 |
|  | abstract*concrete | 0.102 | 0.061 | 1.674 | .097 |

*Notes*. All continuous predictors are mean-centered.

Exploratory measure of pros and cons of possible behavior. We aimed to explore whether individual differences in abstract and concrete construal map on consideration of pros and cons for three different behaviors in response to the difficult social situation (*spending time with this person in other situations, talking about this person with someone else, avoiding this person entirely*). Per prior research ^6^, we aimed to use number of generated pros as an operationalization of *desirability* concerns and number of generated cons as an operationalization of *feasibility* concerns. As pre-registered, a research assistant blind to the hypothesis counted the number of pros (*M* = 4.59, *SD* = 2.03; correlation across three prompts: .04 < *r*s < .39) and cons (*M* = 4.66, *SD* = 2.20; correlation across three prompts: .40 < *r*s < .52).

### Frequency of considering pros and cons as an indirect measure of desirability and feasibility

If abstract and concrete construal are inverse poles of the same dimension, one would expect that abstract construal would be primarily linked with desirability focus and concrete construal would be primarily linked with feasibility concerns. However, is abstract and concrete construals are orthogonal dimensions and may co-occur, an alternative hypothesis would be that both desirability and feasibility considerations may be pronounced when the focus is on the concrete situation at hand. Correlations with individual items showing concrete thought vs. abstract thought as simultaneous predictors of wisdom are below.

|  | | Pros | Cons |
| --- | --- | --- | --- |
| **Abstract Construal Items** |  | -.241 | -.017 |
| I thought about why this event would occur |  | .068 | .159 |
| I saw the situation as an observer would |  | -.315^*^ | -.184 |
| I thought about the situation as one of many similar experiences |  | -.168 | .096 |
| **Concrete Construal Items** |  | .134 | .138 |
| I thought about how the event will unfold |  | .118 | .123 |
| I saw the situation through my own eyes |  | .307^*^ | .317^*^ |
| I thought about the situation as one-of-a kind, a unique experience |  | -.059 | -.053 |
| *Notes. p*-value adjustment method: Holm (1979). *. P < .05 (2-tailed). Data is rank-transformed prior to analyses to account for the ordinal nature of the pro/con index. | | | |

Two of the three concrete construal items were systematically positively associated with both pros and cons, whereas abstract construal items were largely negatively associated with both markers of desirability (pros) and feasibility (cons). Further, as results of multiple regression analyses below show, when both abstract and concrete construal scales were entered as simultaneous predictors of pros or cons, greater abstract construal was linked to a non-significant trend toward fewer pros listed.

|  | | *B* | *SE* | *Beta* | *t* | *p* |
| --- | --- | --- | --- | --- | --- | --- |
| DV: Total Number of Pros | |  |  |  |  |  |
|  | Abstract Construal | -.290 | .152 | -.192 | -1.90 | .060 |
|  | Concrete Construal | .254 | .174 | .147 | 1.46 | .147 |
| DV: Total Number of Cons | |  |  |  |  |  |
|  | Abstract Construal | -.073 | .168 | -.045 | -0.43 | .665 |
|  | Concrete Construal | .220 | .192 | .118 | 1.15 | .255 |

In sum, in Sample B we observed little evidence supporting the hypothesis that thinking in a more abstract [concrete] way about a difficult social interaction one anticipates made people consider various pros [cons] of strategies for dealing with the other person involved in this situation. Instead, abstract construal appeared negatively associated with the number of pro statements participants generated. However, note that it is unclear whether arguments pro/con a particular behavior in fact reflect desirability/feasibility concerns. Moreover, even if this operationalization of desirability/feasibility is accurate, it is unclear whether this measure is related to the social event: focus on desirability/feasibility of the offered solutions might not be linked to focus on desirability/feasibility of the event itself. The lack of correlation may be explained by the different target of the cognitions: the concrete and abstract construals referred to the social event, whereas the pros and cons referred to the three possible ways to deal with the person involved in the event. Therefore, we conceptually replicated this part of the study with a more direct measure of desirability and feasibility thoughts in Sample D.

## Pilot Study C

In the next sample we systematically varied explicit instructions to reflect on one’s goals in an abstract (“why”) fashion, concrete (“how”) fashion^4^ or both (“why”/ “how” or vice versa; order randomized). This experimental setup afforded simultaneous testing whether situational effects of construal on wisdom mirror individual difference effects. On the one hand, one could predict experimentally induced effects to be isomorphic to individual difference effects of abstract and concrete construal on wisdom: Instructions to reflect on one’s goals both abstractly and concretely would result in highest degree of wisdom in participants/ reflections. On the other hand, action identification theory ^9^ posits that in situations when both abstract and concrete construals are made salient, the former would dominate. Consequently, effects of abstract + concrete instructions would be indistinguishable from effects of abstract instructions and both types of instructions would produce effects that differ from the concrete instructions. This alternative prediction is also consistent with the idea that the experimental boosts of wisdom may be most impactful when shifting participants’ construal away from the baseline. The baseline when reflecting on social situations tends to be concrete ^10,11^, which was also evident in our Studies 1a-1b: Both studies indicated greater reports of concrete rather than abstract thoughts about a difficult social situation. Thus, when the baseline response is already concrete, abstract instructions may be relatively more effective than concrete instructions.

The data collection procedure, scale aggregation plan, and hypotheses were preregistered and are available on OSF ([https://osf.io/2sfxr](https://osf.io/2sfxr?view_only=6fbbaa64115e4e27ad3dbe047c7b594d)). We hypothesized that construal level dimensions would be independent dimensions, and that being high on both abstract and concrete thought would independently be linked with wise reasoning about interpersonal situations. We also preregistered to compare concrete thought, abstract thought, and wise reasoning between conditions.

Method

*Participants*. We aimed to recruit 230 participants, based on a priori power analysis (a medium effect seen in prior experimental CLT research, *f* = .25, in a one-way ANOVA with 5 groups would be detectable with 80% power in a sample of 200). We oversampled to account for exclusions. We recruited 237 U.S. MTurk workers and excluded ten participants who did not describe an anticipated social situation. The final sample included 227 participants who completed at least some measures and 218 participants who completed both construal- and wisdom-related measures (55.9% female, *M_age_* = 36.50, *SD* = 11.98).

*Procedure and Materials*. The procedure was identical to Sample B, with one exception: Before reflecting on a difficult social situation in the future, participants were randomly assigned to reflect on their physical fitness and health goals in an abstract (“why”, *n* = 50) fashion, concrete (“how”, *n* = 44) fashion ^4^or both (“why” then “how”, *n* = 43, (“how” then “why”, *n* = 38) or did not complete the goal reflection exercise (*n* = 43). For each exercise, participants wrote down increasingly abstract [concrete] thoughts why [how] they would maintain this goal in four textboxes connected by arrows ^4^.

Participants then completed the abstract and concrete construal items as in Sample B. Items within each construal group were significantly positively correlated. See Table S2 for verbatim items and Table S4 for correlations between items. As in samples a-b, participants reported more concrete (*M* = 5.25, *SD* = 1.13) than abstract (*M* = 4.62, *SD* = 1.24) construal in reflections on a difficult social situation, *t*(226) = 5.73, *p* < .001. Further replicating earlier results, analyses of self-reported construal in sample c showed little support for a unidimensional nature of the instrument: Abstract and concrete construal were not significantly correlated, *r* = .03, 95%*CI* [-.10, .16], *t*(225) = .40, *p*  = .691.

Participants then completed the wise reasoning index (Brienza et al., 2018). Examination of the factor loadings yield a predicted 5 factor solution, revealing a pattern that was largely consistent with prior research with a few minor exceptions: One of the intellectual humility items did not uniquely load on any of the components (Behave as if there may be some information to which I did not have access). A further intellectual humility item loaded on the search for compromise/conflict resolution dimension (Look for any extraordinary circumstances before forming my opinion). Further, two of the items concerning multiple ways to think about change (5. Look for different solutions as the situation evolved; 6. Consider alternative solutions as the situation evolved) loaded on search for compromise and conflict resolution factors, similar to earlier studies. Notably, factor correlation matrix shows a consistent set of positive correlations between each of the sub-factors in a medium-high range, .329 <*r*s < .535.

**5 component factor solution of wise reasoning items**

|  | Factor | | | | |
| --- | --- | --- | --- | --- | --- |
|  | 1 | 2 | 3 | 4 | 5 |
| 1. Put myself in the other person's shoes | -.019 | -.105 | -.085 | -.073 | **-.825** |
| 2. Try to communicate with the other person what we might have in common | .102 | .151 | -.031 | -.070 | **-.490** |
| 3. Make an effort to take the other person's perspective | .067 | .191 | -.021 | .138 | **-.730** |
| 4. Take time to get the other person's opinions on the matter before coming to a conclusion | .063 | .079 | .134 | -.231 | **-.638** |
| 5. Look for different solutions as the situation evolved | .108 | **.707** | .053 | -.196 | .085 |
| 6. Consider alternative solutions as the situation evolved | .213 | **.590** | .088 | -.268 | .106 |
| 7. Believe the situation could lead to a number of different outcomes | **1.045** | -.085 | .040 | -.029 | -.004 |
| 8. Think the situation could unfold in many different ways | **.534** | .100 | -.128 | .073 | -.034 |
| 9. Double-check whether my opinion on the situation might be incorrect | -.024 | .085 | -.173 | **-.715** | -.075 |
| 10. Double-check whether the other person's opinions might be correct | .044 | .027 | -.035 | **-.747** | -.114 |
| 11. Look for any extraordinary circumstances before forming my opinion | -.055 | **.435** | -.198 | -.148 | -.032 |
| 12. Behave as if there may be some information to which I did not have access | .195 | .059 | -.166 | -.151 | -.138 |
| 13. Try my best to find a way to accommodate both of us | .043 | **.673** | -.037 | .115 | -.189 |
| 14. Though it may not be possible, I would search for a solution that could result in both of us being satisfied | -.045 | **.812** | .020 | .009 | -.061 |
| 15. Consider first whether a compromise was possible in resolving the situation | .001 | **.790** | -.030 | .023 | -.028 |
| 16. View it as very important that we resolve the situation | .106 | **.350** | -.063 | .017 | -.272 |
| 17. Try to anticipate how the conflict might be resolved | .098 | **.480** | -.161 | .035 | -.154 |
| 18. Wonder what I would think if I was somebody else watching the situation | .051 | -.034 | **-.863** | -.010 | .034 |
| 19. Try to see the conflict from the point of view of an uninvolved person | .001 | .075 | **-.683** | -.065 | -.080 |
| 20. Ask myself what other people might think or feel if they were watching the conflict | .033 | -.074 | **-.923** | -.025 | -.001 |
| 21. Think about whether an outside person might have a different opinion from mine about the situation | -.012 | .049 | **-.837** | .001 | .039 |

Results. Following pre-registered analytical plans, we examined the effect of condition, finding no effect of condition on abstract thought index, *F*(4,215)=0.66 *p*=.620, *ɲ*^2^ =.012, or on concrete thought index, *F*(4,215)=0.51 *p*=.729, *ɲ*^2^ =.009. The manipulation was not successful in introducing group differences in self-reported abstractness or concreteness in reflections on a difficult social situation, abstract: *F*(3, 223) = 0.61, *p*  = .607, pairwise post-hoc comparisons: .10 < |*t*s| ≤ 1.12, *p > .*676; concrete: *F*(3, 223) = 0.56, *p*  = .644, pairwise post-hoc comparisons: .29 < |*t*s| ≤ 1.13, *p > .*670 (see Figure S1 below for means in each conditions). We conclude that the manipulation of thoughts in the health domain did not transfer to thoughts about the difficult personal situation.


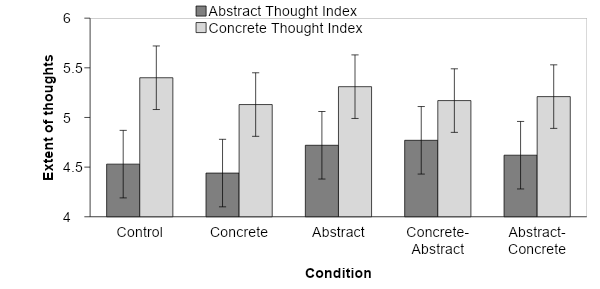


Figure S1. Construal means by condition in Pilot Study C. Standard Error bars denote 95% confidence intervals. Control = No thought exercise, Abstract = Abstract mindset exercise, Concrete = Concrete mindset exercise, Concrete-abstract = Thought exercise to induce concrete mindset followed by thought exercise to induce abstract mindset, Abstract-concrete = Thought exercise to induce abstract mindset followed by thought exercise to induce concrete mindset.

Following the pre-registered plan, we nevertheless continued to examine group differences in wisdom. Per pre-registration, we combined two different orders of the abstract+ concrete condition). Planned contrasts indicated no significant difference between abstract+concrete and abstract conditions, *t*(214) = 0.78, *p* = .437, but a trend of participants in the abstract+concrete condition reporting wiser reasoning compared to the concrete condition, *t*(214) = 1.62, *p* = .107 (see Figure S2 below for means in each conditions). As predicted, we observed a significant difference between abstract+concrete condition and the no exercise control condition, *t*(214) = 2.21, *p* = .028. Moreover, conditions that included an abstraction-inducing exercise showing significantly greater wisdom as compared to only-concreteness-inducing and (concrete baseline) control conditions, *t*(214) = 3.07, *p* = .002. In other words, we did not observe isomorphism between individual difference of abstract and concrete construal on wisdom and experimentally induced effects. Instead, the baseline response to the difficult social situation was concrete, and abstraction-inducing instructions were relatively more effective than concreteness-inducing instructions. Notably, lack of evidence that the manipulation successfully produced group differences in situation-level construal effects (consistent with Maier et al., 2022) renders this interpretation about downstream group differences in situation-level wisdom effects inconclusive.


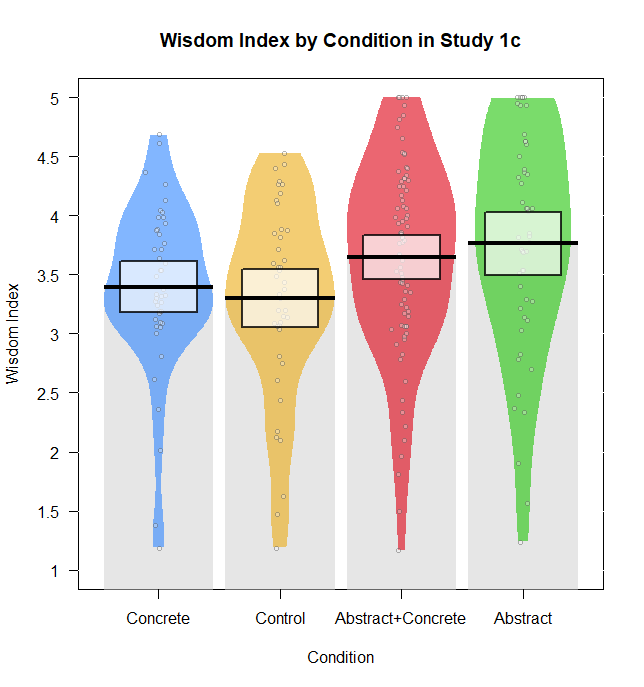


Figure S2. Wisdom means by condition in Pilot Study C.

Wisdom index (5-point scale) as a function of abstract, concrete, both exercises and no exercises (control) in Sample C. Pirateplot showing group means, distribution of the data via density violin plots, and 95% confident band. Plots sorted from lowest to highest on the wisdom index.

Next, we examined how indices of abstract and concrete construal indices relate to wise reasoning in regression analyses (also preregistered), while aggregating across conditions. Results of multivariate analysis with abstract and concrete construals and their interaction term as predictors of wisdom revealed a significant effect of concreteness and a significant a significant abstract × concrete interaction (see Table S8). Simple slope analyses showed that participants who reported higher scores on both abstract and concrete construals scored highest on the overall wisdom scale (see Figure S3). Specifically, abstract construal was linked with wisdom when concrete construal was high (+1*SD*), *B* = 0.127, *SE* = 0.051, *t* = 2.473, *p* = .014, but was not linked with wisdom when concrete construal was low (-1*SD*), *B* = -0.090, *SE* = 0.069, *t* = 1.316, *p* = .190. These results were comparable across all features of wisdom and when including mindset condition as a covariate. Mindset condition did not significantly interact with either construal type or their interaction, *F*s < 1.

## Table S8: Construal indices and wisdom in Sample C

|  |  | *Est.* | *S.E.* | *t* | *p* |
| --- | --- | --- | --- | --- | --- |
| Wisdom Index  *R*^2^ = .110 | (Intercept) | 3.550 | 0.054 | 65.214 | < .001 |
|  | abstract | 0.018 | 0.046 | 0.396 | .692 |
|  | **concrete** | 0.196 | 0.048 | 4.066 | **< .001** |
|  | **abstract*concrete** | 0.096 | 0.035 | 2.756 | **.006** |
| Other perspectives *R*^2^ = .098 | (Intercept) | 3.534 | 0.068 | 51.598 | < .001 |
|  | abstract | 0.045 | 0.058 | 0.784 | .434 |
|  | **concrete** | **0.185** | **0.061** | **3.057** | **.003** |
|  | **abstract*concrete** | **0.138** | **0.044** | **3.148** | **.002** |
| Intellectual humility  *R*^2^ = .058 | (Intercept) | 3.242 | 0.068 | 47.391 | < .001 |
|  | abstract | 0.091 | 0.058 | 1.57 | .118 |
|  | **concrete** | **0.142** | **0.06** | **2.35** | **.020** |
|  | abstract*concrete | 0.071 | 0.044 | 1.632 | .104 |
| Multiple ways *R*^2^= .113 | (Intercept) | 3.646 | 0.062 | 59.16 | < .001 |
|  | abstract | -0.027 | 0.052 | -0.519 | .604 |
|  | **concrete** | **0.249** | **0.055** | **4.574** | **< .001** |
|  | **abstract*concrete** | **0.095** | **0.039** | **2.412** | **.017** |
| Search for a compromise *R*^2^ = .074 | (Intercept) | 3.778 | 0.064 | 58.674 | < .001 |
|  | abstract | -0.036 | 0.054 | -0.665 | .507 |
|  | **concrete** | **0.206** | **0.057** | **3.625** | **< .001** |
|  | **abstract*concrete** | **0.079** | **0.041** | **1.93** | **.055** |
| Self-transcendence  *R*^2^ = .047 | (Intercept) | 3.337 | 0.077 | 43.092 | < .001 |
|  | **abstract** | **0.166** | **0.065** | **2.537** | **.012** |
|  | **concrete** | **0.113** | **0.068** | **1.646** | **.101** |
|  | abstract*concrete | 0.011 | 0.049 | 0.218 | .827 |

*Notes*. All continuous predictors are mean-centered.

## Pilot Study D

In prior samples, we examined individual differences in real life events participants either had experienced or were expecting to experience. Thus, the effects could be partially explained by individual differences in types of events people chose to reflect on: Maybe some events were more likely to foster intellectual humility, open-mindedness to multiple ways a situation may unfold, or consideration of others’ perspectives, and such events were also conducive to abstract and concrete construal; in short, the association between construal and wisdom may be due to specific types of events people brought to mind as a third variable. To control for this possibility, in Sample D we examined the relationship between abstract and concrete construal and wisdom when reflecting on standardized scenarios used in earlier research on construal levels ^5,12^: Participants contemplated decision situations involving consumer choice (e.g., choosing between restaurants, phone apps, shelving units).

Sample D further aimed to rule out the possibility that the positive associations of both abstract and concrete construal to wisdom are due shared method variance or general response tendencies. To this end, we examined thoughts about desirability and feasibility of decisions. Based on earlier research (Trope & Liberman, 1998), we expected that a focus on the desirability of an event may be predicted uniquely by abstract construal, while a focus on the feasibility of the event may be predicted by concrete construal. If these outcome variables are uniquely predicted by one of the construal types, and not the other, while both construal types positively relate to wisdom, a general response tendency account may be unlikely.

The data collection procedure, scale aggregation plan, and hypotheses were preregistered and are available on OSF (https://osf.io/gupfj). We hypothesized that construal level dimensions would be independent dimensions, that being high on both abstract and concrete thought would independently be linked with wise reasoning about interpersonal situations, and that abstract construal would be primarily linked with a focus on desirability aspects whereas concrete construal would be primarily linked with a focus on feasibility aspects.

### Method

*Participants*. We aimed to recruit 100 per each of the three decision scenarios. This estimate followed Pilot Study B and was guided by an a priori power analysis (a small regression effect, *f* = .10, would be detectable with 80% power in a sample of 80; due to lack of theoretical reasons, we did not consider possible interaction effects between scenario type and predictor variables). We oversampled to account for exclusions, posting 300 rather than 240 participation spots in total. We recruited 313 U.S. MTurk workers (compensated with $0.50). Of these, we excluded 43 participants for not following instructions (they did not provide an explanation for their decision, which was a preregistered attention check). The final sample included 270 participants (*M*_age_ = 36.58, *SD* = 11.68; 40.7% female).

*Procedure*. Participants were randomly assigned to think about one of three hypothetical decision situations from Sanchez and Ledgerwood^12^: choosing between two restaurants (*n* = 75), choosing a shelving unit (*n* = 100), choosing a photo app for their phone (*n* = 95). For example, for the restaurant choice situation, they were asked to

“Imagine that your bank’s rewards program sends you a coupon for a free meal from your choice of a list of restaurants. As you consider which restaurant to select, you notice that the options vary in how much you will like the food as well as how easy it will be for you to get to the restaurant.”

Note that we planned to give all three scenarios to everyone in a within-participant design, but a mistake in survey programming led to participants only receiving two out of three scenarios. Instead, we analysed the first scenario participants saw in a between-subject design, an analysis that we intended to do anyways (i.e., this analysis was preregistered).

*Materials*. Following previous sample procedures, we assessed participants' construal levels while imagining the scenario. Participants completed three items assessing *abstract construal* inter-item -.07 < *r*s ≤ .32) and three items assessing *concrete construal*, as in Sample B and C (See Table S2 for verbatim items). Items within each construal group were significantly positively correlated, with one exception: thinking about the reasons for the event was unrelated to viewing the event from a third-person perspective, *r* = -.071.

Next participants reported their *focus on the feasibility* of the chosen option (e.g., "In this situation, how important would it be to you that the restaurant you choose is easy for you to get to") and *focus on the desirability* of the chosen option (e.g., "In this situation, how important would it be to you that the restaurant you choose has food that you really like") in the decision dilemma on a scale ranging from *Not Important at all* (1) to *Very Important* (7).^^[[1]](#footnote-1)^^

Finally, participants completed an abbreviated measure of wisdom with eight items that assessed wisdom-related features in response to the decision situation on 5-point scales. These eight items were adopted from the SWiS scale^7^ and captured facets of intellectual humility, multiple ways, search for compromise, and whether they adopted a self-transcendent view. We did not include items targeting whether participants took the perspective of the other person because the decision dilemmas did not involve another person. Like in previous samples, we averaged all items into respective facets and subsequently averaged all facets, but self-transcendence into an overall wisdom index (inter-facet reliability *α* = .72). Confirmatory factor analyses confirmed the factor structure, with medium-high correlations between factors, .33 ≤ *r*s ≤ .55.

|  | **Confirmatory 4 component solution** | | | | | | | |  |
| --- | --- | --- | --- | --- | --- | --- | --- | --- | --- |
|  |  | Factor | | | | | | | |
|  |  | 1 | | 2 | | 3 | | 4 | |
| MULTIPLE.WAYS - Believed the situation could lead to a number of different decisions | | -.050 | **.732** | | -.014 | | .120 | | |
| MULTIPLE WAYS - Thought the situation could unfold in many different ways | | .017 | **.879** | | .018 | | -.052 | | |
| INT. HUMILITY - Double-checked whether my initial opinion on the situation might be incorrect | | .005 | .011 | | .042 | | **.741** | | |
| INT. HUMILITY - Behaved as if there may be some information to which I did not have access | | .072 | .042 | | -.151 | | **.462** | | |
| COMPROMISE - Considered first whether a compromise was possible in making a decision | | -.042 | -.035 | | **-.916** | | .049 | | |
| COMPROMISE - Tried to anticipate how the conflict between the two options might be resolved | | .213 | .227 | | **-.391** | | -.014 | | |
| SELF-TRANSCEND - Tried to see the decision from the point of view of an uninvolved person | | **.784** | -.027 | | -.029 | | .070 | | |
| SELF-TRANSCEND - Thought about whether an outside person might have | | **.988** | -.003 | | .046 | | -.017 | | |
|  | Extraction Method: Maximum Likelihood. Rotation Method: Oblimin with Kaiser Normalization. | | | | | | | |  |

Analytical Procedure. Following pre-registered analytical plans, we relied on correlational and linear regression analyses. The regression coefficients predicting wise reasoning from both abstract and concrete construal indices are shown in Table S9.

## Table S9: Construal indices and wisdom in Sample D

|  |  | *Est.* | *S.E.* | *t* | *p* |
| --- | --- | --- | --- | --- | --- |
| Wisdom Index *R*^2^ = .283 | (Intercept) | 3.086 | 0.046 | 67.327 | < .001 |
|  | **abstract** | **0.249** | **0.047** | **5.258** | **< .001** |
|  | **concrete** | **0.249** | **0.039** | **6.466** | **< .001** |
|  | abstract*concrete | 0.017 | 0.027 | 0.63 | 0.529 |
| Other perspectives *R*^2^ = .183 | (Intercept) | 2.325 | 0.071 | 32.519 | < .001 |
|  | **abstract** | **0.332** | **0.074** | **4.496** | **< .001** |
|  | **concrete** | **0.253** | **0.060** | **4.204** | **< .001** |
|  | abstract*concrete | **0.09** | **0.043** | **2.114** | **.035** |
| Intellectual humility  *R*^2^ = .195 | (Intercept) | 2.955 | 0.061 | 48.61 | < .001 |
|  | **abstract** | **0.235** | **0.063** | **3.741** | **< .001** |
|  | **concrete** | **0.270** | **0.051** | **5.277** | **< .001** |
|  | abstract*concrete | 0.071 | 0.036 | 1.951 | .052 |
| Multiple ways  *R*^2^ = .142 | (Intercept) | 3.329 | 0.059 | 56.222 | < .001 |
|  | **abstract** | **0.23** | **0.061** | **3.758** | **< .001** |
|  | **concrete** | **0.182** | **0.050** | **3.654** | **< .001** |
|  | abstract*concrete | -0.033 | 0.035 | -0.945 | .345 |
| Search for a compromise *R*^2^ = .221 | (Intercept) | 2.974 | 0.063 | 47.186 | < .001 |
|  | **abstract** | **0.282** | **0.065** | **4.334** | **< .001** |
|  | **concrete** | **0.296** | **0.053** | **5.583** | **< .001** |
|  | abstract*concrete | 0.014 | 0.038 | 0.380 | .704 |
| Self-transcendence  *R*^2^ = .183 | (Intercept) | 2.325 | 0.071 | 32.519 | < .001 |
|  | **abstract** | **0.332** | **0.074** | **4.496** | **< .001** |
|  | **concrete** | **0.253** | **0.060** | **4.204** | **< .001** |
|  | **abstract*concrete** | **0.090** | **0.043** | **2.114** | **.035** |

*Notes*. All continuous predictors are mean-centered.

In this sample, we also examined the possibility of a common method bias impacting positive associations between both types of construal and wisdom. To this end, we aimed to explore whether concrete and abstract thought uniquely predicted participants' feasibility and desirability concern, respectively. In multiple regressions, we regressed the ratings for feasibility or desirability on both concrete and abstract thought (controlling for decision scenario). The ratings of concrete thought uniquely predicted feasibility concerns, *B =* .34 *95%CI*[.21;.47], *t*(266)= 5.01, *p* < .001, whereas the ratings of abstract thought did not, *B =* .10 *95%CI*[-.07;.25], *t*(266) = 1.17, *p =* .244. In contrast, the ratings of abstract thought significantly predicted desirability concerns, *B =* .32 *95%CI*[.11;.53], *t*(266) = 2.95, *p=* .003, whereas ratings of concrete thought did not, *B =* .15 *95%CI*[-.03;.32], *t*(266) = 1.66, *p =* .098. Demonstrating these outcome-specific patterns of concrete and abstract thought provides some evidence of the discriminant validity of the construal measures, suggesting that their additive effects on wise reasoning are not due to a general response tendency or shared method variance.

## Mega-analysis of Pilot Studies

This analysis was not preregistered. We first report results of a mega-analysis across four samples via a linear mixed model with responses nested in samples (random intercept models). To assess heterogeneity and robustness across samples, we follow-up with sample-specific meta-analytic estimates from Schwarzer’s ^13^ *meta* package in *R*.

Results of a linear mixed model with abstract and concrete construal scores nested in participants and in samples showed that participants reported significantly more concreteness (*M* = 4.98, 95%*CI*[4.67, 5.30]) than abstractness (*M* = 4.46, 95%*CI*[4.15, 4.77]), *t*(914) = 10.44, *p* < .001, *Cohen’s d* = 0.488. Overall, abstract and concrete construal were significantly positively associated with each other, *r* = .23, 95%*CI*[.16, .31], *t*(823.40) = 6.03, *p*  < .001. Correlations were positive in each sample, with effect size ranging from moderate, *r*_a_  = .24, *r*_d_  = .34, to negligible, *r*_b_  = .08, *r*_c_  = .03. These results suggest that abstract and concrete construal are not opposite poles of the same dimension.

In a subsequent linear mixed effects analysis, we used mean-centered abstract and concrete construal scores and their interaction as predictors of mental features of wisdom. The results indicated a significant positive effect of both construal types, abstract: *B* = .150, *SE* = .023, *t*(909.91), *p* < .001; concrete: *B* = .219, *SE* = .023, *t*(908.10), *p* < .001, and a non-significant trend in the interaction, *B* = .027, *SE* = .015, *t*(908.61), *p* = .069. Simple slope analyses indicated that the effect of abstract construal on wisdom was stronger when concrete construal was also high (+1 *SD*), *B* = .182, *SE* = .027, *t* = 6.83, *p* < .001, compared to when it was low (-1 *SD*), *B* = .118, *SE* = .031, *t* = 3.77, *p* < .001. Critically, these effects hold when controlling for length of the open-ended reflections, suggesting that the positive association between both types of construal and wisdom is not due to overall deliberation tendencies (see Figure S3 for overall estimates and consistent effects for each sample).


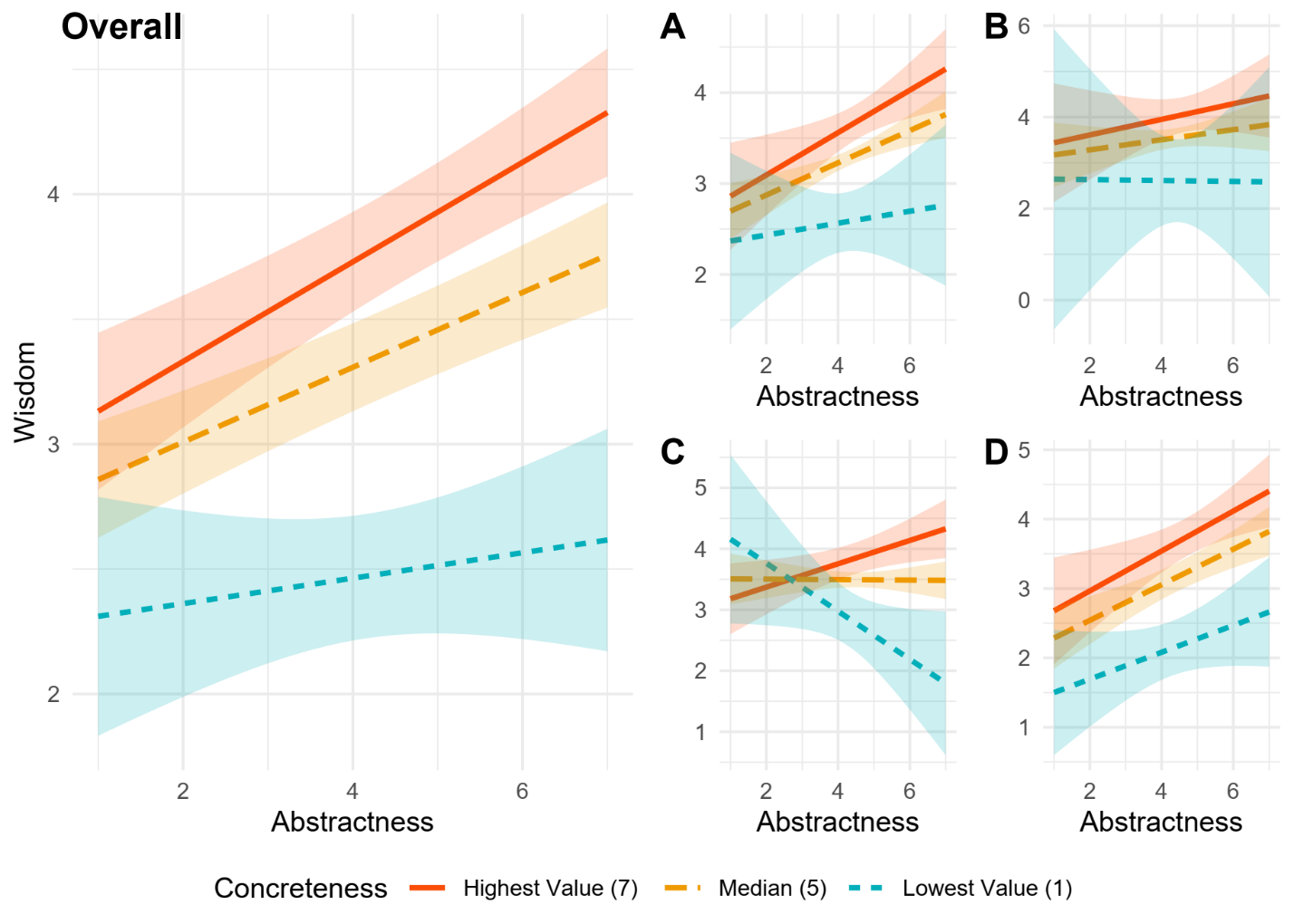


Figure S3: Estimates from the mega-analysis of pilot study results.

Wisdom as a function of concrete and abstract construal (7-point scale) for participants in the highest (7), median (5) and lowest (1) level of concreteness. Estimates represent simple slopes at specified levels of concreteness with robust 95% confidence bands based on results of a model with mean-centered abstractness and concreteness scores, their interaction, and reflection word count as predictors. For plotting, estimates are reversed to original scale. Overall = estimates from a mega-analysis with sample as random intercept. A-D = sample-specific estimates. Sample a aggregates across two temporal conditions and Sample c aggregates across groups performing different construal exercises. In each sample, highest wisdom scores were observed among participants reporting *both* high abstractness and concreteness.

Next, we meta-analyzed results from the four samples. First, we calculated partial correlations, controlling for opposite construal type (i.e., abstract construal and wise reasoning controlling for concrete construal, and concrete construal and wise reasoning controlling for abstract construal). We used the fixed and random effects in which the mean effect size (i.e., mean correlation) was weighted by sample size, Fisher’s *z* transforming correlations for analyses and converting them back to Pearson correlations for presentation. As Figure S4 shows, both abstract and concrete construal have shown significant effects in the moderate range, such that both abstract, *M_r_* = .233, 95% *CI* [.171; .294], *z* = 7.14, *p* < .001, and concrete construal were related to wiser reasoning, *M_r_* = .282, 95% *CI* [.221; .341], *z* = 8.71, *p* < .001.

Figure S4. Meta-analytic estimates of the partial correlation between construal and wisdom

**ABSTRACT CONSTRUAL**


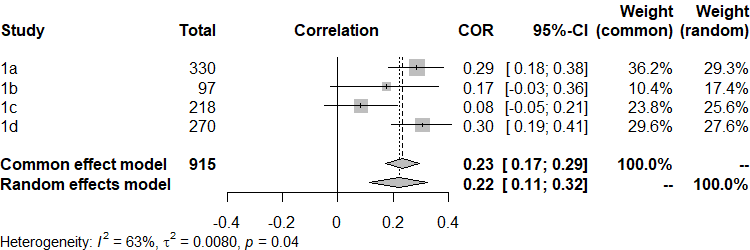


**CONCRETE CONSTRUAL**


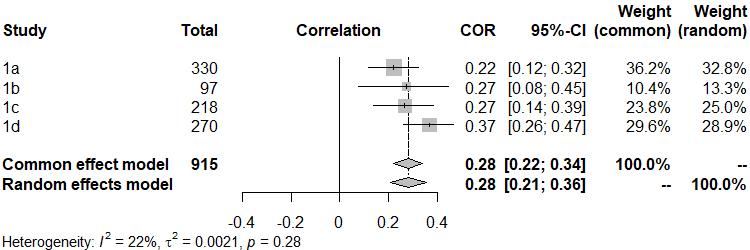


*Note*. Analyses are based on the Inverse variance method and DerSimonian-Laird estimator for τ^2^.



## Discussion of Pilot Study Results

Across four samples capturing autobiographical events, anticipated social challenges, and decision scenarios, we observed systematic evidence that individual differences in self-reported abstract and concrete construal are not inverse poles of the same abstractness-concreteness dimension. Furthermore, abstract and concrete construal were both positively associated with mental features of wisdom—individuals showing greater intellectual humility, recognition of change, perspective-taking, and preference for compromise tended to represent the situation both abstractly and concretely. This result occurred independently of the reflective effort, and for different types of content (autobiographical or anticipated social conflicts, hypothetical decision scenarios). Critically, while greater abstractness and concreteness jointly related to mental features of wisdom, abstractness was uniquely associated with desirability concerns, and concreteness was uniquely associated with feasibility concerns. These latter observations are in line with the Construal Level Theory ^14^. They provide support for the discriminant validity of the construal measures and suggest that the additive effects of construals on wisdom are not due to common method (similar questionnaire format) or common factor variance (e.g., greater deliberation). If a common method factor was the reason for the additive association of abstract and concrete construal to wisdom, the pattern of results should have been mirrored for feasibility and desirability goals. While the results from pilot studies were encouraging, and effects of construal on wisdom were mostly consistent across different items making up the construal indices (see Figure S1), the indices of self-reported construal showed modest reliability. Moreover, it is possible the way we operationalized the items does not correspond to the way construal theory researchers would define or operationalize central individual differences in construal. We addressed this limitation in Study 1.

# Study 1

## Study 1a Analytical steps

Analytical procedure. Prior to psychometric analyses we eliminated 3 close-to-identical sounding items (#abstract 16 - too similar to abstract 17; #abstract 29 - too similar to abstract7; #concrete 23 - too similar to concrete 1), keeping only one version of each item in further analyses. Next, we used Exploratory Factor analysis (EFA) using *MinRes* (minimum residual) to identify factors and select items. We selected EFA because, unlike PCA, it assumes an underlying latent (psychological / causal) structure influencing similarities in responses to different factors of items. We used *MinRes* because it produces solutions very similar to maximum likelihood even for badly behaved matrices. Theory guiding construction of our items suggested that such latent variables (e.g., abstractness and concreteness) could drive individuals’ construal when reasoning about a social conflict. Therefore, EFA appeared well-suited for our goal of item reduction.

Evaluating correlation matrix. Kaiser-Meyer-Olkin factor adequacy tests suggested a high level of Measure of Sampling Adequacy (overall MSA = .94; only two items with MSA between .85 and .86, while other items MSA > .90). Similarly, Bartlett’s test of sphericity was significant, χ^2^ = 10,557.62 (*df* = 1953), *p* < .001, suggesting that the variables in the data set were significantly different from the identity matrix. Consequently, the data appeared sufficiently variable to perform exploratory factor analyses.

Detecting multivariate outliers. We examined Mehalanobis distance for multiple variables. Following Tabachnick aid Fidell ^15^, we are not using *N* - 1 for *df* because Mahalanobis distance is evaluated with degree of freedom based on the number of variables. Thus, using the criterion of α = .001 with 63 *df* (number of variables), the critical χ^2^ to exceed is 103.44. Using this criterion, we detected 29 multivariate outliers, leaving 293 non-outlying cases– an acceptable number of cases for exploratory factor analyses. Preliminary results without omitting multivariate outliers were very similar to the results reported below.

Number of factors. First, we inspected eigenvalues, followed by screeplot and parallel analyses. Eigenvalues for the first three factors were larger than two, and after the fifth factor, changes in successive eigenvalues appeared small. We took it as initial evidence that there may be between three and five factors. Additionally, based on the theoretical rationale about two aspects of construal employed in prior research (abstractness and concreteness), we started with a forced 2-factor solution.

Scree plot further showed that after four factors the curve of plotted eigenvalues began to straighten out, suggesting that four is the maximum number of factors to extract. Similarly, parallel analysis suggested that the distance between eigenvalues from the actual and simulated data was smallest at four factors, while parallel analysis itself suggested five factors. Therefore, in the next step we probed two-, four-, and five-factor solutions, balancing model fit and interpretability.

We inspected each EFA (*MinRes*) model, allowing for correlation between factors (oblimin rotation). We considered solutions that maximize model fit and provide most interpretable solution (reasonably small number of cross-loadings). We looked for factor structure items which cross-loaded strongly onto more than one factor; we examined items with loadings < .3 and counted cross-loadings. Hereby, a two-factor solution provided the worst fit (*RMSR* = .05; *TLI* = .799; *RMSEA* = .052), with three cross-loadings. A four-factor solution provided a moderately adequate fit (*RMSR* = .04; *TLI* = .865; *RMSEA* = .042), with and seven cross-loadings. Finally, a five-factor solution provided a modest improvement in model fit (*RMSR* = .04; *TLI* = .893; *RMSEA* = .038), with 10 cross-loadings. Because the four-factor solution provided the best balance of cross-loadings and model fit, we chose to continue with it for further analyses.

Initial item selection. We looked for factor structure items which cross-loaded strongly onto more than one factor; we planned to cut any items with < .3 loading and that cross-loaded on more than one factor. Because we were agnostic about possible nature of association between abstract and concrete construal, we allowed the latent factors to correlate via an oblique (oblimin) rotation.

- Round 1: The matrix in Table S9 identified seven items with suboptimal cross-loadings, and two items with very low loadings. These items were removed before performing a subsequent item-reduction test.
- Round 2: The matrix reported in Table S10 revealed 2 items with suboptimal cross-loadings, and one item with very low loadings. These items were removed before performing the subsequent iteration of the item-reduction procedure.
- Round 3: The matrix in Table S11 revealed two items with suboptimal cross-loadings and two items with very low loadings. These items were removed before performing the subsequent iteration of the item-reduction procedure.
- Round 4: The matrix in Table S12 showed that all item loadings were above .3, with no cross-loadings. Thus, we stopped the process of item iteration. Item set was reduced to 47 items (20 abstract items and 27 concrete items). Inspection of factor loadings revealed that the *first factor* was dominated by items from the concrete pool that concerned the *specific details* of the experience, while the *second factor* was dominated by items focusing on *third person distance and* stereotypicality. Further, the *third* factor chiefly consisted of items focusing on the feelings and intuitions, while the *fourth* *factor* chiefly consisted of items focusing on the *broader meaning of the situation.*
- Round 5: We selected top 20 items (10 from the abstract pool and 10 from the concrete pool) and inspected loadings of these items (Table S13). Specifically, we selected all six items that uniquely loaded on factor 3 to represent abstractness and all four items that uniquely loaded on factor 4 to represent concreteness. To supplement these items, we selected four abstract items with highest loading on factor 2, and six concrete items with highest loadings on factor 1. We cross-validated the EFA loadings of the four-factor solution on the 20-item pool.

Expert feedback. In parallel to psychometric analyses, we reached out to six experts in construal level theory, asking them to review the initial set of items in terms of their fit for capturing abstract and concrete construal in the context of reflecting on challenging social situations and to identify top ten items they considered best capturing abstract and concrete construal, each. One expert provided general qualitative feedback, pointing out that some items captured focus emotional experiences and intuitions and that such focus may capture a separate (albeit related) construct concerning experiential focus rather than concrete construal per se. Qualitative feedback from two other experts was similar, and consequently (in Study 1b) we treated experience- and intuition-related items identified in psychometric analyses as a separate exploratory state-level measure within the nomological network analyses. Further, two experts indicated that items related to psychological distance may be viewed as distinct from abstract construal per se and hence we treated these items as another state-level marker of nomological net. In short, we removed experience and distance-related items prior to further analyses.

Experts also highlighted items that were previously eliminated in the iterative EFAs. Therefore, we counted how many times experts mentioned the remaining items in their feedback, zeroing in on items that satisfied the following criteria: a. they were mentioned by at least one expert; b. they showed a loading above .3 on one factor in psychometric analyses above; and c. they did not include cross-loadings in prior psychometric analyses above. We also ensured to keep the same number of items for abstractness and concreteness. Complete information about expert recommendations on item fit is on Open Science Framework ([https://osf.io/mwcyp/](https://osf.io/mwcyp/?view_only=1763d9a168bf48ee88aa466cc45c020b)). Through this process, we identified 8 additional items (11, 26, 28 from the abstract pool, and items 10, 12, 14, 22, 27 from the concrete pool) as theoretically central to the construct. Consequently, we included these items in subsequent analyses.

Based on expert’s feedback and initial psychometric investigation, we ended up with 22 items (11 abstract and 11 concrete). As Table 2 in the main manuscript shows, the abstract items systematically related to “why” questions, typicality, one’s character, extrapolation to one’s general future, the “big picture”, the wholistic perspective, and the general significance of the situation. Conversely, the concrete items reflected the “how” questions, specific behaviors, qualifiers of time, concrete experiences and circumstances, and concrete outcomes of the situation.

Fit of the structural model for the items selected in Study 1a. Because no prior psychometric work has supported a unidimensional abstract-concrete model, we compared the fit of a unidimensional model and a two-factor model. We assessed model fit with standard criteria using standardized root-mean-square residual (*RMSR* < .10), root-mean-square error of approximation (*RMSEA* < .08), and comparative fit index (*CFI* > .90) ^16,17^. According to these guidelines, CFA that a unidimensional model was poor, *RMSR* = .071, *RMSEA* = .089, *CFI* = .793, and significantly worse than the fit of a 2-factor model, *RMSR* = .065, *RMSEA* = .074, *CFI* = .856, χ ^2^(*df* = 1), *p* < .001. Moreover, inspecting loadings of the unidimensional model suggested positive contribution of all items, contrary to the claims that that features of abstract and concrete construal are opposite ends of the same construal dimension.

A 2-factor model was statistically superior to a unidimensional model. Further, fit indices suggest that the model could be improved. To examine potential adjustments, we next examined modification indices to further strengthen the model fit by allowing some error terms to covary. Modification indices in Table S14 indicates three pairs of error terms (highlighted in grey) with relatively high modification indices (> 15), suggesting that covarying these error term-pairs could improve the model fit. After covarying error terms, we re-examined the model fit, observing an improved fit, *RMSR* = .054, *RMSEA* = .060, *CFI* = .908.

## Study 1b

Further item selection and bifactor model**.** First, we tested a theorized 2-factor model of abstract and concrete construal as separate factors on the North American sample recruited via MTurk. Because initial bifactor 2-factor model with 22 items showed poor fit, *RMSR* = .057, *RMSEA* = .071, *CFI* = .863, we followed the pre-registered plan and examined modification indices, constraining three residual covariances with relatively high modification indices (>15). This procedure resulted in a modestly improved model fit, *RMSR* = .052, *RMSEA* = .060, *CFI* = .905. Consequently, we further followed the pre-registered contingency plan and performed a new series of iterative EFAs, with an eye on trimming items cross-loading across two factors. Like in Study 1a, we performed iterative analyses in several rounds.

- Round 1: First, we performed parallel analyses to identify number of factors and inspect factor loadings (Table S15). Theoretically, we aimed for a two-factor solution, while a scree plot suggested a three-factor solution. Because model fit was comparable, we inspected factor loadings of each solution, each showing one cross-loading item. Because a 2-factor solution was more parsimonious and all items but one (abstract 31) clearly loaded either on the abstract or concrete factors, we went ahead with a 2-factor solution in the next round. At this stage, we cut abstract item 31 due to a cross-loading, abstract item 12 (due to thematic similarity to abstract item 15, and because it showed poorer loading of the two) and concrete item 4 (due to lowest loading).
- Round 2: The matrix reported in Table S16 revealed one item with a low loading, which was removed to ensure balanced set of items per factor.
- Round 3: The matrix in Table S17 revealed an 18-item simple solution, with each item loading either on abstract or concrete factors.

After reducing the set of items, we moved to evaluating the model fit. *CFA* results on the MTurk sample showed that a bifactor 2-factor solution on the reduced set of 18 items showed a moderate fit, *RMSR* = .052, *RMSEA* = .067, *CFI* = .900. Therefore, we examined modification indices on the reduced 18-item solution, constraining two residual covariances with high modification indices (> 20; see Figure 3 in the main text). This procedure resulted in an acceptable model fit, *RMSR* = .050, *RMSEA* = .059, *CFI* = .923. Tables S18-S19 show the estimated unstandardized parameters for the final model in the North American and UK samples (see Figure 3 in the main text for standardized parameter estimates).

Inter-individual variability. We examined the distribution of abstract and concrete construal responses by taking the average across responses from nine items of each sub-scale. As Figure S5 shows, both construal types showed modest degree of skewness and kurtosis (both < |1|), with substantial inter-individual variability in responses across participants. Moreover, replicating pilot study results, people in North America and in the UK reported significantly greater concreteness than abstractness in their reflections on interpersonal challenges, North America: *t*(321) = 9.63, *p* < .001; UK: *t*(237) = 8.71, *p* < .001.


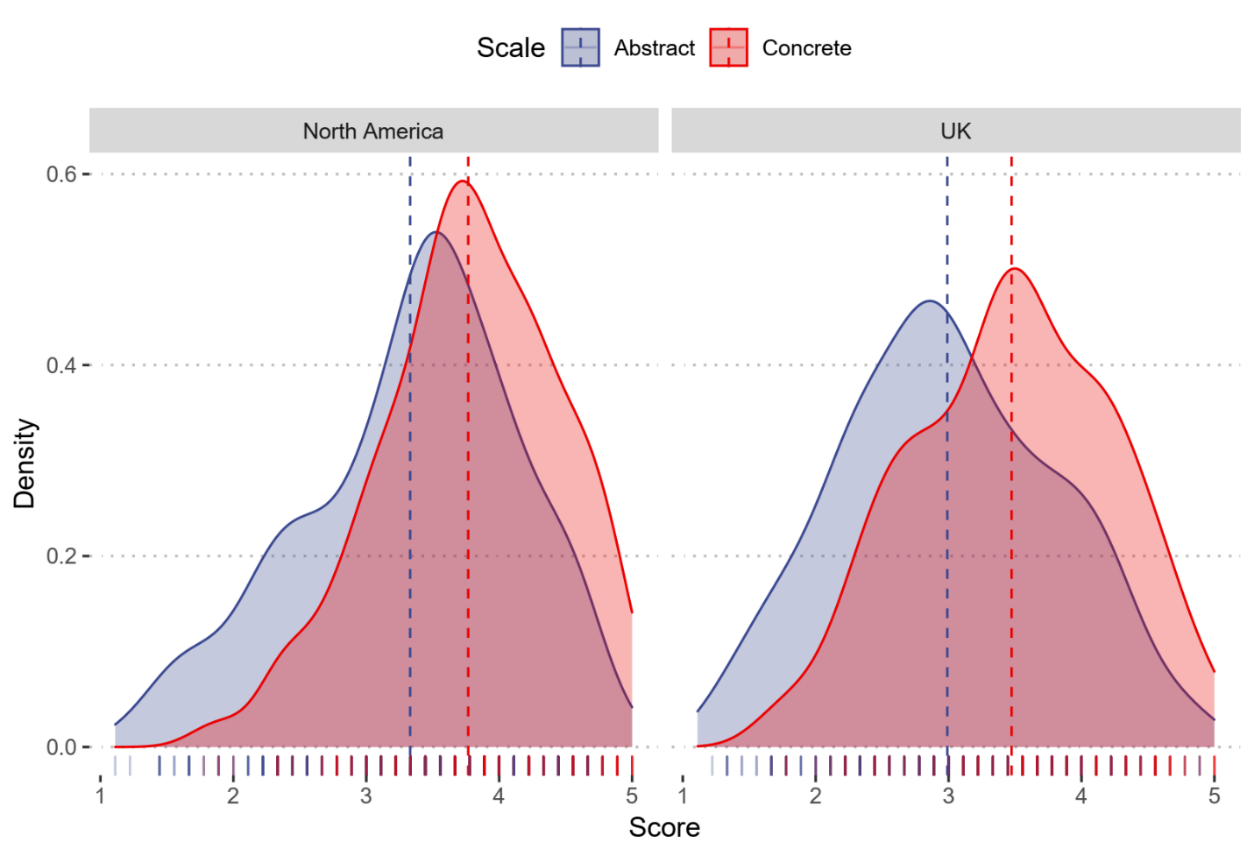


Figure S5. Distribution of abstract and concrete construal

Average scores across 9 items of each sub-scale across individuals reflecting on interpersonal challenges in Study 2b, along with average tendencies (dotted lines).

## Table S9: EFA in Study 1a, Round 1

|  | Factor |  |  |  |
| --- | --- | --- | --- | --- |
| Item# | 1 | 2 | 4 | 3 |
| abstract 1 |  |  | .387 |  |
| abstract 2 |  |  | .471 |  |
| abstract 3 |  |  | .620 |  |
| abstract 4 |  |  | .767 |  |
| abstract 5 |  |  | .541 |  |
| abstract 6 | .308 |  | .366 |  |
| abstract 7 |  |  | .544 |  |
| abstract 8 |  |  | .471 |  |
| abstract 9 |  | .34 | .390 |  |
| abstract 10 |  | .507 |  |  |
| abstract 11 | .487 |  |  |  |
| abstract 12 |  | .500 |  |  |
| abstract 13 | .421 |  | .308 |  |
| abstract 14 |  | .503 | .310 |  |
| abstract 15 |  | .592 |  |  |
| abstract 17 |  | .700 |  |  |
| abstract 18 |  |  |  |  |
| abstract 19 | .466 |  |  |  |
| abstract 21 | .437 |  |  |  |
| abstract 22 |  |  |  |  |
| abstract 24 | .625 |  |  |  |
| abstract 25 |  | .525 |  |  |
| abstract 26 | .378 |  |  |  |
| abstract 27 | .594 |  |  |  |
| abstract 28 |  | .390 |  |  |
| abstract 30 | .467 |  | .39 |  |
| abstract 31 |  |  | .323 |  |
| abstract 32 | .580 | .308 |  |  |
| abstract 33 | .587 |  |  |  |
| abstract 34 | .546 |  |  |  |
| abstract 35 | .474 |  |  |  |
| concrete 1 | .597 |  |  |  |
| concrete 2 |  | .619 |  |  |
| concrete 3 | .633 |  |  |  |
| concrete 4 | .625 |  |  |  |
| concrete 5 | .411 |  |  | .324 |
| concrete 6 | .504 |  |  |  |
| concrete 7 | .364 |  |  |  |
| concrete 8 | .613 |  |  |  |
| concrete 9 |  | .395 |  |  |
| concrete 10 | .615 |  |  |  |
| concrete 11 |  |  |  | .351 |
| concrete 12 | .481 |  |  |  |
| concrete 13 |  | .333 |  |  |
| concrete 14 | .519 |  |  |  |
| concrete 15 | .312 |  |  |  |
| concrete 16 | .524 |  |  |  |
| concrete 17 |  |  |  | .502 |
| concrete 18 | .439 |  |  |  |
| concrete 19 |  | .439 |  |  |
| concrete 21 |  | .641 |  |  |
| concrete 22 | .471 |  |  |  |
| concrete 24 | .340 |  |  |  |
| concrete 25 |  | .627 |  |  |
| concrete 26 | .525 |  |  |  |
| concrete 27 |  |  |  | .349 |
| concrete 28 | .605 |  |  |  |
| concrete 29 |  |  |  | .378 |
| concrete 30 |  |  |  | .522 |
| concrete 31 |  |  |  | .619 |
| concrete 32 |  |  |  | .602 |
| concrete 33 |  | .613 |  |  |
| concrete 34 |  | .423 |  |  |

*Note.* Highlights indicate items that were removed in the next round.

## Table S10: EFA in Study 1a, Round 2

|  | Factor |  |  |  |
| --- | --- | --- | --- | --- |
| Item# | 1 | 2 | 4 | 3 |
| abstract 1 |  |  | .424 |  |
| abstract 2 |  |  | .483 |  |
| abstract 3 |  |  | .616 |  |
| abstract 4 |  |  | .771 |  |
| abstract 5 |  |  | .510 |  |
| abstract 7 |  |  | .505 |  |
| abstract 8 |  |  | .486 |  |
| abstract 10 |  | .485 |  |  |
| abstract 11 | .536 |  |  |  |
| abstract 12 |  | .468 |  |  |
| abstract 15 |  | .623 |  |  |
| abstract 17 |  | .713 |  |  |
| abstract 19 | .520 |  |  |  |
| abstract 21 | .469 |  |  |  |
| abstract 24 | .662 |  |  |  |
| abstract 25 |  | .506 |  |  |
| abstract 26 |  |  |  |  |
| abstract 27 | .559 |  |  |  |
| abstract 28 |  | .359 |  |  |
| abstract 31 |  |  | .330 |  |
| abstract 33 | .525 |  |  |  |
| abstract 34 | .530 |  |  |  |
| abstract 35 | .372 |  |  |  |
| concrete 1 | .597 |  |  |  |
| concrete 2 |  | .634 |  |  |
| concrete 3 | .691 |  |  |  |
| concrete 4 | .653 |  |  |  |
| concrete 6 | .530 |  |  |  |
| concrete 7 | .392 |  |  |  |
| concrete 8 | .597 |  |  |  |
| concrete 9 |  | .365 |  |  |
| concrete 10 | .618 |  |  |  |
| concrete 11 |  |  |  | .377 |
| concrete 12 | .484 |  |  |  |
| concrete 13 |  |  |  |  |
| concrete 14 | .537 |  |  |  |
| concrete 15 | .324 |  |  |  |
| concrete 16 | .577 |  |  |  |
| concrete 17 |  |  |  | .442 |
| concrete 18 | .470 |  |  |  |
| concrete 19 |  | .422 |  |  |
| concrete 21 |  | .669 |  |  |
| concrete 22 | .473 |  |  |  |
| concrete 24 | .395 |  |  |  |
| concrete 25 |  | .631 |  |  |
| concrete 26 | .528 |  |  |  |
| concrete 27 | .332 |  |  | .338 |
| concrete 28 | .637 |  |  |  |
| concrete 29 | .303 |  |  | .361 |
| concrete 30 |  |  |  | .563 |
| concrete 31 |  |  |  | .675 |
| concrete 32 |  |  |  | .628 |
| concrete 33 |  | .576 |  |  |
| concrete 34 |  | .409 |  |  |

*Note.* Highlights indicate items that were removed in the next round.

## Table S11: EFA in Study 1a, Round 3

|  | Factor |  |  |  |
| --- | --- | --- | --- | --- |
| Item# | 1 | 2 | 4 | 3 |
| abstract 1 |  |  | .420 |  |
| abstract 2 |  |  | .486 | .320 |
| abstract 3 |  |  | .639 |  |
| abstract 4 |  |  | .767 |  |
| abstract 5 |  |  | .518 |  |
| abstract 7 | .309 |  | .520 |  |
| abstract 8 |  |  | .478 |  |
| abstract 10 |  | .485 |  |  |
| abstract 11 | .550 |  |  |  |
| abstract 12 |  | .470 |  |  |
| abstract 15 |  | .643 |  |  |
| abstract 17 |  | .726 |  |  |
| abstract 19 | .525 |  |  |  |
| abstract 21 | .472 |  |  |  |
| abstract 24 | .661 |  |  |  |
| abstract 25 |  | .487 |  |  |
| abstract 26 |  |  |  |  |
| abstract 27 | .546 |  |  |  |
| abstract 28 |  | .345 |  |  |
| abstract 31 |  |  | .343 |  |
| abstract 33 | .508 |  |  |  |
| abstract 34 | .520 |  |  |  |
| abstract 35 | .356 |  |  |  |
| concrete 1 | .591 |  |  |  |
| concrete 2 |  | .640 |  |  |
| concrete 3 | .697 |  |  |  |
| concrete 4 | .660 |  |  |  |
| concrete 6 | .542 |  |  |  |
| concrete 7 | .398 |  |  |  |
| concrete 8 | .599 |  |  |  |
| concrete 9 |  | .344 |  |  |
| concrete 10 | .612 |  |  |  |
| concrete 11 |  |  |  |  |
| concrete 12 | .491 |  |  |  |
| concrete 14 | .543 |  |  |  |
| concrete 15 | .337 |  |  |  |
| concrete 16 | .578 |  |  |  |
| concrete 17 |  |  |  | .447 |
| concrete 18 | .488 |  |  |  |
| concrete 19 |  | .425 |  |  |
| concrete 21 |  | .662 |  |  |
| concrete 22 | .470 |  |  |  |
| concrete 24 | .392 |  |  |  |
| concrete 25 |  | .623 |  |  |
| concrete 26 | .534 |  |  |  |
| concrete 28 | .636 |  |  |  |
| concrete 30 |  |  |  | .610 |
| concrete 31 |  |  |  | .696 |
| concrete 32 |  |  |  | .606 |
| concrete 33 |  | .567 |  |  |
| concrete 34 |  | .409 |  |  |

*Note.* Highlights indicate items that were removed in the next round.

## Table S12: EFA in Study 2a, Round 4

|  | Factor |  |  |  |
| --- | --- | --- | --- | --- |
| Item# | 1 | 2 | 4 | 3 |
| abstract 1 |  |  | .427 |  |
| abstract 3 |  |  | .654 |  |
| abstract 4 |  |  | .700 |  |
| abstract 5 |  |  | .495 |  |
| abstract 8 |  |  | .512 |  |
| abstract 10 |  | .495 |  |  |
| abstract 11 | .558 |  |  |  |
| abstract 12 |  | .478 |  |  |
| abstract 15 |  | .642 |  |  |
| abstract 17 |  | .718 |  |  |
| abstract 19 | .519 |  |  |  |
| abstract 21 | .467 |  |  |  |
| abstract 24 | .673 |  |  |  |
| abstract 25 |  | .493 |  |  |
| abstract 27 | .554 |  |  |  |
| abstract 28 |  | .346 |  |  |
| abstract 31 |  |  | .346 |  |
| abstract 33 | .537 |  |  |  |
| abstract 34 | .521 |  |  |  |
| abstract 35 | .375 |  |  |  |
| concrete 1 | .595 |  |  |  |
| concrete 2 |  | .633 |  |  |
| concrete 3 | .703 |  |  |  |
| concrete 4 | .663 |  |  |  |
| concrete 6 | .543 |  |  |  |
| concrete 7 | .418 |  |  |  |
| concrete 8 | .595 |  |  |  |
| concrete 9 |  | .342 |  |  |
| concrete 10 | .598 |  |  |  |
| concrete 12 | .487 |  |  |  |
| concrete 14 | .562 |  |  |  |
| concrete 15 | .339 |  |  |  |
| concrete 16 | .584 |  |  |  |
| concrete 17 |  |  |  | .446 |
| concrete 18 | .494 |  |  |  |
| concrete 19 |  | .430 |  |  |
| concrete 21 |  | .651 |  |  |
| concrete 22 | .468 |  |  |  |
| concrete 24 | .377 |  |  |  |
| concrete 25 |  | .623 |  |  |
| concrete 26 | .512 |  |  |  |
| concrete 28 | .633 |  |  |  |
| concrete 30 |  |  |  | .613 |
| concrete 31 |  |  |  | .731 |
| concrete 32 |  |  |  | .602 |
| concrete 33 |  | .557 |  |  |
| concrete 34 |  | .410 |  |  |

*Note.* Highlights indicate items that were removed in the next round (leaving 10 abstract and 10 concrete items). Items are selected based on loading on unique factors or highest loading on factors 1 (for concrete) and 2 (or abstract).

## Table S13: EFA in Study 1a, Round 5

|  | Factor |  |  |  |
| --- | --- | --- | --- | --- |
|  | 1 | 4 | 3 | 2 |
| abstract 1 |  | .429 |  |  |
| abstract 3 |  | .708 |  |  |
| abstract 4 |  | .787 |  |  |
| abstract 5 |  | .511 |  |  |
| abstract 8 |  | .609 |  |  |
| abstract 10 |  |  |  | .377 |
| abstract 15 |  |  |  | .657 |
| abstract 31 |  | .360 |  |  |
| abstract 17 |  |  |  | .791 |
| abstract 25 |  |  |  | .400 |
| concrete 1 | .635 |  |  |  |
| concrete 3 | .648 |  |  |  |
| concrete 4 | .660 |  |  |  |
| concrete 28 | .613 |  |  |  |
| concrete 8 | .643 |  |  |  |
| concrete 16 | .509 |  |  |  |
| concrete 30 |  |  | .721 |  |
| concrete 31 |  |  | .785 |  |
| concrete 32 |  |  | .565 |  |
| concrete 17 |  |  | .365 |  |

*Note.* Highlights indicate psychological distance and emotion/intuition items that were removed in the next round based on expert recommendations

## Table S14: Modification indices for a 2-factor model of construal in Study 1a

*Modification indices for the 2-factor model of abstract and concrete construal in the exploratory Study 1a.*

| Error terms | | | *M.I.* | Change |
| --- | --- | --- | --- | --- |
| abstract 15 | ~~ | abstract 17 | 74.952 | .732 |
| abstract 12 | ~~ | abstract 15 | 3.905 | .409 |
| abstract 12 | ~~ | abstract 17 | 21.994 | .372 |
| concrete 12 | ~~ | concrete 16 | 13.754 | .164 |
| abstract 3 | ~~ | concrete 22 | 13.261 | -.183 |
| abstract 26 | ~~ | concrete 22 | 13.161 | .187 |
| abstract 3 | ~~ | abstract 4 | 12.326 | .196 |
| concrete | =~ | abstract 31 | 11.62 | .539 |
| abstract 1 | ~~ | abstract 28 | 11.587 | .213 |
| concrete 12 | ~~ | concrete 14 | 1.886 | .154 |
| abstract 5 | ~~ | concrete 4 | 1.302 | .140 |
| abstract 12 | ~~ | abstract 28 | 1.257 | .225 |
| concrete | =~ | abstract 4 | 1.203 | -.513 |
| concrete | =~ | abstract 17 | 9.925 | -.641 |
| concrete 12 | ~~ | concrete 28 | 9.753 | -.129 |
| abstract 31 | ~~ | concrete 10 | 9.732 | .154 |
| abstract 5 | ~~ | concrete 22 | 9.726 | -.147 |
| abstract 15 | ~~ | abstract 31 | 9.668 | -.203 |
| concrete 1 | ~~ | concrete 27 | 8.761 | -.140 |
| abstract 8 | ~~ | abstract 31 | 8.240 | .148 |
| abstract 5 | ~~ | abstract 15 | 7.755 | -.172 |
| abstract 8 | ~~ | concrete 22 | 6.787 | .123 |
| abstract 17 | ~~ | abstract 31 | 6.783 | -.183 |
| concrete | =~ | abstract 26 | 6.766 | .423 |
| abstract 17 | ~~ | concrete 3 | 6.672 | -.160 |
| abstract 8 | ~~ | abstract 15 | 6.379 | -.156 |
| abstract 12 | ~~ | concrete 14 | 6.095 | .139 |
| concrete | =~ | abstract 5 | 5.895 | .369 |
| abstract 3 | ~~ | abstract 12 | 5.604 | -.147 |
| abstract 28 | ~~ | concrete 22 | 5.447 | .135 |
| abstract 3 | ~~ | abstract 26 | 5.382 | -.132 |
| abstract 31 | ~~ | concrete 27 | 5.278 | .115 |
| abstract | =~ | concrete 14 | 5.272 | -.305 |
| abstract 8 | ~~ | concrete 4 | 5.217 | -.100 |
| concrete 4 | ~~ | concrete 28 | 5.193 | .089 |
| abstract 12 | ~~ | concrete 27 | 5.020 | .127 |

*Note*. Highlights show items with high modification indices.

## Table S15: EFA in Study 1b, Round 1

|  | Factor |  |
| --- | --- | --- |
|  | 1 | 2 |
| abstract 1 | .465 |  |
| abstract 3 | .561 |  |
| abstract 4 | .669 |  |
| abstract 5 | .549 |  |
| abstract 8 | .677 |  |
| abstract 12 | .560 |  |
| abstract 15 | .626 |  |
| abstract 17 | .611 |  |
| abstract 26 | .501 |  |
| abstract 28 | .522 |  |
| abstract 31 | .387 | .344 |
| concrete 1 |  | .567 |
| concrete 3 |  | .496 |
| concrete 4 |  | .367 |
| concrete 8 |  | .700 |
| concrete 10 |  | .529 |
| concrete 12 |  | .601 |
| concrete 14 |  | .525 |
| concrete 16 |  | .491 |
| concrete 22 |  | .610 |
| concrete 27 |  | .480 |
| concrete 28 |  | .559 |

*Note.* Highlights indicate items that were removed in the next round, either due to cross-loading, semantic similarity to another item (removing abstract 12 in favor of higher loading abstract 15) or poor loading (concrete 4).

## Table S16: EFA in Study 1b, Round 2

|  | Factor |  |
| --- | --- | --- |
|  | 2 | 1 |
| abstract 1 |  | .489 |
| abstract 3 |  | .586 |
| abstract 4 |  | .734 |
| abstract 5 |  | .566 |
| abstract 8 |  | .733 |
| abstract 15 |  | .554 |
| abstract 17 |  | .540 |
| abstract 26 |  | .500 |
| abstract 28 |  | .509 |
| concrete 1 | .586 |  |
| concrete 3 | .475 |  |
| concrete 8 | .702 |  |
| concrete 10 | .496 |  |
| concrete 12 | .586 |  |
| concrete 14 | .529 |  |
| concrete 16 | .527 |  |
| concrete 22 | .625 |  |
| concrete 27 | .515 |  |
| concrete 28 | .519 |  |

*Note.* Highlights indicate an item that was removed in the next round due to poor loading and to ensure balanced number of items per factor.

## Table S17: Factor Loadings in Study 1b, Final Round 4

*Items and factor loadings in the final (round 3) EFA in Study 1b*

|  | Factor |  |
| --- | --- | --- |
|  | 1 | 2 |
| I thought about what the situation says about me as a person. (abstract 1) | .490 |  |
| I thought about the life experiences that led to this situation. (abstract 3) | .594 |  |
| I thought about how this situation fits into the broader context of my life. (abstract 4) | .742 |  |
| I thought about how this situation impacts my overall well-being. (abstract 5) | .574 |  |
| I focused on the broader meaning of the situation. (abstract 8) | .737 |  |
| I thought about the situation as one of many similar experiences. (abstract 15) | .539 |  |
| It seemed to represent a typical event in my life. (abstract 17) | .525 |  |
| I focused on the big picture. (abstract 26) | .507 |  |
| I asked myself why this situation made me feel the way it did. (abstract 28) | .506 |  |
| I considered the specific words I was saying in the situation. (concrete 1) |  | .596 |
| I focused on the specific details of the situation. (concrete 8) |  | .690 |
| I considered the concrete and immediate outcomes of the situation. (concrete 10) |  | .454 |
| I focused on each part of the situation as the experience unfolded. (concrete 12) |  | .579 |
| I focused on the “here and now” of the situation. (concrete 14) |  | .513 |
| I focused on specific aspects of the experience. (concrete 16) |  | .540 |
| I focused on what I was saying and doing in the situation. (concrete 22) |  | .660 |
| I focused on specific behaviors during the situation. (concrete 27) |  | .542 |
| I considered the specific circumstances surrounding the situation. (concrete 28) |  | .485 |

## Table S18: Parameter Estimates of the Bifactor Model in Study 1b – North America

*Parameter estimates in the final bifactor model in Study 1b – North American Sample*

| Parameter | Coefficient | CI_low | CI_high | *z* | *p* | Component |
| --- | --- | --- | --- | --- | --- | --- |
| abstract =~ abstract_1 | 1.000 | 1.000 | 1.000 |  | < .001 | Loading |
| abstract =~ abstract_3 | 1.146 | 0.779 | 1.514 | 6.111 | < .001 | Loading |
| abstract =~ abstract_4 | 1.127 | 0.706 | 1.548 | 5.245 | < .001 | Loading |
| abstract =~ abstract_5 | 1.065 | 0.708 | 1.422 | 5.853 | < .001 | Loading |
| abstract =~ abstract_8 | 1.319 | 0.939 | 1.700 | 6.794 | < .001 | Loading |
| abstract =~ abstract_15 | 1.925 | 0.543 | 3.306 | 2.731 | .006 | Loading |
| abstract =~ abstract_17 | 1.715 | 0.637 | 2.793 | 3.117 | .002 | Loading |
| abstract =~ abstract_26 | 0.941 | 0.577 | 1.305 | 5.064 | < .001 | Loading |
| abstract =~ abstract_28 | 1.110 | 0.782 | 1.438 | 6.630 | < .001 | Loading |
| concrete =~ concrete_1 | 1.000 | 1.000 | 1.000 |  | < .001 | Loading |
| concrete =~ concrete_8 | 0.953 | 0.698 | 1.208 | 7.315 | < .001 | Loading |
| concrete =~ concrete_10 | 0.741 | 0.482 | 0.999 | 5.617 | < .001 | Loading |
| concrete =~ concrete_12 | 0.962 | 0.701 | 1.224 | 7.219 | < .001 | Loading |
| concrete =~ concrete_14 | 0.761 | 0.497 | 1.026 | 5.644 | < .001 | Loading |
| concrete =~ concrete_16 | 0.962 | 0.697 | 1.227 | 7.106 | < .001 | Loading |
| concrete =~ concrete_22 | 0.901 | 0.678 | 1.123 | 7.947 | < .001 | Loading |
| concrete =~ concrete_27 | 1.119 | 0.822 | 1.417 | 7.378 | < .001 | Loading |
| concrete =~ concrete_28 | 0.757 | 0.524 | 0.990 | 6.359 | < .001 | Loading |
| method =~ abstract_1 | 1.000 | 1.000 | 1.000 |  | < .001 | Loading |
| method =~ abstract_3 | 1.224 | 0.635 | 1.813 | 4.075 | < .001 | Loading |
| method =~ abstract_4 | 1.721 | 0.864 | 2.577 | 3.937 | < .001 | Loading |
| method =~ abstract_5 | 1.397 | 0.727 | 2.066 | 4.089 | < .001 | Loading |
| method =~ abstract_8 | 1.529 | 0.856 | 2.201 | 4.455 | < .001 | Loading |
| method =~ abstract_15 | -0.754 | -2.778 | 1.270 | -0.730 | .465 | Loading |
| method =~ abstract_17 | -0.805 | -3.086 | 1.475 | -0.692 | .489 | Loading |
| method =~ abstract_26 | 1.358 | 0.655 | 2.060 | 3.786 | < .001 | Loading |
| method =~ abstract_28 | 0.859 | 0.351 | 1.367 | 3.316 | .001 | Loading |
| method =~ concrete_1 | 0.573 | 0.118 | 1.028 | 2.467 | .014 | Loading |
| method =~ concrete_8 | 0.538 | 0.071 | 1.004 | 2.258 | .024 | Loading |
| method =~ concrete_10 | 1.143 | 0.301 | 1.986 | 2.659 | .008 | Loading |
| method =~ concrete_12 | 0.848 | 0.247 | 1.448 | 2.768 | .006 | Loading |
| method =~ concrete_14 | 0.293 | -0.132 | 0.718 | 1.351 | .177 | Loading |
| method =~ concrete_16 | 0.322 | -0.086 | 0.730 | 1.547 | .122 | Loading |
| method =~ concrete_22 | 0.398 | -0.034 | 0.830 | 1.806 | .071 | Loading |
| method =~ concrete_27 | 0.217 | -0.380 | 0.815 | 0.713 | .476 | Loading |
| method =~ concrete_28 | 0.789 | 0.167 | 1.411 | 2.487 | .013 | Loading |
| concrete_1 ~~ concrete_22 | 0.219 | 0.117 | 0.322 | 4.215 | < .001 | Covariance |
| abstract_1 ~~ abstract_28 | 0.313 | 0.167 | 0.460 | 4.188 | < .001 | Covariance |
| concrete ~~ method | 0.000 | 0.000 | 0.000 |  | < .001 | Covariance |
| abstract ~~ method | 0.000 | 0.000 | 0.000 |  | < .001 | Covariance |
| abstract ~~ concrete | 0.127 | -0.011 | 0.266 | 1.800 | .072 | Covariance |

## Table S19: Parameter Estimates of the Bifactor Model in Study 1b – UK

*Parameter estimates in the final bifactor model in Study 1b – UK Sample*

|  | Parameter | | Coefficient | CI_low | CI_high | | *z* | | *p* | Component |  |
| --- | --- | --- | --- | --- | --- | --- | --- | --- | --- | --- | --- |
|  | abstract =~ abstract_1 | | 1.000 | 1.000 | 1.000 | |  | | < .001 | Loading |  |
|  | abstract =~ abstract_3 | | 1.146 | 0.779 | 1.514 | | 6.111 | | < .002 | Loading |  |
|  | abstract =~ abstract_4 | | 1.127 | 0.706 | 1.548 | | 5.245 | | < .003 | Loading |  |
|  | abstract =~ abstract_5 | | 1.065 | 0.708 | 1.422 | | 5.853 | | < .004 | Loading |  |
|  | abstract =~ abstract_8 | | 1.319 | 0.939 | 1.700 | | 6.794 | | < .005 | Loading |  |
|  | abstract =~ abstract_15 | | 1.925 | 0.543 | 3.306 | | 2.731 | | .006 | Loading |  |
|  | abstract =~ abstract_17 | | 1.715 | 0.637 | 2.793 | | 3.117 | | .002 | Loading |  |
|  | abstract =~ abstract_26 | | 0.941 | 0.577 | 1.305 | | 5.064 | | < .001 | Loading |  |
|  | abstract =~ abstract_28 | | 1.110 | 0.782 | 1.438 | | 6.630 | | < .001 | Loading |  |
|  | concrete =~ concrete_1 | | 1.000 | 1.000 | 1.000 | |  | | < .001 | Loading |  |
|  | concrete =~ concrete_8 | | 0.953 | 0.698 | 1.208 | | 7.315 | | < .001 | Loading |  |
|  | concrete =~ concrete_10 | | 0.741 | 0.482 | 0.999 | | 5.617 | | < .001 | Loading |  |
|  | concrete =~ concrete_12 | | 0.962 | 0.701 | 1.224 | | 7.219 | | < .001 | Loading |  |
|  | concrete =~ concrete_14 | | 0.761 | 0.497 | 1.026 | | 5.644 | | < .001 | Loading |  |
|  | concrete =~ concrete_16 | | 0.962 | 0.697 | 1.227 | | 7.106 | | < .001 | Loading |  |
|  | concrete =~ concrete_22 | | 0.901 | 0.678 | 1.123 | | 7.947 | | < .001 | Loading |  |
|  | concrete =~ concrete_27 | | 1.119 | 0.822 | 1.417 | | 7.378 | | < .001 | Loading |  |
|  | concrete =~ concrete_28 | | 0.757 | 0.524 | 0.990 | | 6.359 | | < .001 | Loading |  |
|  | method =~ abstract_1 | | 1.000 | 1.000 | 1.000 | |  | | < .001 | Loading |  |
|  | method =~ abstract_3 | | 1.224 | 0.635 | 1.813 | | 4.075 | | < .001 | Loading |  |
|  | method =~ abstract_4 | | 1.721 | 0.864 | 2.577 | | 3.937 | | < .001 | Loading |  |
|  | method =~ abstract_5 | | 1.397 | 0.727 | 2.066 | | 4.089 | | < .001 | Loading |  |
|  | method =~ abstract_8 | | 1.529 | 0.856 | 2.201 | | 4.455 | | < .001 | Loading |  |
|  | method =~ abstract_15 | | -0.754 | -2.778 | 1.270 | | -0.730 | | .465 | Loading |  |
|  | method =~ abstract_17 | | -0.805 | -3.086 | 1.475 | | -0.692 | | .489 | Loading |  |
|  | method =~ abstract_26 | | 1.358 | 0.655 | 2.060 | | 3.786 | | < .001 | Loading |  |
|  | method =~ abstract_28 | | 0.859 | 0.351 | 1.367 | | 3.316 | | .001 | Loading |  |
|  | method =~ concrete_1 | | 0.573 | 0.118 | 1.028 | | 2.467 | | .014 | Loading |  |
|  | method =~ concrete_8 | | 0.538 | 0.071 | 1.004 | | 2.258 | | .024 | Loading |  |
|  | method =~ concrete_10 | | 1.143 | 0.301 | 1.986 | | 2.659 | | .008 | Loading |  |
|  | method =~ concrete_12 | | 0.848 | 0.247 | 1.448 | | 2.768 | | .006 | Loading |  |
|  | method =~ concrete_14 | | 0.293 | -0.132 | 0.718 | | 1.351 | | .177 | Loading |  |
|  | method =~ concrete_16 | | 0.322 | -0.086 | 0.730 | | 1.547 | | .122 | Loading |  |
|  | method =~ concrete_22 | | 0.398 | -0.034 | 0.830 | | 1.806 | | .071 | Loading |  |
|  | method =~ concrete_27 | | 0.217 | -0.380 | 0.815 | | 0.713 | | .476 | Loading |  |
|  | method =~ concrete_28 | | 0.789 | 0.167 | 1.411 | | 2.487 | | .013 | Loading |  |
|  | concrete_1 ~~ concrete_22 | | 0.219 | 0.117 | 0.322 | | 4.215 | | < .001 | Covariance |  |
|  | abstract_1 ~~ abstract_28 | | 0.313 | 0.167 | 0.460 | | 4.188 | | < .001 | Covariance |  |
|  | concrete ~~ method | | 0.000 | 0.000 | 0.000 | |  | | < .001 | Covariance |  |
|  | abstract ~~ method | | 0.000 | 0.000 | 0.000 | |  | | < .001 | Covariance |  |
|  | abstract ~~ concrete | | 0.127 | -0.011 | 0.266 | | 1.800 | | .072 | Covariance |  |
|  | |  | | | |  | |  | | | |

## Table S20: Construal and Covariates Predicting Wisdom in Study 1b

*Results of multiple regressions of abstract and concrete construal predicting wisdom when including trait-level, situation-specific, and deliberation-time-related covariates*

|  | Version A: Construal factor scores as predictors | | | | | |  | | Version B: Construal item average scores as predictors | | | | | | | | | |  |
| --- | --- | --- | --- | --- | --- | --- | --- | --- | --- | --- | --- | --- | --- | --- | --- | --- | --- | --- | --- |
|  |  | | *Est.* | *S.E.* | *t val.* | *p* | |  | | *Est.* | | | *S.E.* | | *t val.* | | *p* | | |
| Model 1  (trait-level covariates)  *F*(15,544) = 17.073  *p* < .001  *R*² = 0.320  Adj. *R*² = 0.301 | (Intercept) | 3.103 | | 0.033 | 93.575 | < .001 | | Model 1  (trait-level covariates)  *F*(15,544) = 26.871  *p* < .001  *R*² = 0.426  Adj. *R*² = 0.410 | | | 3.099 | 0.031 | | 98.727 | | < .001 | |  |  |
|  | **abstract** | **0.506** | | **0.089** | **5.676** | **< .001** | |  |  |  | **0.345** | **0.041** | | **8.499** | | **< .001** | |  |  |
|  | **concrete** | **0.580** | | **0.100** | **5.829** | **< .001** | |  |  |  | **0.386** | **0.048** | | **7.990** | | **< .001** | |  |  |
|  | **abstract X concrete** | **0.479** | | **0.214** | **2.239** | **.026** | |  |  |  | **0.107** | **0.048** | | **2.246** | | **.025** | |  |  |
|  | REI_Analytic | 0.109 | | 0.060 | 1.804 | .072 | |  |  |  | 0.060 | 0.056 | | 1.069 | | .286 | |  |  |
|  | REI_Imagination | 0.059 | | 0.071 | 0.836 | .404 | |  |  |  | -0.044 | 0.066 | | -0.662 | | .509 | |  |  |
|  | REI_Emotionality | 0.089 | | 0.077 | 1.165 | .245 | |  |  |  | 0.072 | 0.071 | | 1.014 | | .311 | |  |  |
|  | REI_Intuition | 0.044 | | 0.068 | 0.651 | .515 | |  |  |  | 0.023 | 0.062 | | 0.363 | | .717 | |  |  |
|  | CRT | -0.051 | | 0.028 | -1.815 | .070 | |  |  |  | -0.040 | 0.026 | | -1.536 | | .125 | |  |  |
|  | BIF | -0.001 | | 0.141 | -0.004 | .997 | |  |  |  | -0.039 | 0.130 | | -0.304 | | .761 | |  |  |
|  | Hexaco_HonHum | -0.035 | | 0.050 | -0.706 | .481 | |  |  |  | -0.020 | 0.046 | | -0.441 | | .659 | |  |  |
|  | Hexaco_Emotion | -0.062 | | 0.060 | -1.034 | .302 | |  |  |  | -0.062 | 0.055 | | -1.129 | | .259 | |  |  |
|  | Hexaco_Extra | 0.127 | | 0.049 | 2.572 | .010 | |  |  |  | 0.109 | 0.046 | | 2.399 | | .017 | |  |  |
|  | Hexaco_Agree | 0.358 | | 0.052 | 6.932 | < .001 | |  |  |  | 0.270 | 0.048 | | 5.647 | | < .001 | |  |  |
|  | Hexaco_Cons | -0.192 | | 0.059 | -3.241 | .001 | |  |  |  | -0.200 | 0.055 | | -3.673 | | < .001 | |  |  |
|  | Hexaco_Open | -0.016 | | 0.062 | -0.253 | .800 | |  |  |  | 0.040 | 0.057 | | 0.707 | | .480 | |  |  |
| Model 2 (state-level covariates)  *F*(8,551) = 39.260  *p* < .001  *R*² = 0.363  Adj. *R*² = 0.354 | (Intercept) | 3.102 | | 0.032 | 97.342 | < .001 | | Model 2  (state-level covariates)  *F*(8,551) = 54.575  *p* < .001  *R*² = 0.442  Adj. *R*² = 0.434 | | | 3.096 | 0.031 | | 100.870 | | < .001 | |  |  |
|  | **abstract** | **0.290** | | **0.092** | **3.160** | **.002** | |  |  |  | **0.293** | **0.044** | | **6.654** | | **< .001** | |  |  |
|  | **concrete** | **0.541** | | **0.095** | **5.727** | **< .001** | |  |  |  | **0.382** | **0.048** | | **8.015** | | **< .001** | |  |  |
|  | **abstract X concrete** | **0.508** | | **0.204** | **2.490** | **.013** | |  |  |  | **0.118** | **0.046** | | **2.548** | | **.011** | |  |  |
|  | Psychological distance | 0.249 | | 0.030 | 8.396 | < .001 | |  |  |  | 0.179 | 0.029 | | 6.190 | | < .001 | |  |  |
|  | Temporal closeness | 0.042 | | 0.028 | 1.457 | .146 | |  |  |  | -0.016 | 0.027 | | -0.573 | | .567 | |  |  |
|  | Closeness to conflict person | 0.111 | | 0.020 | 5.438 | < .001 | |  |  |  | 0.112 | 0.019 | | 5.885 | | < .001 | |  |  |
|  | Reliance on emotion/intuition | 0.063 | | 0.042 | 1.500 | .134 | |  |  |  | -0.049 | 0.041 | | -1.184 | | .237 | |  |  |
|  | Impulsivity assessment | -0.168 | | 0.027 | -6.207 | < .001 | |  |  |  | -0.110 | 0.026 | | -4.303 | | < .001 | |  |  |
| Model 3  (deliberation time)  *F*(5,554) = 26.806  *p* < .001  *R*² = .195  Adj. *R*² = .188 | (Intercept) | 3.097 | | 0.036 | 86.663 | < .001 | | Model 3  (deliberation time)  *F*(5,554) = 59.671  *p* < .001  *R*² = .350  Adj. *R*² = .344 | | | 3.097 | 0.033 | | 93.703 | | < .001 | |  |  |
|  | **abstract** | **0.498** | | **0.093** | **5.372** | **< .001** | |  |  |  | **0.371** | **0.041** | | **8.984** | | **< .001** | |  |  |
|  | **concrete** | **0.714** | | **0.102** | **6.990** | **< .001** | |  |  |  | **0.426** | **0.048** | | **8.908** | | **< .001** | |  |  |
|  | **abstract X concrete** | **0.604** | | **0.229** | **2.636** | **.009** | |  |  |  | **0.116** | **0.050** | | **2.337** | | **.020** | |  |  |
|  | *log* (Time_Reflect) | -0.016 | | 0.032 | -0.513 | .608 | |  |  |  | -0.031 | 0.028 | | -1.086 | | .278 | |  |  |
|  | *log* (Study Duration in sec) | -0.052 | | 0.082 | -0.635 | .526 | |  |  |  | -0.011 | 0.073 | | -0.148 | | .882 | |  |  |

*Note.* All predictors are mean-centered. Version B uses average of respective construal level items as indices of abstractness and concreteness.

## Table S21: Sample-wise Nomological Net of Abstract and Concrete Construal in Study 1b

*Sample-wise zero-order correlations between abstract and concrete construal and aspects of nomological network in Study 1b.*

|  |  | Abstract | | | | | | | Concrete | | | | | | |
| --- | --- | --- | --- | --- | --- | --- | --- | --- | --- | --- | --- | --- | --- | --- | --- |
| Group | Covariate | r |  | *95% CI* | *t* | *df* | *p* | *r* | |  | *95% CI* | *t* | *df* | *p* |  |
| North America | REI_Analytic | -0.27 | [-0.37, | -0.17] | -5.04 | 320 | < .001*** | 0.08 | | [-0.03, | 0.19] | 1.47 | 320 | > .999 |  |
|  | REI_Intuition | 0.19 | [ 0.08, | 0.29] | 3.41 | 320 | .006** | 0.21 | | [ 0.10, | 0.31] | 3.83 | 320 | .002** |  |
|  | REI_Imagination | 0.15 | [ 0.04, | 0.25] | 2.66 | 320 | .058 | 0.29 | | [ 0.19, | 0.39] | 5.5 | 320 | < .001*** |  |
|  | REI_Emotionality | 0.13 | [ 0.02, | 0.23] | 2.31 | 320 | .108 | 0.22 | | [ 0.11, | 0.32] | 3.97 | 320 | .001** |  |
|  | CRT | -0.29 | [-0.39, | -0.19] | -5.49 | 320 | < .001*** | -0.16 | | [-0.26, | -0.05] | -2.83 | 320 | .054 |  |
|  | BIF | 0.07 | [-0.04, | 0.17] | 1.19 | 320 | .701 | 0.1 | | [-0.01, | 0.20] | 1.71 | 320 | .789 |  |
|  | Holistic Tendency | 0.27 | [ 0.16, | 0.37] | 4.95 | 320 | < .001*** | 0.27 | | [ 0.17, | 0.37] | 5.05 | 320 | < .001*** |  |
|  | Hexaco_HonHum | -0.25 | [-0.35, | -0.15] | -4.65 | 320 | < .001*** | -0.04 | | [-0.15, | 0.07] | -0.65 | 320 | > .999 |  |
|  | Hexaco_Emotion | 0.06 | [-0.05, | 0.17] | 1.11 | 320 | .701 | 0.06 | | [-0.05, | 0.17] | 1.09 | 320 | > .999 |  |
|  | Hexaco_Extra | <0.01 | [-0.11, | 0.11] | -0.05 | 320 | .963 | 0.04 | | [-0.07, | 0.15] | 0.68 | 320 | > .999 |  |
|  | Hexaco_Agree | -0.14 | [-0.24, | -0.03] | -2.51 | 320 | .075 | 0.07 | | [-0.04, | 0.17] | 1.18 | 320 | > .999 |  |
|  | Hexaco_Cons | -0.34 | [-0.43, | -0.24] | -6.5 | 320 | < .001*** | 0.01 | | [-0.10, | 0.12] | 0.23 | 320 | > .999 |  |
|  | Hexaco_Open | -0.12 | [-0.23, | -0.01] | -2.22 | 320 | .109 | 0.12 | | [ 0.01, | 0.23] | 2.18 | 320 | .300 |  |
|  | Psychological distance | 0.46 | [ 0.37, | 0.54] | 9.28 | 320 | < .001*** | 0.21 | | [ 0.10, | 0.31] | 3.85 | 320 | .002** |  |
|  | Temporal closeness | 0.3 | [ 0.20, | 0.39] | 5.59 | 320 | < .001*** | 0.28 | | [ 0.17, | 0.37] | 5.15 | 320 | < .001*** |  |
|  | Closeness to conflict person | 0.23 | [ 0.13, | 0.34] | 4.31 | 320 | < .001*** | 0.09 | | [-0.02, | 0.19] | 1.53 | 320 | > .999 |  |
|  | Reliance on emotion/intuition | 0.38 | [ 0.28, | 0.47] | 7.25 | 320 | < .001*** | 0.32 | | [ 0.21, | 0.41] | 5.96 | 320 | < .001*** |  |
|  | Impulsivity assessment | 0.29 | [ 0.18, | 0.39] | 5.38 | 320 | < .001*** | 0.06 | | [-0.05, | 0.17] | 1.06 | 320 | > .999 |  |
|  | State-level outsider view | 0.45 | [ 0.35, | 0.53] | 8.9 | 320 | < .001*** | 0.29 | | [ 0.19, | 0.39] | 5.45 | 320 | < .001*** |  |
|  | *State-level wisdom* | 0.46 | [ 0.37, | 0.54] | 9.25 | 320 | < .001*** | 0.43 | | [ 0.34, | 0.51] | 8.5 | 320 | < .001*** |  |
|  | Intellectual humility | 0.47 | [ 0.38, | 0.55] | 9.6 | 320 | < .001*** | 0.33 | | [ 0.23, | 0.43] | 6.32 | 320 | < .001*** |  |
|  | Others' perspectives | 0.43 | [ 0.34, | 0.52] | 8.6 | 320 | < .001*** | 0.36 | | [ 0.26, | 0.45] | 6.84 | 320 | < .001*** |  |
|  | Multiple views | 0.36 | [ 0.27, | 0.46] | 6.99 | 320 | < .001*** | 0.39 | | [ 0.29, | 0.48] | 7.55 | 320 | < .001*** |  |
|  | Compromise | 0.33 | [ 0.23, | 0.43] | 6.32 | 320 | < .001*** | 0.43 | | [ 0.33, | 0.51] | 8.48 | 320 | < .001*** |  |
| UK | REI_Analytic | 0.03 | [-0.10, | 0.16] | 0.49 | 236 | > .999 | 0.09 | | [-0.03, | 0.22] | 1.45 | 236 | > .999 |  |
|  | REI_Intuition | 0.11 | [-0.02, | 0.24] | 1.72 | 236 | > .999 | 0.07 | | [-0.05, | 0.20] | 1.13 | 236 | > .999 |  |
|  | REI_Imagination | 0.15 | [ 0.02, | 0.27] | 2.29 | 236 | .344 | <0.01 | | [ 0.10, | 0.34] | 3.54 | 236 | .011* |  |
|  | REI_Emotionality | 0.08 | [-0.05, | 0.20] | 1.17 | 236 | > .999 | 01 | | [-0.12, | 0.13] | 0.1 | 236 | > .999 |  |
|  | CRT | -0.06 | [-0.19, | 0.07] | -0.91 | 236 | > .999 | -0.07 | | [-0.19, | 0.06] | -1.05 | 236 | > .999 |  |
|  | BIF | -0.02 | [-0.14, | 0.11] | -0.24 | 236 | > .999 | 0.08 | | [-0.04, | 0.21] | 1.29 | 236 | > .999 |  |
|  | Holistic Tendency | 0.05 | [-0.08, | 0.17] | 0.71 | 236 | > .999 | 0.23 | | [ 0.10, | 0.35] | 3.60 | 236 | .009** |  |
|  | Hexaco_HonHum | -0.04 | [-0.17, | 0.08] | -0.69 | 236 | > .999 | -0.05 | | [-0.18, | 0.07] | -0.83 | 236 | > .999 |  |
|  | Hexaco_Emotion | 0.05 | [-0.07, | 0.18] | 0.84 | 236 | > .999 | 0.08 | | [-0.04, | 0.21] | 1.30 | 236 | > .999 |  |
|  | Hexaco_Extra | -0.09 | [-0.21, | 0.04] | -1.34 | 236 | > .999 | 0.06 | | [-0.07, | 0.19] | 0.96 | 236 | > .999 |  |
|  | Hexaco_Agree | -0.07 | [-0.20, | 0.06] | -1.1 | 236 | > .999 | 0.04 | | [-0.09, | 0.16] | 0.55 | 236 | > .999 |  |
|  | Hexaco_Cons | 0.01 | [-0.11, | 0.14] | 0.23 | 236 | > .999 | 0.16 | | [ 0.03, | 0.28] | 2.45 | 236 | .225 |  |
|  | Hexaco_Open | 0.08 | [-0.05, | 0.21] | 1.24 | 236 | > .999 | 0.18 | | [ 0.05, | 0.30] | 2.75 | 236 | .110 |  |
|  | Psychological distance | 0.16 | [ 0.03, | 0.28] | 2.5 | 236 | .218 | 0.13 | | [ 0.00, | 0.25] | 2.05 | 236 | .503 |  |
|  | Temporal closeness | 0.24 | [ 0.11, | 0.35] | 3.72 | 236 | .006** | 0.2 | | [ 0.07, | 0.32] | 3.13 | 236 | .035* |  |
|  | Closeness to conflict person | 0.17 | [ 0.05, | 0.30] | 2.73 | 236 | .136 | 0.04 | | [-0.09, | 0.16] | 0.57 | 236 | > .999 |  |
|  | Reliance on emotion/intuition | 0.27 | [ 0.14, | 0.38] | 4.24 | 236 | < .001*** | <0.01 | | [ 0.14, | 0.37] | 4.13 | 236 | .001** |  |
|  | Impulsivity assessment | 0.22 | [ 0.10, | 0.34] | 3.5 | 236 | .012* | <0.01 | | [-0.14, | 0.12] | -0.14 | 236 | > .999 |  |
|  | State-level outsider view | 0.18 | [ 0.05, | 0.30] | 2.82 | 236 | .110 | 0.15 | | [ 0.02, | 0.27] | 2.29 | 236 | .301 |  |
|  | *State-level wisdom* | 0.17 | [ 0.04, | 0.29] | 2.57 | 236 | .203 | 0.22 | | [ 0.10, | 0.34] | 3.48 | 236 | .013* |  |
|  | Intellectual humility | 0.16 | [ 0.04, | 0.28] | 2.53 | 236 | .218 | 0.16 | | [ 0.03, | 0.28] | 2.44 | 236 | .225 |  |
|  | Others' perspectives | 0.13 | [ 0.00, | 0.25] | 1.96 | 236 | .709 | 0.2 | | [ 0.08, | 0.32] | 3.20 | 236 | .029* |  |
|  | Multiple views | 0.11 | [-0.02, | 0.23] | 1.63 | 236 | > .999 | 0.17 | | [ 0.05, | 0.29] | 2.71 | 236 | .115 |  |
|  | Compromise | 0.16 | [ 0.03, | 0.28] | 2.51 | 236 | .218 | 0.21 | | [ 0.08, | 0.32] | 3.22 | 236 | .029* |  |

*Note. p-value adjustment method: Holm (1979)*

## Figure S6: Bifactor model of abstract and concrete construal in Study 1


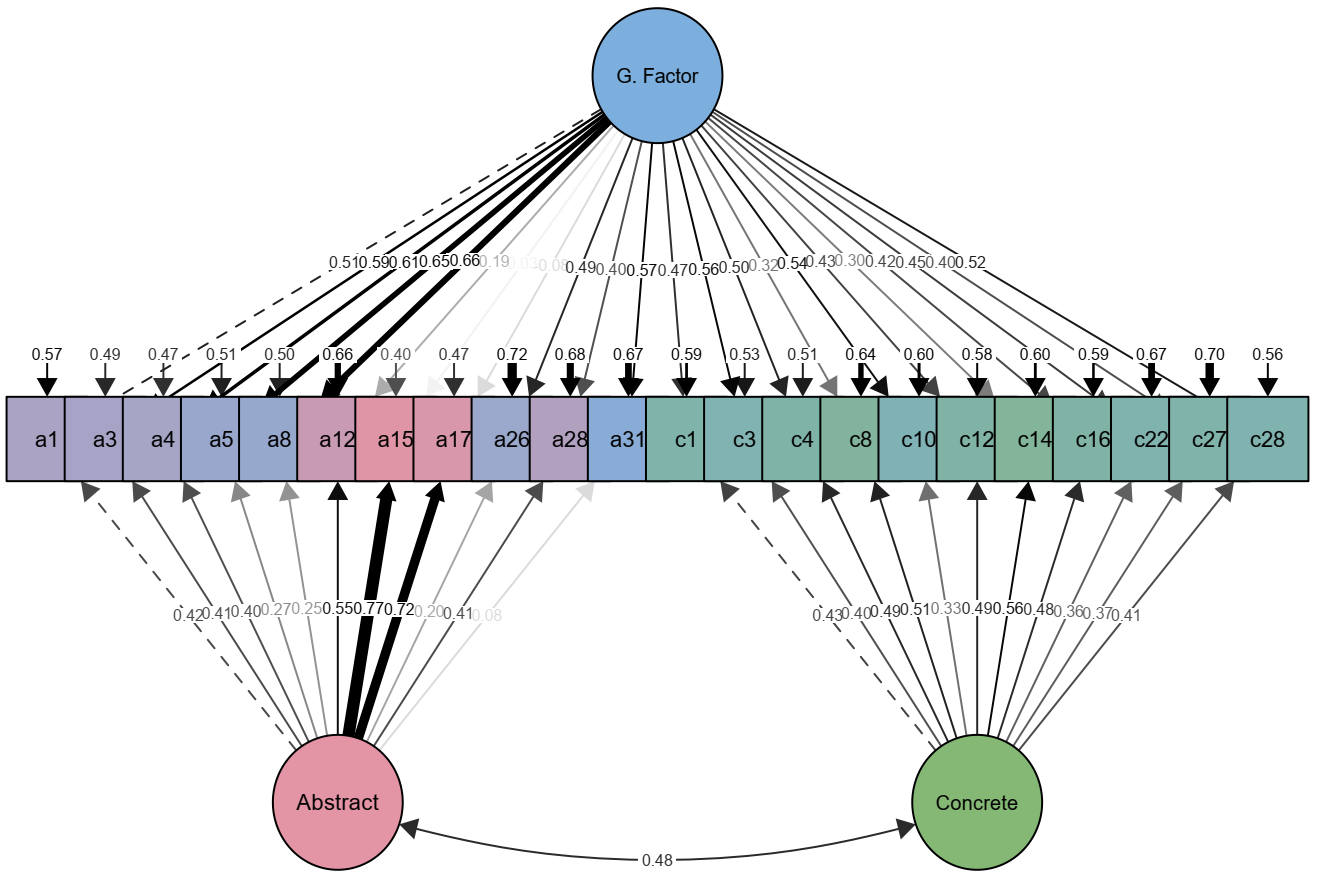


Path diagram of the bifactor model with 22 items (a = items from the abstract pool; c = items from the abstract pool). G. Factor = general factor. Item coloring and line thickness correspond to strength of association with respective construal factors and the general factor. For ease of interpretation, we include standardized parameter estimates.

## Figure S7: Effects of abstract and concrete construal on holism and wisdom in Study 1


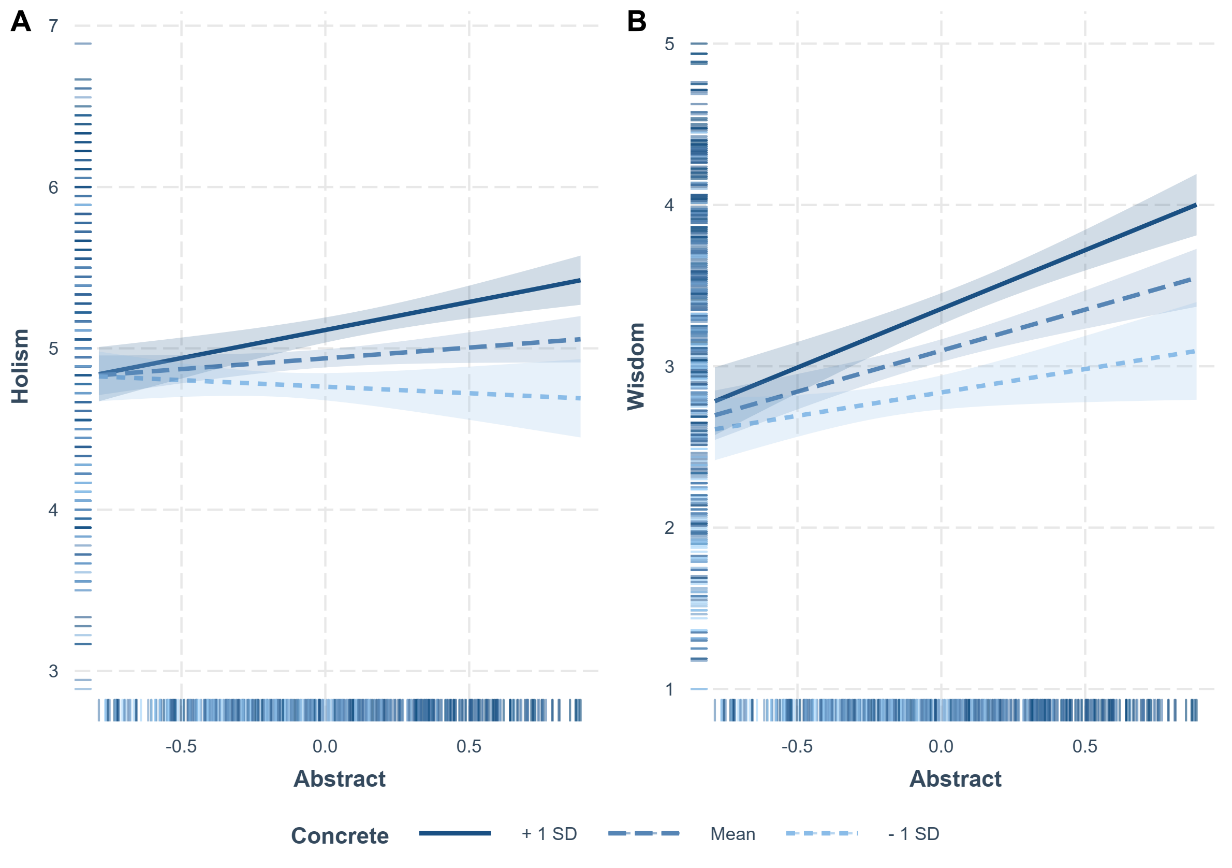


Simple slopes showing the nature of interaction between abstract and concrete construal (factor scores on a standardized scale) and respective dependent variable. Plot A: Holistic tendencies; Plot B: Mental features of wisdom. Rugs show distributions of the predictor (abstract construal - bottom) and response variable (left).

# Study 2

## Relatability of events

Participants rated all events as relatable (see Figure S6 in the supplement), with five scenarios rated as somewhat relatable (2.86 < *M*s ≤ 3.32; 2.68 < 95% *CI*s ≤ 3.76), and one scenario in wave 2 as slightly relatable (*M* = 2.10; 95% *CI* [1.94, 2.26]), omnibus *F*(5,1326.1) = 54.40, *p* < .001. The six events varied in reported abstractness (2.87 < *M*s ≤ 3.74; 2.77 < 95% *CI*s ≤ 3.84), *F*(5, 1316.1) = 55.52, *p* < .001, and to a lesser extent in concreteness (3.56 < *M*s ≤ 3.92; 3.49 < 95% *CI*s ≤ 4.02), *F*(5, 1312.2) = 15.96, *p*s < .001. Replicating Study 1 and the pilot studies, participants overall reported significantly more concreteness than abstractness when reflecting on social challenges, *t*(2902) = 15.79, *p* < .001. This effect was pronounced for five out of six events, 2.30< *ts*(2896) ≤ 19.17, .021 < *p*s ≤ .001. For one event (“difficulty to reconnect with friends”) we observed a reversal, *t*(2896) = 3.02, *p* = .003. See supplementary Figures S8-S10 for event-specific means. Similarly, participants tended to choose concrete rather than abstract strategies among the top eight that came to their mind first, *t* (290.41) = 5.99, *p* < .001, dovetails with results of SACCS-based responses.

## Effects in the strategy ordering task

In the Strategy Ordering Task, people selected more concrete (0) than abstract (1) strategies, *B* = -0.143, *SE* = 0.024, *z* = 5.90, *p* < .001. Notably, as supplementary Figure S12 shows, people scoring higher on wisdom (+1*SD* below the mean) were more likely to select abstract strategies for the first position and remained at chance thereafter, *B* (linear effect of rank) = -0.002, *SE* = 0.011, *z* = 0.15, *p* = .879. In contrast, people scoring low on wisdom (-1*SD* below the mean) were equally likely to select abstract and concrete strategies for the first position and were more likely to select concrete construal for later positions, *B (*linear effect of rank) = -0.023, *SE* = 0.011, *z* = 2.07, *p* = .038.

## Effects in disbalance on different features of wisdom

We performed separate linear mixed effect analyses with grand-mean-centered person’s average disbalance score across situations (i.e., person’s trait-level effects) and person-centered disbalance score (i.e., person’s state-level effect; situation-specific deviation from one’s average) as simultaneous predictors, and each feature of wisdom as a dependent variable, with responses nested in participants. Our aim was to examine if (dis)balance is particularly aligned with dialecticism and open-mindedness, compared to other features of wisdom such as intellectual humility and compromise. Results indicated compare effects of trait-level disbalance on intellectual humility, *B* = -0.143, *SE* = 0.029, *t*(800.30) = 5.46, *p* < .001, others’ perspectives, *B* = -0.138, *SE* = 0.028, *t*(798.62) = 4.98, *p* < .001, multiple views, *B* = -0.142, *SE* = 0.029, *t*(801.32) = 4.85, *p* < .001, and compromise, *B* = -0.124, *SE* = 0.029, *t*(800.28) = 4.31, *p* < .001. Similarly, state-level effects were equally non-significant, intellectual humility: *t* < .84, *ns.*; others’ perspectives: *t*(800.34) = 1.68, *p*  = .093, multiple views: *t* < .33, *ns.*, and compromise: *t* < .20, *ns.* We conclude that the effects of disbalance were comparable across different features of wisdom we assessed.

## Effects in switching on different features of wisdom

We performed separate linear mixed effect analyses with grand-mean-centered person’s average switching score across situations (i.e., person’s trait-level effects) and person-centered switching score (i.e., person’s state-level effect; situation-specific deviation from one’s average) as simultaneous predictors, and each feature of wisdom as a dependent variable, with responses nested in participants. Our aim was to examine if switching is particularly aligned with dialecticism and open-mindedness, compared to other features of wisdom such as intellectual humility and compromise. Results indicated compare effects of trait-level disbalance on intellectual humility, *B* = 0.121, *SE* = 0.027, *t*(798.41) = 4.40, *p* < .001, others’ perspectives, *B* = 0.112, *SE* = 0.028, *t*(797.55) = 4.03, *p* < .001, multiple views, *B* = 0.120, *SE* = 0.026, *t*(798.97) = 4.64, *p* < .001, and compromise, *B* = 0.076, *SE* = 0.025, *t*(798.10) = 3.05, *p* = .002. Similarly, state-level effects were equally non-significant, intellectual humility: *t* (799.90) = 1.14, *p =* .254; others’ perspectives: *t*(798.65) = 1.44, *p*  = .150, multiple views: *t* < .23, *ns.*, and compromise: *t* < .18, *ns.* We conclude that the effects of switching were comparable across different features of wisdom we assessed.

## Table S22: Descriptive statistics, zero-order correlations, and measures of intra-individual and within-wave stability of key measures in Study 2.

|  | *M* | *SD* | ICC (person) | ICC (event) | 1 | 2 | 3 | 4 | 5 | | a | | b | | c | | d | | 6 | |  |
| --- | --- | --- | --- | --- | --- | --- | --- | --- | --- | --- | --- | --- | --- | --- | --- | --- | --- | --- | --- | --- | --- |
| 1. Abstract | 3.42 | 0.85 | 0.37 | 0.11 |  |  |  |  | |  | |  | |  | |  | |  | |  | |
| 2. Concrete | 3.76 | 0.75 | 0.51 | 0.03 | 0.45 |  |  |  | |  | |  | |  | |  | |  | |  | |
| 3. Disbalance | 17 | 13.38 | 0.12 | 0.09 | -0.25 | -0.04 |  |  | |  | |  | |  | |  | |  | |  | |
| 4. Construal Switching | 2.94 | 1.49 | 0.14 | 0.04 | 0.19 | 0.03 | -0.57 |  | |  | |  | |  | |  | |  | |  | |
| 5. Wisdom Index | 3.14 | 0.86 | 0.47 | 0.06 | 0.44 | 0.44 | -0.15 | 0.14 | |  | |  | |  | |  | |  | |  | |
| a. Intellectual humility | 3.02 | 1.04 | 0.41 | 0.05 | 0.38 | 0.36 | -0.12 | 0.11 | | 0.87 | |  | |  | |  | |  | |  | |
| b. Others’ perspectives | 3.17 | 1.13 | 0.28 | 0.12 | 0.34 | 0.33 | -0.14 | 0.11 | | 0.86 | | 0.74 | |  | |  | |  | |  | |
| c. Multiple views | 3.26 | 0.99 | 0.36 | 0.03 | 0.36 | 0.40 | -0.12 | 0.11 | | 0.79 | | 0.6 | | 0.57 | |  | |  | |  | |
| d. Compromise | 3.4 | 0.97 | 0.33 | 0.05 | 0.31 | 0.41 | -0.11 | 0.07 | | 0.82 | | 0.61 | | 0.68 | | 0.64 | |  | |  | |
| 6. Self-transcendence | 2.83 | 1.13 | 0.48 | 0.03 | 0.40 | 0.30 | -0.15 | 0.16 | | 0.74 | | 0.6 | | 0.48 | | 0.46 | | 0.45 | |  | |
| 7. Relatability | 3.04 | 1.40 | 0.19 | 0.13 | 0.35 | 0.20 | 0.02 | < .01 | | 0.24 | | 0.19 | | 0.21 | | 0.22 | | 0.21 | | 0.16 | |

*Note*. *ICC* = intra-class correlations from a random intercept linear mixed model without fixed effects (null model).

## Figure S8: Relatability of events in Study 2


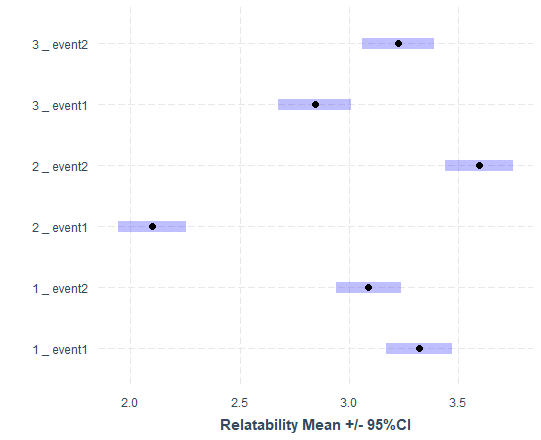


Estimated means of relatability for events across measurement waves 1-3 in Study 2. Participants rated relatability on a 5-point scale (1= *not at all*, 2 = *slightly*, 3 = *somewhat*, 4 = *moderately*, 5 = *very much***).** Though participants reported at least some reliability for all events, reliability was lowest for the first event of the second measurement wave (“husband’s repeated infidelity”), 7.17 < post-hoc pairwise *t*s ≤ 15.06, *p*s < .001, and greatest for the second event of the same wave (“difficulty to reconnect with friends”), 2.82 < post-hoc pairwise *t*s ≤ 15.06, .055 < *p*s < .001.

## Figure S9: Abstractness of events in Study 2


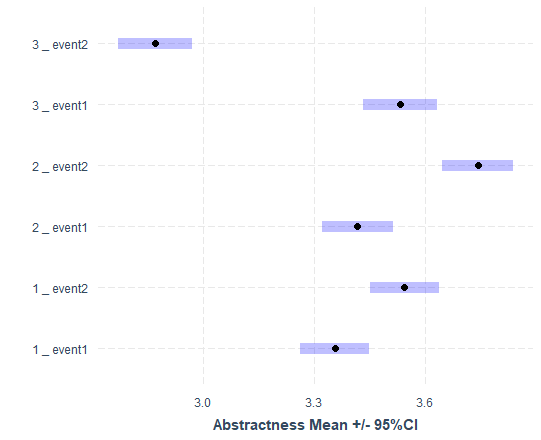


Estimated means of abstractness for events across measurement waves 1-3 in Study 2. Participants reported abstractness above the mid-point of the scale (3) for all events, except for the second event during the third measurement wave (“angry nurse at mom’s hospital”). For this event, participants reported lower abstractness compared to all other events, 8.86 < post-hoc pairwise *t*s ≤ 15.63, *p*s < .001. Participants reported highest degree of abstractness for the second event during wave 2 (“difficulty to reconnect with friends”), 3.78 < post-hoc pairwise *t*s ≤ 15.63, .002 < *p*s < .001.

## Figure S10: Concreteness of events in Study 2


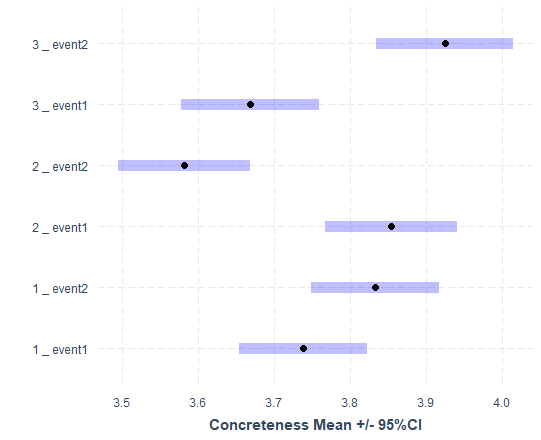


Estimated means of concreteness for events across measurement waves 1-3 in Study 2. Participants reported concreteness above the mid-point of the scale (3) for all events. Participants reported lower concreteness compared to all other events for the second event during wave 2 (“difficulty to reconnect with friends”), 1.19 < post-hoc pairwise *t*s ≤ 6.18, .411 < *p*s < .001. Participants reported highest degree of concreteness for the second event during wave 3 (“angry nurse at mom’s hospital”), 1.53 < post-hoc pairwise *t*s ≤ 7.43, .644 < *p*s < .001.

## Figure S11: Average wisdom for each event in Study 2


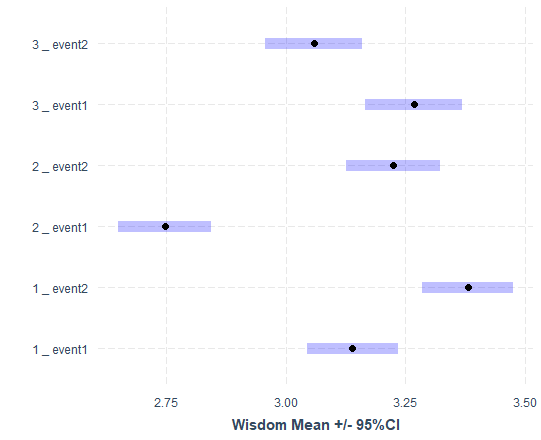


Estimated means of wisdom for events across measurement waves 1-3 in Study 2. Participants reported wisdom above the mid-point of the scale (3) for all events, except for the first event during the second measurement wave (“husband’s repeated infidelity” – scores below the midpoint) and the second event of the last measurement wave (“angry nurse at mom’s hospital” – 95% *CI* includes the midpoint). Participants reported lower wisdom compared to all other events for the first event during the second measurement wave (“husband’s repeated infidelity”), 5.86 < post-hoc pairwise *t*s ≤ 12.66, *p*s < .001. Participants reported highest degree of wisdom for the second event during wave 1 (“lost friend on facebook”), 2.17 < post-hoc pairwise *t*s ≤ 12.66, .252 < *p*s < .001.

## Figure S12: Likelihood of selecting abstract strategies for different ranks in Study 2


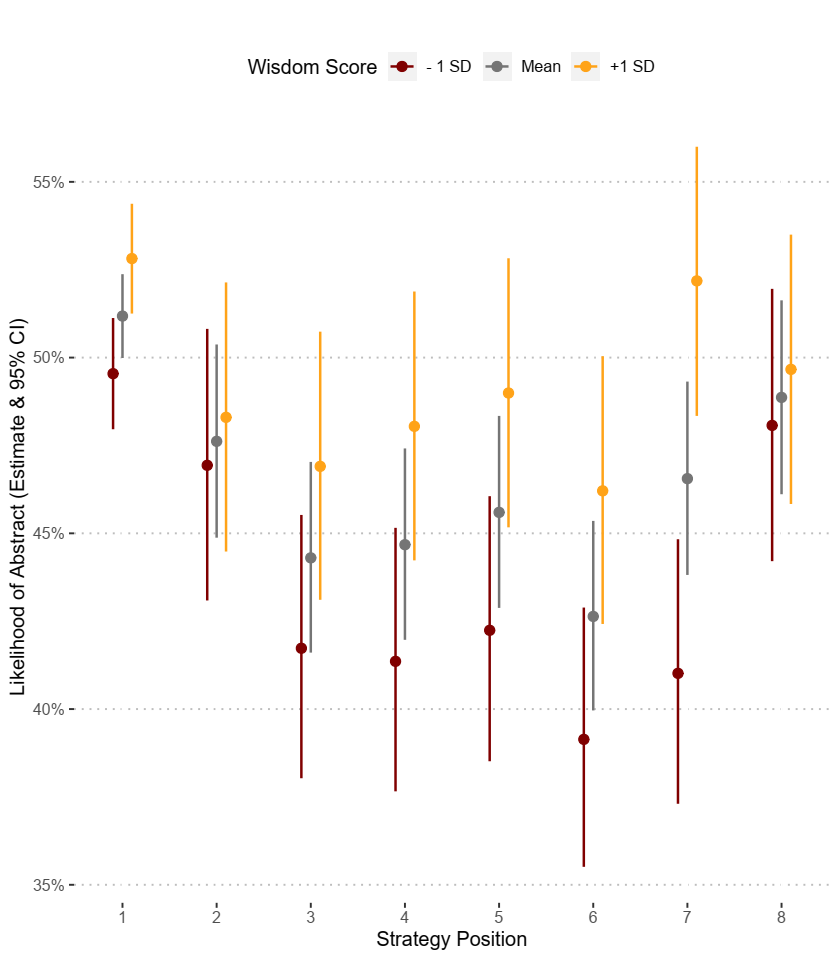


Likelihood of choosing abstract strategies for each position in the Strategy Ordering Task in Study 2, estimated for participants at 1 *SD* below the average wisdom score, at average wisdom, and at 1 *SD* below the average wisdom score. Estimates from a generalized logistic mixed model with position, wisdom score, and their interaction predicting likelihood of choosing abstract (1) rather than concrete (0). Most people start at parity (equally likely to choose abstract and concrete) and move toward concrete. This trend is more pronounced among low wisdom participants, while high wisdom participants remain equally likely to choose abstract and concrete for each position.

## Figure S13: SACCS-based ratings of construal and construal disbalance in Study 2


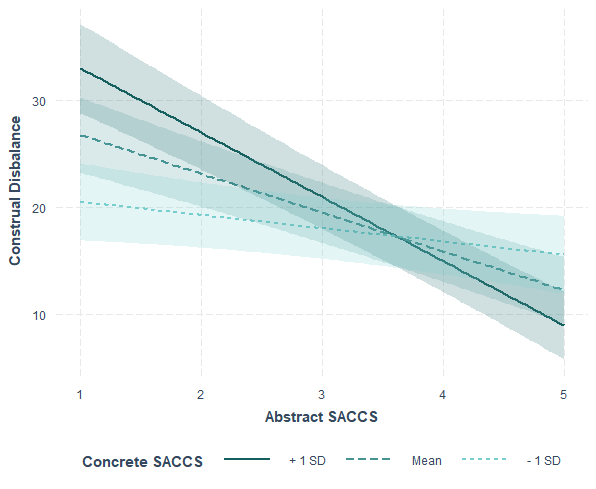


Estimated disbalance (% deviations from parity—50 %) between abstract and concrete construal strategies as a function of abstract and concrete construal on the SACCS (*S*ituation-specific *A*bstract and *C*oncrete *C*onstrual *S*cale) in Study 2. Simple slope estimates and 95% confidence band at the mean concreteness, as well as 1 *SD* above and below the mean. Participants who reported higher scores on both abstract and concrete SACCS sub-scales were significantly less likely to show disbalance between abstract and concrete strategies in their reflections, abstract X concrete interaction, *t* (1385.35) = 7.21, *p* < .001. Simple slope shows that abstractness weakly predicted less disbalance when concreteness was low (- 1 *SD*), *B* = -1.233, *SE* = 0.565, *t* = 2.181, *p* = .029, and strongly predicted less disbalance when concreteness was high (+ 1 *SD*), *B* = -5.991, *SE* = 0.559, *t* = 10.726, *p* < .001.

## Figure S14: SACCS-based ratings of construal and construal switching in Study 2


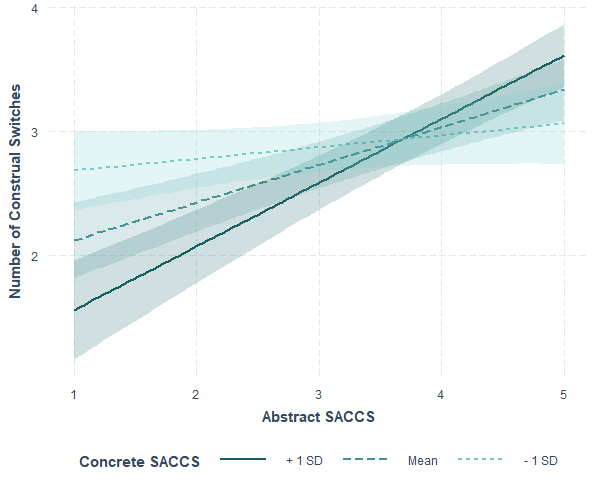


Estimated number of switches between abstract and concrete construal strategies in the order of reflection as a function of abstract and concrete construal on the SACCS (*S*ituation-specific *A*bstract and *C*oncrete *C*onstrual *S*cale) in Study 2. Simple slope estimates and 95% confidence band at the mean concreteness, as well as 1 *SD* above and below the mean. Participants who reported higher scores on both abstract and concrete SACCS sub-scales were significantly more likely to show switching between abstract and concrete strategies in their reflections, abstract X concrete interaction, *t*(1433.38) = 5.49, *p* < .001. Simple slope shows that abstractness did not significantly predict switching when concreteness was low (- 1 *SD*), *B* = 0.095, *SE* = 0.065, *t* = 1.463, *p* = .144, but did predict switching when concreteness was high (+ 1 *SD*), *B* = 0.514, *SE* = 0.064, *t* = 7.997, *p* < .001.

# Supplementary References

1. Grossmann, I. *et al.* The Science of Wisdom in a Polarized World: Knowns and Unknowns. *Psychol. Inq.* **31**, 103–133 (2020).

2. Caruso, E. M., Gilbert, D. T. & Wilson, T. D. A Wrinkle in Time: Asymmetric Valuation of Past and Future Events. *Psychol. Sci.* **19**, 796–801 (2008).

3. Kane, J., Van Boven, L. & McGraw, A. P. Prototypical prospection: future events are more prototypically represented and simulated than past events: Prototypical prospection. *Eur. J. Soc. Psychol.* **42**, 354–362 (2012).

4. Freitas, A. L., Gollwitzer, P. & Trope, Y. The influence of abstract and concrete mindsets on anticipating and guiding others’ self-regulatory efforts. *J. Exp. Soc. Psychol.* **40**, 739–752 (2004).

5. Liberman, N. & Trope, Y. The role of feasibility and desirability considerations in near and distant future decisions: A test of temporal construal theory. *J. Pers. Soc. Psychol.* **75**, 5–18 (1998).

6. Eyal, T., Liberman, N., Trope, Y. & Walther, E. The Pros and Cons of Temporally Near and Distant Action. *J. Pers. Soc. Psychol.* **86**, 781–795 (2004).

7. Brienza, J. P., Kung, F. Y. H., Santos, H. C., Bobocel, D. R. & Grossmann, I. Wisdom, bias, and balance: Toward a process-sensitive measurement of wisdom-related cognition. *J. Pers. Soc. Psychol.* **115**, 1093–1126 (2018).

8. Grossmann, I., Oakes, H. & Santos, H. C. Wise reasoning benefits from emodiversity, irrespective of emotional intensity. *J. Exp. Psychol. Gen.* **148**, 805–823 (2019).

9. Vallacher, R. R. & Wegner, D. M. Action Identification Theory. in *Handbook of Theories of Social Psychology* vol. 1 199–203 (2012).

10. Grossmann, I. & Kross, E. The impact of culture on adaptive versus maladaptive self-reflection. *Psychol. Sci.* **21**, 1150–1157 (2010).

11. Ayduk, Ö. & Kross, E. From a distance: Implications of spontaneous self-distancing for adaptive self-reflection. *J. Pers. Soc. Psychol.* **98**, 809–829 (2010).

12. Sanchez, A. A wrinkle in time: Temporal landmarks and the relation between temporal distance and abstraction. (2018).

13. Schwarzer, G. Meta‐Analysis in R. in *Systematic Reviews in Health Research* (eds. Egger, M., Higgins, J. P. T. & Davey Smith, G.) 510–534 (Wiley, 2022). doi:10.1002/9781119099369.ch26.

14. Eyal, T., Sagristano, M. D., Trope, Y., Liberman, N. & Chaiken, S. When values matter: Expressing values in behavioral intentions for the near vs. distant future. *J. Exp. Soc. Psychol.* **45**, 35–43 (2009).

15. Tabachnick, B. G. & Fidell, L. S. *Experimental designs using ANOVA*. (Thomson/Brooks/Cole, 2007).

16. Hu, L. & Bentler, P. M. Cutoff criteria for fit indexes in covariance structure analysis: Conventional criteria versus new alternatives. *Struct. Equ. Model. Multidiscip. J.* **6**, 1–55 (1999).

17. Meyers, L. S., Gamst, G. & Guarino. *Meyers, L. S., Gamst, G., & Guarino, A. J. Applied multivariate research: Design and interpretation*. (Sage, 2006).

1. Pilot Study D included two additional items concerning motivation (“how motivated would you be to make the best possible choice” and “how important would it be to you to make the right choice”) on a *Not Important at all* (1) to *Very Important* (7) scale. Abstract and concrete construals both positively contributed to motivation to make a good decision, yet only concrete construal reached statistical significance, *B* = 0.448, *SE* = 0.056, *t* = 7.65, *p* < .001. [↑](#footnote-ref-1)
